# Tracing electron density changes in langbeinite under pressure

**DOI:** 10.1107/S2052252521012628

**Published:** 2021-12-23

**Authors:** Roman Gajda, Dongzhou Zhang, Jan Parafiniuk, Przemysław Dera, Krzysztof Woźniak

**Affiliations:** a Biological and Chemical Research Centre, Department of Chemistry, University of Warsaw, Żwirki i Wigury 101, Warszawa 02-089, Poland; b APS, University of Chicago, 9700 S. Cass Avenue, Building 434A, Argonne, IL 60439, USA; c Institute of Geochemistry, Mineralogy and Petrology, Department of Geology, University of Warsaw, Żwirki i Wigury 93, Warszawa 02-089, Poland; d Hawaii Institute of Geophysics and Planetology, School of Ocean and Earth Science and Technology, University of Hawaii at Manoa, 1680 East West Road, Honolulu, Hawaii 96822, USA

**Keywords:** high pressure, electron density, theoretical structure factors

## Abstract

Pressure is well known to dramatically alter physical properties and chemical behaviour of materials, much of which is due to the changes in chemical bonding that accompany compression. Though it is relatively easy to comprehend this correlation in the discontinuous compression regime, where phase transformations take place, understanding of the more subtle continuous compression effects is a far greater challenge, requiring insight into the finest details of electron density redistribution. In this study, a detailed examination of quantitative electron density redistribution in the mineral langbeinite was conducted at high pressure. Langbeinite is a potassium magnesium sulfate mineral with the chemical formula [K_2_Mg_2_(SO_4_)_3_], and crystallizes in the isometric tetartoidal (cubic) system. The mineral is an ore of potassium, occurs in marine evaporite deposits in association with carnallite, halite and sylvite, and gives its name to the langbeinites, a family of substances with the same cubic structure, a tetrahedral anion, and large and small cations. Single-crystal X-ray diffraction data for langbeinite have been collected at ambient pressure and at 1 GPa using a combination of in-house and synchrotron techniques. Experiments were complemented by theoretical calculations within the pressure range up to 40 GPa. On the basis of changes in structural and thermal parameters, all ions in the langbeinite structure can be grouped into ‘soft’ (potassium cations and oxygens) and ‘hard’ (sulfur and magnesium). This analysis emphasizes the importance of atomic basins as a convenient tool to analyse the redistribution of electron density under external stimuli such as pressure or temperature. Gradual reduction of completeness of experimental data accompanying compression did not significantly reduce the quality of structural, electronic and thermal parameters obtained in experimental quantitative charge density analysis.

## Abstract 2

Experimental and theoretical charge density investigations performed for langbeinite allow for detailed examination of the redistribution of electron density under pressure both within atomic basins as well as at atomic positions.

## 1. Introduction

Establishing a detailed picture of electron density evolution as a function of depth in rock-forming mineral phases is absolutely crucial for the development of quantitative models, allowing us to predict chemical and physical transformations involved in major geological processes taking place in the deep interiors of Earth and other extraterrestrial planets, as well as in ore formation. All these processes can be traced at subatomic levels of detail, achievable by combining experimental charge density studies and crystal structure investigations under extreme conditions. This work is a continuation of our previous study on experimental quantitative electron density determination in grossular under 1 GPa pressure (Gajda *et al.*, 2020 ▸), which demonstrated the feasibility of such analysis but was not able to identify clear pressure-induced effects at the electron density level.

**Figure 1:**
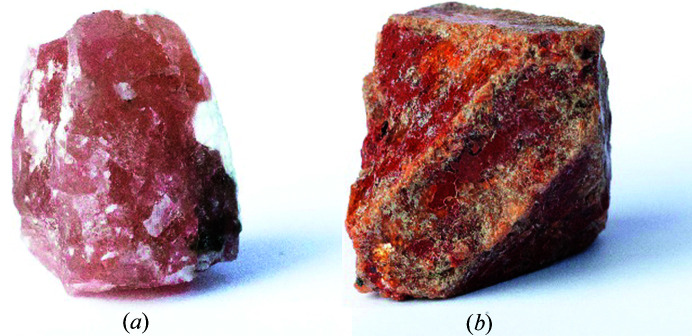
(*a*) Langbeinite [K_2_Mg_2_(SO_4_)_3_], Kalusa (Kałusz), Ukraine, *ca* 3 × 1.5 × 1.2 cm, small pieces of this big crystal are studied in this work. (*b*) Langbeinite crystal replaced by sylvine, Carlsbad Potash Mining District, New Mexico, USA, *ca* 3 × 1.5 × 1.2 cm.

Here we focus our attention on an another model mineral, langbeinite [K_2_Mg_2_(SO_4_)_3_] (Speer & Salje, 1986 ▸; Mereiter, 1979 ▸), hereafter abbreviated Lb (see Figs. 1 ▸ and 2 ▸). The mineral was first described from salt deposits in Wilhelmshall, Halberstadt, Saxony-Anhalt, Germany and named after Adalbert Lanbein (1834–1894), a German chemist and technical director of Concordia Chemische Fabrik, Leopoldshall (Zuckschwerdt, 1891). Langbeinite usually occurs as granular, massive aggregates, sometimes it also forms nodules or single crystals scattered in the mass of potash salt. Euhedral crystals are rare, tetrahedron in shape and up to a few centimetres (Fig. 1 ▸). They are colourless or pale pink, yellow, green and grey. The mineral has no cleavage, a conchoidal fracture, a hardness of 3,5–4 and is slowly soluble in water. It is piezoelectric and has interesting magnetic properties (Oelkrug *et al.*, 1988 ▸). Langbeinite is a significant constituent of marine salt deposits but it does not crystallize directly during the evaporation of sea water and is formed during the subsequent transformations of potash beds. The mineral is of economic importance and its more important deposits are located in Germany, England, Austria, Penjab (India) and New Mexico (USA). The research material for our study comes from the Kalusa (formerly Kałusz) deposit in Ukraine. Langbeinite has also been noted from volcanic fumaroles in Kamchatka, Russia and from high-temperature exhalations in burning coal dumps in the Upper Silesia coal basin (Parafiniuk & Siuda, 2021 ▸). Langbeinite is composed of relatively light atoms/ions such as potassium, magnesium, sulfur and oxygen, and belongs to a wider group of sulfate minerals with similar structures. Other minerals in the langbeinite group include efremovite [(NH_4_)_2_Mg_2_(SO_4_)_3_] (Shimobayashi *et al.*, 2011 ▸) and manganolangbeinite [K_2_Mn_2_(SO_4_)_3_] (Yamada *et al.*, 1981 ▸), which all crystallize in the space group *P*2_1_3 of the regular (cubic) crystal system. High symmetry of these minerals allows the collection of diffraction data with high completeness and redundancy, even when the sample is enclosed in a diamond anvil cell (DAC). Some other similar minerals such as picromerite [MgK_2_(SO_4_)_2_ *x*(H_2_O)_6_] (Kannan & Viswamitra, 1965 ▸) and leonite [K_2_Mg(SO_4_)_2_ *x*(H_2_O)_4_] (Hertweck *et al.*, 2001 ▸, 2003 ▸) crystallize in the monoclinic crystal system which is more challenging for quantitative charge density investigations.

Lb also denotes a specific group of structures in the Inorganic Crystal Structure Database (ICSD) (Bergerhoff *et al.*, 1983 ▸) which itself has many variants containing different anions and cations. Examples of the Lb-like structures include compounds containing cadmium [K_2_Cd_2_(SO_4_)_3_] (Percival *et al.*, 1989 ▸), zinc [K_2_Zn_2_(SO_4_)_3_] (Moriyoshi & Itoh, 1996 ▸) or calcium [K_2_Ca_2_(SO_4_)_3_] (Xu *et al.*, 2017 ▸) as the metal cation. In others, the SO_4_ group is substituted with PO_4_, for example: [Ba_3_V_4_(PO_4_)_6_] (Droß & Glaum, 2004 ▸), [Rb_2_FeZr(PO_4_)_3_] (Trubach *et al.*, 2004 ▸), [KBaFe_2_(PO_4_)_3_] (Battle *et al.*, 1986 ▸), [KBaCr_2_(PO_4_)_3_] (Battle *et al.*, 1988 ▸).

Establishing experimental charge density distributions from high-pressure X-ray diffraction data is a significant challenge. However, some examples of experimental electron density determined for crystals under high pressure have already been published. This has been achieved so far either for pure elements (Li *et al.*, 2015 ▸) or for inorganic compounds using maximum entropy methods (Yamanaka *et al.*, 2009 ▸). An interesting application of experimental charge density studies to the molecular organic crystal *syn*-1,6:8,13-bis­carbonyl­[14]annulene under pressure has also been published by Macchi and co-workers (Casati *et al.*, 2016 ▸). Additionally, challenges associated with charge density analysis in crystals at high pressure have been discussed by Casati *et al.* (2017 ▸) and others (Diamond & Jeanloz, 2020 ▸; Yamada *et al.*, 2018 ▸). An interesting continuation of the above studies utilizing the idea of the transferable aspherical atom approach (TAAM) has been recently published by Milašinović *et al.* (2021 ▸).

### 1.1. Experimental determinations of quantitative electron density distributions (EDDs) in crystals

In order to obtain an accurate EDD, one needs to collect accurate, precise and complete X-ray diffraction data up to a sufficiently high resolution, including the high diffraction angle data range. By ‘high-resolution X-ray measurements’ we mean such measurements for which (sin θ/λ)_max_ is close to or even larger than 1.1 Å^−1^. High resolution is a must because the large number of parameters of the multipolar model of electron density requires many more unique observations to secure a proper observation/parameter ratio. The other reason is that high-resolution reflections are strongly associated with nuclear positions and particularly with temperature factors of atoms (atomic displacement parameters – ADPs) whereas the low diffraction angle reflections contribute mostly to the valence electron density. It is also a common belief that an X-ray dataset should be sufficiently complete to avoid systematic effects in the refinement. Once reflection intensities are measured, we can refine not only the spherical electron density model (independent atom model – IAM), but also some more advanced aspherical electron density models. The most common aspherical quantitative experimental charge density model is based on a finite spherical harmonic expansion of the electronic part of the charge distribution around each atomic centre. Such an atomic expansion is called a pseudoatom and the molecular electron distribution at any point in a crystal is the sum of all the pseudoatomic densities. In the most commonly used formalism of Hansen and coworkers (Hansen & Coppens, 1978 ▸; Koritsanszky & Coppens, 2001 ▸), the pseudoatom electron density is defined by

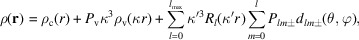

where ρ_c_(*r*) and ρ_v_(*r*) are spherical core and valence densities, respectively. The third term contains the sum of the angular functions *d*_*lm*±_(θ,φ) to take into account aspherical deformations. The angular functions *d*_*lm*±_(θ,φ) are real spherical harmonic functions. The coefficients *P*_v_ and *P*_*lm*±_ are populations for the valence and deformation density multipoles, respectively. κ and κ’ are scaling parameters introduced to make valence and deformation densities expand or contract. In the Hansen–Coppens formalism, *P*_v_, *P*_*lm*±_, κ and κ′ are refineable parameters together with the atomic coordinates and thermal coefficients. Least-squares refinements are performed against the measured intensities *F* ^2^(*hkl*) of reflections obtained by single-crystal X-ray diffraction. This requires a data resolution of 0.45–0.50 Å. Starting atomic coordinates and anisotropic displacement parameters are taken from the ordinary spherical refinement stage and freely refined. For more information on multipole refinement and topological analysis of electron density see the supporting information.

### 1.2. Topological analysis of electron density

Once quantitative EDD in minerals is established, different methods of electron density partitioning can be used to analyse properties of the studied systems. One of the most popular is atoms-in-molecules (AIM) theory (Bader, 1994 ▸). AIM theory (Popelier, 1996 ▸) offers a self-consistent way of partitioning any molecular system into its atomic fragments, deduced from the first principles of quantum mechanics and Schwinger’s principle of stationary action (Coppens *et al.*, 1979 ▸). In AIM theory, the many-electron system is separated into subsystems (atomic basins) by *zero-flux surfaces* (ZFSs) that satisfy the following condition for every point on the surface: **n**∇ρ(**r**) = 0, where ∇ρ(**r**) is the gradient vector field of the molecular electron density, **r** is a point on the zero-flux surface that separates two fragments and **n** is the vector normal to the surface at that point. Further analysis of the gradient vector field of electron density results in localization of the extremes of the electron density by finding ‘critical points’ (CPs) at which the following equation applies: ∇ρ(**r**_CP_) = 0. Particularly useful are bond critical points (BCPs) – the weakest points in bonds which define their properties. Integrating these properties over the atomic basins is one of the cornerstones of AIM theory because it yields valuable information such as integrated charges and the volumes of atoms/ions, their energies, electronic populations as well as higher multiple moments polarizabilities *etc.* (Ángyán *et al.*, 1994 ▸). More information on quantum topological approaches is given in the supporting information.

### 1.3. Charge density studies in mineralogy

There is limited research describing experimental charge densities in minerals from X-ray data (without high-pressure data). So far, the following minerals have been studied by applying multipolar refinements: mesolite [Na_2_Ca_2_(Al_2_Si_3_O_10_)3·*x*8H_2_O] (Kirfel & Gibbs, 2000 ▸), natrolite [Na_2_Al_2_Si_3_O_10_·*x*2H_2_O] (Kirfel & Gibbs, 2000 ▸; Ghermani *et al.*, 1996 ▸), scolecite [CaAl_2_Si_3_O_10_·*x*3H_2_O] (Kirfel & Gibbs, 2000 ▸; Kuntzinger *et al.*, 1998 ▸), phenakite [Be_2_SiO_4_] (Downs & Gibbs, 1987 ▸; Tsirelson *et al.*, 1990 ▸), coesite [SiO_2_] (Gibbs *et al.*, 2003 ▸), stishovite SiO_2_ (Kirfel *et al.*, 2001 ▸), rutile [TiO_2_] (Restori *et al.*, 1987 ▸; Jiang *et al.*, 2003 ▸), azurite [Cu_3_(CO_3_)_2_(OH)_2_] (Belokoneva *et al.*, 2001 ▸), cuprite [Cu_2_O] (Restori & Schwarzenbach, 1986 ▸) topaz [Al_2_(SiO_4_)F_2_] (Ivanov *et al.*, 1998 ▸), dioptase [Cu_6_(Si_6_O_18_)·*x*6H_2_O] (Belokoneva *et al.*, 2002 ▸), triphylite [LiFePO_4_] (Streltsov *et al.*, 1993 ▸), fluorite [CaF_2_] (Stachowicz *et al.*, 2017 ▸) and grossular (Gajda *et al.*, 2020 ▸). The above mentioned minerals were mostly investigated at so called ambient conditions, meaning ambient pressure as well as room temperature.

Previously published papers have approached the problem from different directions. For example, the EDD of danburite (CaB_2_Si_2_O_8_) was investigated experimentally and published in 1992 (Downs & Swope, 1992 ▸). Ten years later charge density for the same danburite was obtained also on the basis of quantum mechanical calculations (Luaña *et al.*, 2003 ▸). The second work showed the utility of comparing experimental high-resolution electron density studies and results obtained on the basis of theoretical calculations. For some minerals [*e.g.* spodumene (LiAlSi_2_O_6_)], only theoretical charge density results are available (Prencipe *et al.*, 2003 ▸; Kuntzinger & Ghermani, 1999 ▸). For others such as datolite (CaBOHSiO_4_), the Hansen–Coppens multipole model and Bader’s topological analysis – just on the basis of experimental data – were reported (Ivanov & Belokoneva, 2007 ▸). However, approaches that combine experiment and theory are becoming more common [*e.g.* clinopyroxene (LiGaSi_2_O_6_; Bianchi *et al.*, 2007 ▸)]. Many authors also use maximum entropy methods as a tool to describe the topology of electron density in minerals. An example of such an approach is also work on clinopyroxene (Merli & Cámara, 2003 ▸). Most of the papers describing EDD published to date discuss charge density under ambient conditions [*e.g.* diopside (MgCaSi_2_O_6_; Bianchi *et al.*, 2005 ▸)]. However, recent papers describing changes in EDD for inorganic compounds such as CaSi_2_O_5_ as function of pressure have appeared [theoretical simulations (Yu *et al.*, 2013 ▸)].

However, the older reports are less reliable because of the generally poor quality diffraction data and severe methodological approximations applied at that time. Notably, there are likely to be a few more charge density studies of minerals accomplished in Russia (particularly, in the very active group of Professor Tsirelson in Moscow) published in Russian journals, but are not covered by the common literature databases.

### 1.4. Aims of this work

The main goal of our work has been to quantify the influence of pressure on experimental charge density in the Lb structure, and also examine the geometrical and thermal motion parameters of ions present in this structure. However, this work also includes a substantial methodological effort, focused on discussing differences in the properties of charge density distributions obtained for complete and incomplete high-resolution X-ray diffraction *hkl* datasets, differences between experimental and theoretical charge density distributions obtained on the basis of experimental data, and theoretical dynamic structure factors. We also examined whether the wavelength of X-ray radiation used for data collection influences the final distributions of electron density, and compared the results of charge density studies obtained at two different wavelengths (Ag *K*α and Mo *K*α) at ambient pressure.

## 2. Experimental

Multipole refinement of electron density requires not only high resolution of the X-ray diffraction data, but also high completeness. In the case of high-pressure experiments, where a sample is placed inside access-constraining DAC, it is difficult to fulfil both of these requirements. However, by employing shorter X-ray wavelengths, more reflections can be collected over a wider *d*-spacing range. Synchrotron facilities in particular seem to be quite useful for collecting suitable X-ray data [*e.g.* forsterite (Mg_2_SiO_4_; Kirfel *et al.*, 2005 ▸)]. For this reason, our leading experiment was conducted at a synchrotron facility. In our synchrotron experiment, four small pieces of big single crystals (Fig. 1 ▸) of Lb were placed in the DAC (see Fig. 3 ▸), and because of their complementary orientations, each of them made unique contributions when the final concatenated *hkl* file was constructed. We were able to collect data with a resolution up to 0.45 Å. Despite the fact that we used a DAC with a relatively wide opening angle (120°), crystals with high cubic symmetry and four single pieces of crystals in different orientations (finally, only three of them were taken into account), the completeness was only 89% for the highest-angle reflections. On the basis of these data, multipole refinement of electron density according to the Hansen–Coppens multipole model was conducted and experimental EDD was obtained. To obtain a reference EDD at ambient pressure, a high-resolution X-ray diffraction dataset was collected using our in-house diffractometers. Two data collections were conducted, with Mo and Ag X-ray sources. Additionally, to extend the pressure range available in DAC and to check whether the complete *hkl* dataset had a significant influence on the final results of the multipole refinement of electron density, theoretical calculations for the Lb structure under pressures up to 40 GPa were also performed.

### 2.1. Synchrotron facility: data collection details

Single-crystal X-ray diffraction data collection at high pressure was carried out at the experimental 13-BM-C station at the Advanced Photon Source, Argonne National Laboratory (Zhang *et al.*, 2017 ▸). The X-ray beam was monochromated with a silicon 311 crystal to a wavelength of 0.434 Å with 1 eV bandwidth. A Kirkpatrick–Baez mirror system was used to obtain a vertical × horizontal focus spot size of 18 × 12 µm, measured as the full width at half-maximum (see Fig. 3 ▸). A Pilatus3 1M detector (Dectris) was placed about 200 mm from the sample, and LaB_6_ powder at room temperature and pressure was used to calibrate the distance and tilting of the detector. The sample was placed on the rotation centre of the diffractometer, and was aligned by scanning the sample chamber absorption profile with X-rays. A membrane pressure controller was used to adjust the pressure remotely, and the pressure was determined by ruby fluorescence (Dewaele *et al.*, 2008 ▸). Four crystals of Lb with random orientations were loaded at the centre of the sample chamber of a One20 DAC (Easylab), and helium was used as the pressure-transmitting medium (Rivers *et al.*, 2008 ▸). The diffraction data were collected with a six-circle kappa geometry diffractometer (Newport). The diffraction patterns were collected at two different 2θ angles (0 and 30°) and covered the whole opening angle range of the DAC from φ = −55° to φ = 55°. Each diffraction frame covered a φ range of 0.5°. At 2θ = 0°, the exposure time was 5 s per frame, and at 2θ = 30°, the exposure time was 20 s per frame. For each crystal, diffraction patterns were collected at two different χ angles (0 and 90°). The diffraction images at different settings were merged and reduced to *hkl* files using the *APEX3* software package (Bruker). Basic experimental data corresponding with this measurement are presented in Table 1 ▸ and labelled APS_exp.

The synchrotron data collection was conducted at 1 GPa pressure. The list of differences between the synchrotron and in-house experiments were: shorter wavelength (λ = 0.434 Å), different values of pressure and poorer access to the Ewald sphere (missing reflections).

### 2.2. In-house measurements

The in-house data collection was conducted at ambient temperature and pressure and was used as a reference benchmark for the synchrotron experiments. An Lb specimen was mounted on top of a thin glass capillary with a tiny amount of ep­oxy resin. An optimal data collection strategy, yielding a complete dataset up to the above resolution, was calculated with the *CrysAlisPRO* software (CrysAlis Pro, 2014 ▸). One measurement was conducted using a diffractometer equipped with a Mo X-ray microfocus source (*K*α λ = 0.7107 Å) and a second with Ag X-ray microfocus source (*K*α λ = 0.5609 Å). Basic information about both measurements is presented in Table 1 ▸. Data collected with Mo radiation is named Mo_exp and that with Ag radiation named Ag_exp. All the reflections, as defined in the reciprocal lattice by the Ewald sphere, were collected up to 0.4 Å resolution. An extended table of data collection and crystal structure parameters is available in the supporting information.

### 2.3. Data reduction

Data reduction for all the frames collected was performed using the *CrysAlisPRO* software (Rigaku Oxford Diffraction, 2015 ▸). Next, the structures were solved and refined with *ShelXS* (Sheldrick, 2008 ▸) and *ShelXL* (Sheldrick, 2015 ▸), respectively, within the *Olex2* suite (Dolomanov *et al.*, 2009 ▸). Then, the intensities for each of the measurements were merged using *Sortav* (Blessing, 1995 ▸) implemented in the *WinGX* program suite (Farrugia, 2012 ▸). Such merged reflection intensity data were subsequently used as an input for the *XD2016* program (Volkov *et al.*, 2016 ▸).

### 2.4. Multipole refinements

The structure of Lb, solved and refined at the IAM level using *SHELX*-97, served as a starting point for the refinement of the Hansen–Coppens multipole model of electron density. Refinements against the data collected at the APS synchrotron facility and the data collected in-house, as well as data on the basis of theoretical calculations (denoted theor_0 and theor_1) were processed in the same way. The refinement was conducted on *F* ^2^ without any weighting parameters. In each case, the model included a multipole expansion up to the *l* = 4 level (hexadecapoles). Because K and Mg atoms/ions occupy special positions, only appropriate symmetry-specific multipoles were taken into account. Moreover, special constraints for atomic thermal parameters were used. Each model was refined in seven steps, including refinement of kappa coefficients. Details of these steps and their order are reported in the supporting information. The scale factor was refined in each step. No significant correlation between the refined parameters was observed.

### 2.5. Theoretical calculations

In order to prepare the input data for multipole refinement based on theoretical calculations as the first step, the crystal structure of Lb was optimized in *CRYSTAL17* (Dovesi *et al.*, 2017 ▸, 2018 ▸). On the basis of the results of this optimization, so called dynamic structure factors were calculated (Erba *et al.*, 2013 ▸). The list of proper structure factors was built in such a way that it contains the structure factors up to resolution of 0.45 Å (which corresponds to the resolution of APS_exp) with 100% completeness (no missing reflections as in the case of APS_exp). Because *CRYSTAL17* allows for optimization under different pressure conditions, the set of dynamic structure factors under different values of pressure was obtained. Such prepared structure factors were used as input data for multipole refinement which was proceeded in the same way as refinements for experimental datasets. There are two major differences between the experimental and theoretical data which should be underlined here. Firstly, the experimental *hkl* file contains experimentally measured intensities of reflections (*I* ≃ *F* ^2^) with values of the sample standard deviations. Whereas theoretical *hkl* files contain calculated dynamic structure factors (*F*), and because they are calculated, they have no sample standard deviation values, although they were used in optimization in the same form as the experimental values.

## 3. Results

### 3.1. Influence of high pressure on structure, thermal motions and EDD in langbeinite

#### 3.1.1. Crystal structure

**Figure 2:**
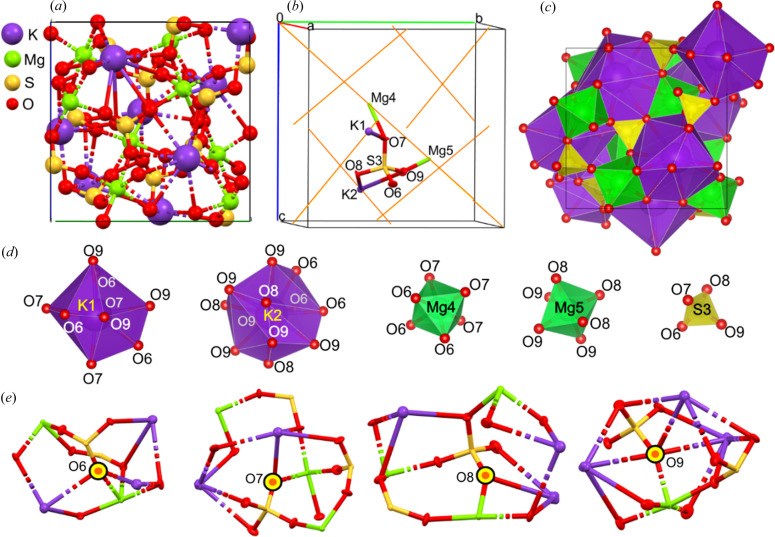
Langbeinite. (*a*) Atomic arrangement within the unit cell. (*b*) Atomic labels and ADP ellipsoids for atoms/ions defining the independent part of the unit cell (orange lines depict threefold axes). (*c*) Visualization of the polyhedral model of the langbeinite structure: SO_4_, MgO_6_ and KO_12_ polyhedra and the local neighbourhood of oxygen atoms/ions. The Wyckoff positions of K and Mg cations are *a*3. Other ions are placed at general positions. (*d*) Separated polyhedral of particular ions. (*e*) Local neighbourhood of oxygen ions/atoms.

**Figure 3:**
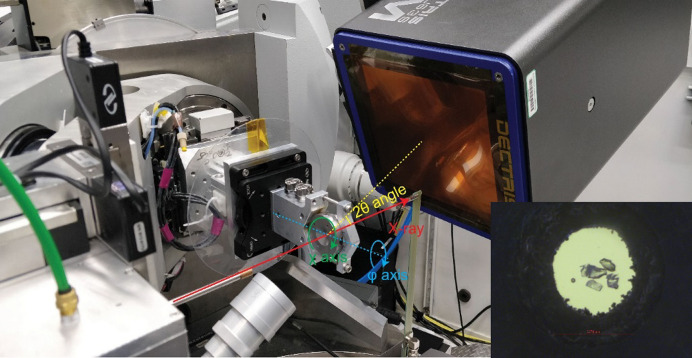
Geometry of the high-pressure single-crystal diffraction experiment at APS 13-BM-C. X-ray direction together with φ, χ and 2θ angles are labelled. Inset: image of the DAC sample chamber after He gas loading, showing four Lb crystals and one ruby sphere. The diameter of the sample chamber was 0.274 mm, indicated by the red scale bar.

**Figure 4:**
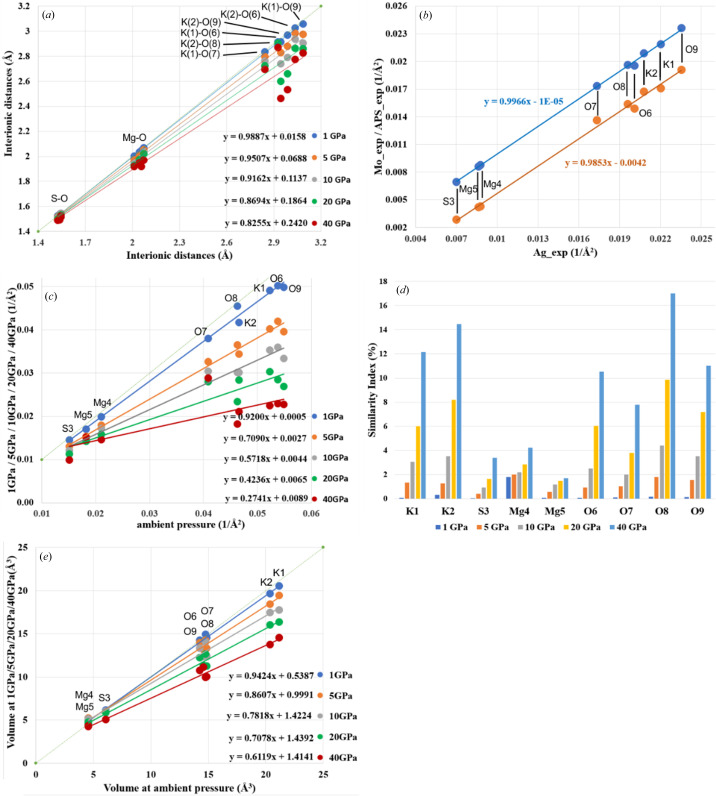
(*a*) Interionic distances as a function of pressure (data correspond with Table S7). (*b*) Average eigenvalues for specific ADPs obtained on the basis of synchrotron measurements and in-house measurements. Blue dots – relation between Ag_exp and Mo_exp, orange dots – relation between Ag_exp and APS_exp. (*c*) Average eigenvalues for specific ADPs obtained on the basis of theoretical calculations. (*d*) Similarity index calculated for ADPs obtained on the basis of theoretical dynamic structure factors. (*e*) Volumes of integrated atomic basins under pressure *versus* integrated ambient atomic basins – all from theoretical calculations.

Lb crystallizes in the cubic space group *P*2_1_3 (Figs. 1 ▸ and 2 ▸). The crystal structure of Lb is composed of SO_4_ tetrahedra and MgO_6_ octahedra. Potassium cations, which were placed in the voids between these polyhedra, are surrounded by oxygen anions (see Fig. 2 ▸). Due to significant geometric distortion of the K coordination environment, it can be considered a deformed KO_12_ icosahedron. Although the polyhedra seem to completely fill the space [Fig. 2 ▸(*c*)], this schematic presentation is not optimal, when the topology of EDD must be described, as polyhedra are not the best representations of the actual electron density, since they contain only a part of the atomic electron density of the central atom/ion and small parts of electron density of the corner ions/atoms. For this reason we focused on the analysis of atomic basins instead. Note that refinement of aspherical models of electron density also results in more precise geometric parameters (bond lengths and valence angles) than is possible in conventional spherical atom refinements. Tables containing information about interatomic distances for Mo_exp, Ag_exp and APS_exp are presented in Table S6 of the supporting information. One can use the ambient-pressure data as the reference and find relations between particular parameters when the pressure is changed. For example, in Fig. 4 ▸(*a*) linear relations between the ambient-pressure interatomic distances and the interatomic distances at elevated pressures are shown. The slopes of these relations can be interpreted as a change in the resistance of this structure to the increasing pressure. Of course, the KO_12_ polyhedra are the most sensitive to changes of pressure, whereas the SO_4_ tetrahedron is the least deformable by pressure. Different types of interionic contacts give different responses to pressure increases. The most resistant to pressure are the S—O contacts, which are the shortest and are considered to be covalent bonds. A 40 GPa pressure causes shrinking of only about 1.5–3.0% in length. Slightly more susceptible to compression are the Mg—O contacts, which undergo changes in length by 4.6–6.8% at 40 GPa compared with ambient conditions. The most significant changes as a function of pressure were observed for the K—O contacts, which shorten by up to 16.3% at 40 GPa (both the relative and absolute changes are the largest observed here).

#### 3.1.2. Anisotropic displacement parameters

It is widely known that lower temperatures result in smaller values for ADPs. Also, the higher the pressure, the smaller the thermal ellipsoids. Although the effect seems to be similar (reduction of ADPs), the mechanism is slightly different (reduction of the vibrational energy *versus* contraction of the potential wall). The consequence is that temperature reduces the atomic displacements as well as the anharmonic components, whereas pressure reduces the displacements but not the anharmonicity. To check whether a pressure of 1 GPa is large enough to cause any significant changes of ADPs in Lb, we compared the thermal ellipsoids for all experiments. Specific tables containing the values of ADPs resulting from the refinement of the Mo_exp, Ag_exp and APS_exp data as well as tables containing principal diagonal components of ADPs obtained in both theoretical and experimental investigations are available in Tables S8–S10 of the supporting information. Fig. 4(*b*) presents charts comparing the relationship between the isotropic ADPs [the average eigenvalues of the ADP matrices of particular ions/atoms at ambient pressure and 1 GPa, see Fig. 4 ▸(*b*)]. The relationship between the average eigenvalues for specific ADPs obtained from synchrotron measurements and in-house measurements Ag_exp (*x* axis) and Mo_exp (*y* axis; blue dots) and between the isotropic ADP values for Ag_exp (*x* axis) and APS_exp (*y* axis; orange dots) are shown in Fig. 4 ▸(*b*). From the data analys in this figure, we can see two clear trends. First, the eigenvalues obtained for the synchrotron measurements at 1 GPa are systematically lower for all types of atoms compared with the corresponding eigenvalues obtained for the in-house measurements at ambient pressure. So the difference of 1 GPa is sufficient to observe this effect. The difference is *ca* 0.004 (1 Å^−2^) and, as a result, those eigenvalues are smaller by about 60% for S3, 50% for Mg4 and Mg5, and about 20% for K and O ions. The second trend is visible when we expand our range of investigation and include the results for theoretical calculations [see Fig. 4 ▸(*c*)]. As we can see, the ADPs of some atoms/ions are more affected by increasing pressure than the ADPs of others. The ‘hard/resistant’ atoms include sulfur and magnesium. The list of ‘soft/sensitive’ atoms includes oxygen and potassium ions. One can also note that the increase of pressure generates non-uniform changes of ADPs. In relative terms (per unit of pressure), the changes of ADPs are largest when pressure is close to the ambient value. On the other hand, when pressure is already high the compressibility of ions is of course smaller. One can see this from a regular decrease in the slope of the linear relations shown in Fig. 4 ▸(*c*) by relating the slope to pressure. This is a consequence of the shape of EOS function.

A convenient method of ADP comparison is the similarity index (see Table S11) (Whitten & Spackman, 2006 ▸). This indicator was introduced to characterize the degree of agreement between ADPs obtained using different approaches (1 and 2). It can be defined in terms of the overlap between the two probability density functions in direct space. It is convenient to transform the ADP tensor *U* into a Cartesian system, and for two compared tensors *U*_1_ and *U*_2_, the equation has the form (Whitten & Spackman, 2006 ▸):

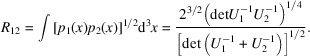

Probability density functions are normalized, so as a result, for *U*_1_ = *U*_2_, the similarity index *R*_12_ = 1.0. To underline subtle differences and present them as percentages between two compared ADPs we used the similarity index in the form

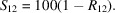

By analysing the data in Table S11, we observed that the *S*_12_ index is surprisingly small for K cations but significant (up to almost 40%) for oxygen anions. The X-ray data from APS_exp (1 GPa) are different from those obtained in both in-house experiments (ambient pressure). However, despite the rather good correlation between ADPs of both in-house measurements [see Fig. 4 ▸(*b*)], the similarity index for them still shows differences up to 20% for some oxygen anions, whereas for the rest of ions the ADPs are almost identical. This highlights the importance of proper description and deconvolution of thermal motion of ions from electron distribution effects. The use of pressure (*ca* 1 GPa) doubles the values of similarity indexes which effectively illustrates the scale of compressibility of ions close to ambient pressure. No doubt this is the valence electron density of the oxygen atoms/ions which are the softest and can be easily relocated by pressure.

In the case of theoretical calculations, differences between particular ADPs are much smaller despite the larger pressure range used in the computations (see Table S10). One reason could be the fact that experimental measurements were absolutely independent from one another whereas the theoretical results output from one calculation at a given pressure value was used as input for the next higher pressure value calculations. So despite shrinking ADPs, their orientations in space were more or less conserved. Taking into consideration the results depicted in Fig. 4 ▸(*d*), we see once again that the similarity index shows the most significant changes in the case of potassium and oxygen ions, whereas for sulfur and magnesium the changes are as much as four times smaller. Atomic volumes [Fig. 4 ▸(*e*)] are discussed in the next section.

### 3.2. Influence of high pressure on EDD in langbeinite

#### 3.2.1. Electron density parameters

In order to compare parameters of EDDs, we will discuss the properties at bond critical points (BCPs), net charge of atoms/ions, volumes of integrated atomic basins, maps of the laplacian of electron density, total electron density and deformation electron density. The crucial experiment in this context is our high-resolution, high-pressure measurement at the synchrotron facility abbreviated to APS_exp. We will relate results obtained at high pressure to those obtained at ambient pressure denoted Ag_exp and Mo_exp.

Table S12 presents the values of electron density and laplacian of electron density at (3,−1)-type BCPs. The amount of electron density (ρ) at the BCPs for all interionic contacts in the Lb structure resulting from the APS_exp data look indistinguishable from results obtained on the basis of the in-house experiments. Surprisingly, there is one outlier among the ∇^2^_ρ_ values which is observed for the S(3)—O(8) interaction [2.9 e^−^ Å^−5^ *versus* −15.3 e^−^ Å^−5^/−13.0 e^−^ Å^−5^]. Relatively high values of ρ and ∇^2^_ρ_ < 0 suggest that the S—O bonds can be considered as shared-shell interactions (covalent and polar bonds), whereas Mg⋯O for which ∇^2^_ρ_ > 0 and values of ρ are small as closed-shell interactions (the ionic ones). Results for Ag_exp and Mo_exp are comparable but not identical. In the case of ρ, the values obtained for the Mg—O bonds seem to be systematically larger for Ag_exp than for Mo_exp [with the exception of the S(3)—O(6) contact]. Although numerical values of electron density ρ and Laplacian ∇^2^_ρ_ presented in Table S12 are not absolutely identical for compared experiments, they are very similar, especially taking into account the estimated standard deviation error.

Because the results of the in-house experiments are based on 100% complete X-ray data and data obtained from APS, to the APS_exp, which has only a 89% completeness, an additional column has been added in Table S12 and Table 2 ▸. The ‘Ag_exp (APS completeness)’ column shows results for the in-house Ag_exp after the removal of these reflections which are missing in the synchrotron data. In other words, in this attempt the completeness of the original dataset was adjusted to be exactly the same as the synchrotron data. Note that differences in ρ are at the second position after the coma, and are smaller than the standard deviation of ρ. Differences in Laplacian values are more noticeable, but only significant for S(3)—O(6) and S(3)—O(8) contacts. Atomic charges differ at the second decimal position similarly as for atomic volumes. This shows that the cutting off of the data did not significantly change the results quantitatively or qualitatively. We will discuss the effect of deteriorating completeness on the final results of refinement in the last section of this work.

Table 2 ▸ contains values of the net atomic charge and atomic volumes after integration of the atomic/ionic basins. The most striking differences were observed for O(9) which is an interlink between three potassium polyhedra. Its charge, −2.0, is *ca* 0.4^−^ bigger than the average value for the in-house experiments. Additionally, a significant discrepancy was observed for K(2). Its charge is about 0.45 smaller than for the averaged in-house data. Except for the noted outliers other results were directly comparable. The sum of all atomic charges per unit cell after atomic basin integration is close to 0 and the sum of atomic volumes is equal to the volume of the unit cell within the level of errors. For the unit-cell volume, the sums of atomic volumes differed from the volumes of the original cells by *ca* −0.7, 0.7, −1.2 and −2.4% for Ag_exp, Ag_exp (APS completeness), Mo_exp and APS_exp data, respectively.

When comparing particular values of charge and atomic volume for Ag_exp and Mo_exp, one can see that the biggest atomic basins had potassium cations located in cavities between the SO_4_ and MgO_6_ polyhedra, whereas Mg and S, surrounded closely by oxygen atoms, have four times smaller atomic basins. The atomic charge at the potassium cation is *ca* +1 whereas that of the magnesium cation is significantly larger. The fact that both potassium cations have a slightly different charge and not all oxygen anions had the same charge is associated with the different interactions of these ions in the crystal lattice of Lb. The K(1) polyhedron is formed by nine oxygen anions [O(6), O(7), O(9) and symmetry-related oxygens], whereas the K(2) void is surrounded by 12 oxygen anions (see Fig. 2 ▸). On the other hand, the position of O(9) is a bridging position between three potassium voids and as a consequence its charge is significantly different from the charge of the other oxygen atoms/ions.

#### 3.2.2. Dynamic structure factors and theoretical models of electron density

Because of the limitations resulting from DAC construction, sometimes it is not possible to obtain experimental data suitable for experimental charge density analysis for high-pressure values. Thus theoretical calculations which can mimic particular pressure effects could be compelling. In this work experimental data were complemented with theoretical calculations for six pressure values. Two of them, ambient pressure and 1 GPa, correspond with experimental measurements, the other allowed us to widen the investigation slightly beyond current experimental limits.

In Table S13, the values of selected properties at BCPs resulting from theoretical calculations are presented. The values of ρ and the laplacian were calculated with the use of the *XDPROP* module of *XD*. To avoid repetition, the table combining results for BCPs for experimental and theoretical data is presented in the supporting information. In Table 3 ▸, the volumes of integrated atomic basins and net atomic charges within these basins are presented.

**Figure 5:**
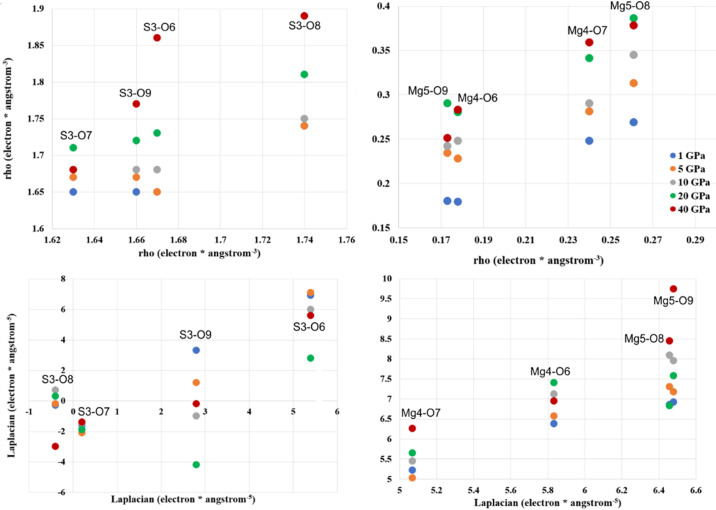
Laplacian and ρ as a function of pressure for S—O and Mg—O contacts at the (3, −1) BCPs for theoretical calculations.

When we consider ρ and ∇^2^_ρ_ for Mg⋯O-type interactions, we can see that these values correspond very well with the experimental values (Table S12). However, the values of the laplacian in the case of S⋯O are no longer very negative, thus indicating that these ‘covalent’ bonds are becoming more ionic (Fig. 5 ▸).

Table S14 presents the values of the net atomic charge and volumes obtained after integration over atomic basins. The values of the net atomic charge do not change significantly within the investigated pressure range. Charge does not change monotonically as it is dependent on changing the electronegativity of ions/atoms under pressure or/and the transfer of charge among ions under pressure. The deviation of total charge per unit cell in the whole pressure range varied investigated between +0.64 and −0.44 which seems to be irrelevant taking into consideration that the unit cell of Lb contains 824 electrons (384 valence electrons to refine by the multipole model), so the deviation of about 1 electron from neutrality is just 0.12% of all the electrons in the unit cell.

We observe a similar situation for atomic basins as a function of pressure [see Fig. 4 ▸(*e*)] as for the ADP values [see Fig. 4 ▸(*c*)]. There is group of ‘hard’ atoms such as sulfur and magnesium whose volumes are quite resistant to pressure change and a group of ‘soft’ atoms/ions such as oxygen and potassium whose atomic basins are noticeably decreasing under pressure.

As we see from Fig. 4 ▸(*e*) and Table 3 ▸, the volumes of atomic basins decrease as a function of pressure as expected. In addition to the numerical values of many point parameters presented in the tables above, maps of these parameters could be helpful to compare results of EDD obtained in different ways and detect detailed differences. Here we would like to discuss three types of such 2D maps: maps of total electron density, maps of the Laplacian and maps of deformation density. These maps are presented in Figs. S2 and S3. Two areas are depicted on the maps: a vicinity of the S cation (plane defined by the S cation and two neighbouring oxygen atoms, see Fig. S2) and the environment of the Mg cation (the plane determined by the Mg cation and two neighbouring oxygen anions, see Fig. S3). In each case, the results of the in-house experimental measurements (Mo_exp, Ag_exp) are compared with synchrotron experiment (APS_exp) as well as with theoretical calculations (theor_0).

Let us first focus on the maps of the SO_2_ fragment. We see that on the maps of total electron density (contour 0.1e), there are no significant differences. However, we can say that in the case of theoretical calculations contours appear to be smoother than those obtained for the experimental results, which is also true for the laplacian and for the deformation density maps. Looking at the deformation density maps (red – minus values, blue – plus values, contours 0.05e) when the spherical IAM model of electron density is removed from the total EDD, we expect that, due to its rather covalent character, we will observe some density on the bond paths between S and O. In fact, for the theoretical results, we see some maxima on these paths as well as additional maxima (two per oxygen anion), which can be interpreted as free electron pairs of oxygens. Near the sulfur cation, we can see depletion of electron density. Generally, similar features that are a bit more perturbed can also be observed from the experimental results. Considering maps of the laplacian (blue contour – positive value, red contour – negative value), a positive maximum near the halfway point of each S—O bond path could be found.

For the maps of the MgO_2_ fragment (defined by the Mg cation and two O anions), we can observe that for the theoretical results two other oxygen atoms also reside almost in this plane (concentration of electron density above and below the Mg cation) whereas for the experimental data these oxygens were no longer visible in this plane. It is so because in the theoretically optimized structures, one observes an almost flat MgO_4_ moiety, but for structures refined against the experimental X-ray diffraction data, positions of other oxygens are off this plane. That is why we will discuss only similarities and differences observed for the Mg(4)⋯O(6) and Mg(4)⋯O(7) bond paths which are visible on all maps. The Mg⋯O interactions are expected to have much more ionic character than the S—O interactions, and in fact, there are no significant electron density maxima close to the midpoint of the Mg⋯O bond paths (see deformation density maps). Also there are no peaks of the laplacian along these paths.

The maps presented in Figs. S4 and S5 are difference maps. They were created by subtracting maps from Figs. S2 and S3 and present only the differences between pairs of subtracted maps.

**Figure 6:**
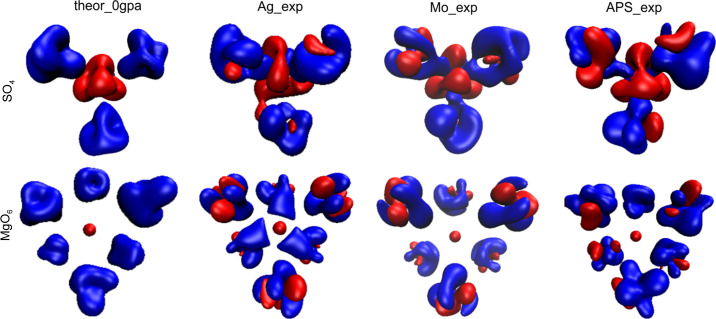
3D deformation density maps (red – minus values, blue – plus values, contour ±0.022 e).

As 2D maps are only cuts through 3D features, the 3D maps are, in our opinion, far more informative. In Fig. 6 ▸, deformation density maps for SO_4_ and MgO_6_ groups are presented. This gives a better view of details of rather complex distributions of density around ions and cations.

Although numerical data showing changes of electron distribution are quite informative, visualizations would make interpretation easier.

#### 3.2.3. Atomic/ionic basins

**Figure 7:**
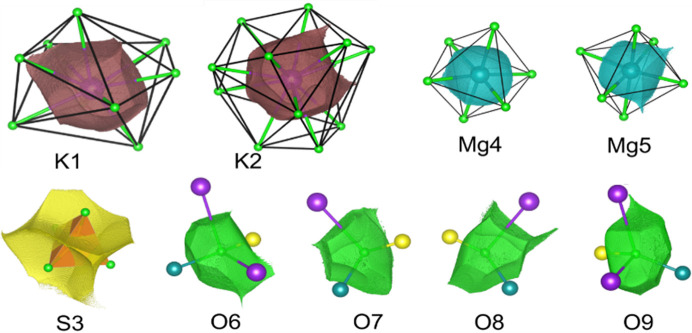
Relation between the polyhedral representation of ions in the crystal structure of Lb and atomic/ionic basins of particular ions.

There is no doubt that the polyhedra commonly used in mineralogy and crystallography at the structural level of X-ray results are not useful representations of the actual electron density as they neither have full representation of the central ion nor any of the corner ions (see Fig. 7 ▸).

**Figure 8:**
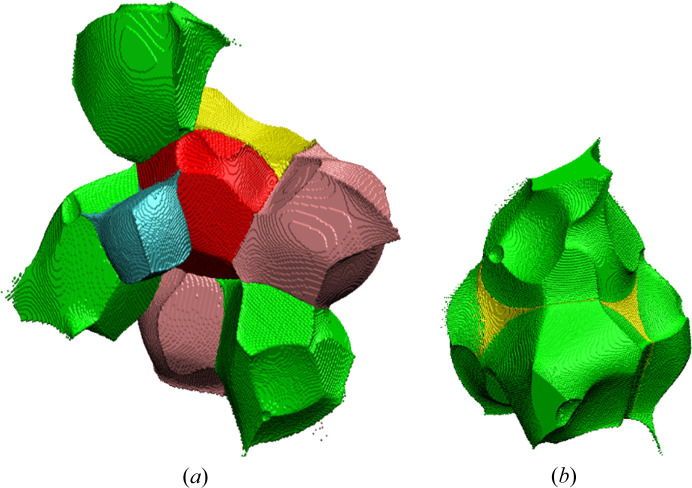
(*a*) Atomic basin of O9 (red shape) surrounded by basins belongs to sulfur (yellow), magnesium (cyan), oxygen (green) and potassium atoms (pink); (*b*) SO_4_ group (green – oxygen, yellow – sulfur).

So from time to time an old question returns (Brown, 2017 ▸): how big are atoms/ions in crystals (particularly in minerals)? This question is asked because atoms in crystals, particularly the partition of electron density, can be defined in many different ways. The quantum theory of atoms in molecules (QTAIM) (Bader, 1994 ▸) describes a far more useful method to partition electron density into atomic basins. This is based on the gradient lines of electron density which start from the local maxima of electron density (atoms/ions) and go to infinity with exceptions for those which go to the nearest atoms. Such gradient lines of electron density are called bond/interaction paths. The intersection of each bond path with its interatomic surface of zero flux of the gradient of electron density is a charge density minimum along the bond path and is known as the BCP. Obviously, particular atoms in the structure (in 3D space) can be surrounded by many neighbours (Fig. 8 ▸), so there are many bond/bonding paths oriented in different directions and having different length to their BCPs. That is why atomic basins have an irregular shape but completely fill the space (Fig. 8 ▸). We have illustrated this in Fig. 8 ▸ and corresponding movie M1 presented in the supporting information. It helps to understand how complex the arrangement of atomic basins is and how their shapes are affected by closest neighbours.

**Figure 9:**
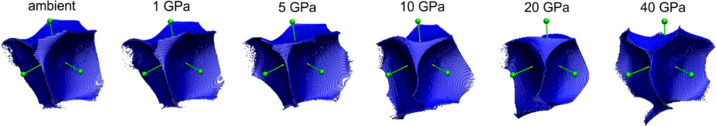
Sulfur atomic basin and the evolution of its shape as a function of pressure.

Atomic basins are topologically equivalent to polyhedra, in such a way that they also have faces, edges and vertices, and they satisfy Euler’s relationship (faces − edges + vertexes = 2). However, because of their irregular shapes, the basins have curved edges and faces. We can say that in Lb, the Mg basins are most similar to cubes with six faces [see Fig. 8 ▸(*a*), cyan shape] and the S basins to tetrahedra with four faces (see Fig. 9 ▸). Other basins such as K and especially O are much more irregular.

It could be beneficial to associate changes of EDD with the shapes of atomic basins. We should not treat atoms/ions or cations as points within the space of a particular unit cell. Atomic charge density is not a well defined property. It depends on a given definition. We use the Bader’s integrated atomic charges in this work. So electron density which belongs to particular atoms, fills some shapes (atomic basins), and these shapes, like bricks, form the crystal structure [see Figs. 8 ▸(*a*) and 8 ▸(*b*)]. That is why any changes of EDD caused by pressure will be visible as changes of the shapes of these bricks (atomic basins). Additionally, as a result of pressure, EDD within particular atomic basins will be changed. So let us look into this issue and see how atomic basins are changing as a function of pressure. There are nine different ions in the asymmetric part of Lb structure: four oxygen anions, two potassium cations, two magnesium cations and one sulfur cation. This means that we have nine different atomic basins to consider (see Fig. 7 ▸).

The sulfur atomic basin is one of the smallest and is characteristically defined by the atomic basins of the neighbouring oxygens. In fact, the sulfur basin is almost completely surrounded from each side by oxygen basins [see Fig. 8 ▸(*b*), movie M2]. Each of these surrounding oxygen basins perturbs the shape of the sulfur basin (see Fig. 9 ▸). Because the effective atomic basin describing the sulfur cation no longer has anything in common with its tetrahedron schematic. The issue is much more visible in the movie M3. It shows that in fact the tetrahedron and atomic basins overlap only in a small central region. On one hand a significant part of the basins goes beyond the polyhedra boundaries, on the other, the vertices of polyhedra are beyond the atomic basin.

The higher the pressure, the more significant the deformation of the sulfur atomic basin is. As a result of the squeezing caused by high pressure, the volumes of the atomic basins decrease. But they depict a delicate balance in the interactions of the crystal structure of Lb which is dependent on pressure. Their shapes will also be dependent on pressure. Of course SO_4_ is a very well defined anion so the shape of its basin is quite well conserved as a function of pressure.

Of course the higher the pressure, the smaller the volume of the unit cell. But when we investigate the data in Table 3 ▸ further, we can see that different basins do not change their volume proportionally to the decrease of the whole unit cell. For example, at less than 5 GPa, the volume of the whole unit cell is smaller by about 6.4% in relation to that at ambient pressure. However, one of the magnesium basins has a slightly expanded volume, but the potassium basins have shrunk by about 8.5–9.8% and one of the oxygen basins by 10.3%. This situation should be reflected somehow by changes of net atomic charges of those basins (Table 3 ▸). In fact the net atomic charge under 5 GPa appears to correspond quite well with the results from 1 GPa and ambient pressure. However, for 10 and 20 GPa, a significant discrepancy between charge of anions and cations is observed. As a rule, the whole unit cell should be neutral. Some small deviations *ca* ±1 electron (which is *ca* 0.1%) could be accepted. The shapes of atomic basins of all moieties present in the Lb structure under different pressure values are visualized in Figs S6–S14.

Moreover, to better display how neighbouring ions affect the shape of particular central ions, movies M4–M9 are presented.

#### 3.2.4. Changes of atomic/ionic basins as a function of pressure

**Figure 10:**
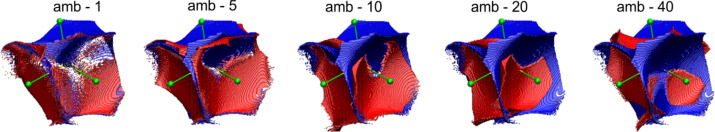
Differences between shape of the sulfur atomic basin (in blue) at ambient pressure and pressures ranging from 1 to 40 GPa (in red).

Even subtle changes of particular atomic basins are more visible when we subtract and superimpose the two shapes of the basins we want to compare. As observed from Fig. 10 ▸, at 1 GPa the shape of sulfur atomic basin is almost identical to that at ambient pressure. However, the higher the pressure the more deformed the atomic basin is, with respect to the corresponding atomic/ionic basin at ambient pressure. Such illustrations nicely show the anisotropy of interionic interactions under pressure. Atomic basins of all the other cations and anions and their changes as a function of pressure are presented in the supporting information.

**Figure 11:**
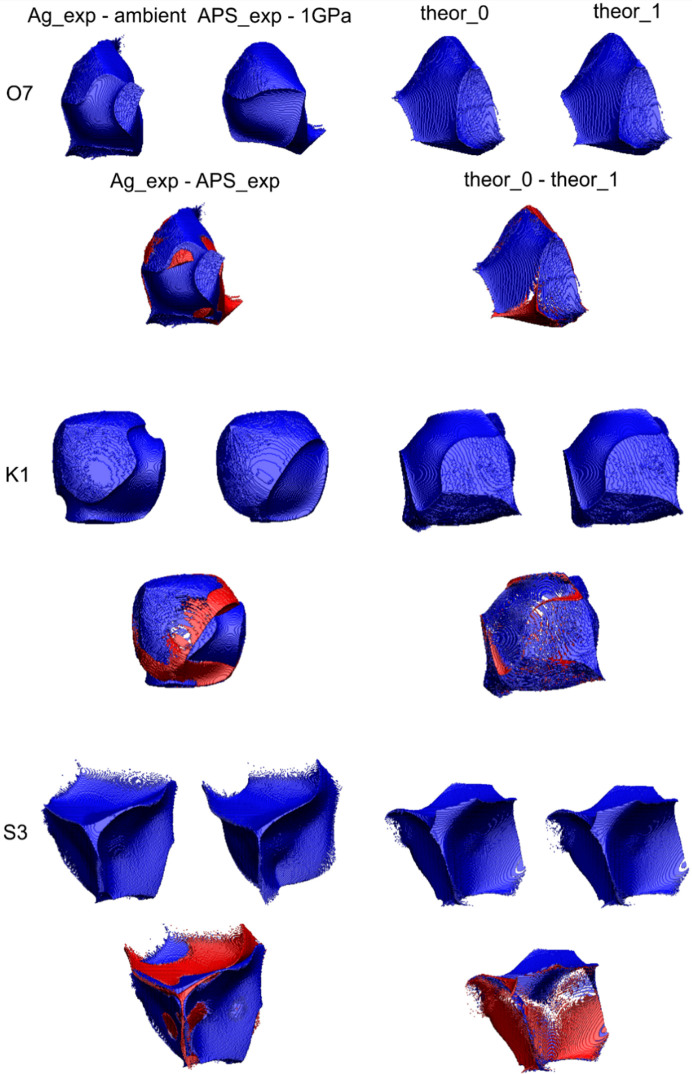
Comparison of selected atomic basins: O(7), K(1) and S(3) and their difference maps for experimental and theoretical data.

The sulfur atomic basins depicted in Figs. 9 ▸ and 10 ▸ as well as data presented in Table 3 ▸ correspond with results obtained on the basis of theoretical optimization of the Lb structure in *CRYSTAL17*. The comparison of selected examples of atomic basins obtained on the basis of experimental data and theoretical calculations is presented in Fig. 11 ▸.

When a particular atomic basin is squeezed under pressure, its volume shrinks and, as a result, the charge density within it must be redistributed. We will illustrate such processes by using an example of one of the oxygen anions/atoms. The atomic basin of O9 [red shape on Fig. 8 ▸(*a*)] is surrounded by the other ions: sulfur, magnesium and potassium cations and other oxygen anions. When the pressure is raised, and the atomic basin changes shape, some charge density inside it must be redistributed.

#### 3.2.5. Redistribution of charge density inside atomic/ionic basins

**Figure 12:**
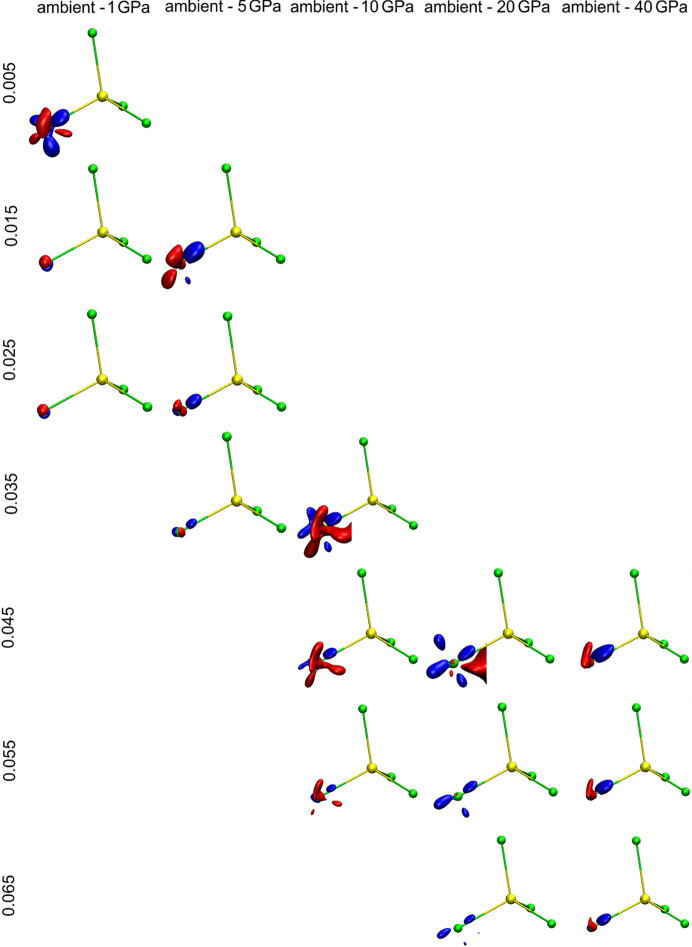
Differences in EDD at the O(9) anion in a range of pressure values from ambient to 40 GPa described by a set of isovalues. The regions in space which carry more total electron density (defined by the isosurface value) at ambient pressure than under particular pressure values are in blue and the regions for which there are more total electron density (as defined by the isocontour value) under pressure than at ambient pressure are in red. So we can interpret such figures as an illustration of the redistribution of charge (at a given contour isosurface value) from the blue regions at ambient pressure to the red regions under higher pressure values.

When external stimuli such as pressure or temperature are applied, charge also redistributes inside atomic/ionic basins. The way in which the EDD changes with pressure is visible when difference maps comparing two pressures are created (Fig. 12 ▸). They are presented as a set of difference electron density maps showing changes at the O(9) anion/atom. In each case, a map results from subtracting the total charge density at the O(9) anion at ambient pressure from the high-pressure data. Maps for five different pressure ranges are depicted. Moreover, to show how EDD is sensitive to pressure changes, for each pressure value, sets of different isosurfaces are used. This is necessary because for maps showing differences within the low-pressure range (as ambient and 1 or 5 GPa) changes are quite subtle and small isovalues are needed to present those changes. Whereas for maps showing differences within the large pressure range (between ambient and 20 or 40 GPa) changes are significant and to show them some larger isovalues are needed. Additionally, in the movies provided in the supporting information, movie M13 is presented differences in EDD at O(9) in a pressure range between ambient and 1 GPa.

**Figure 13:**
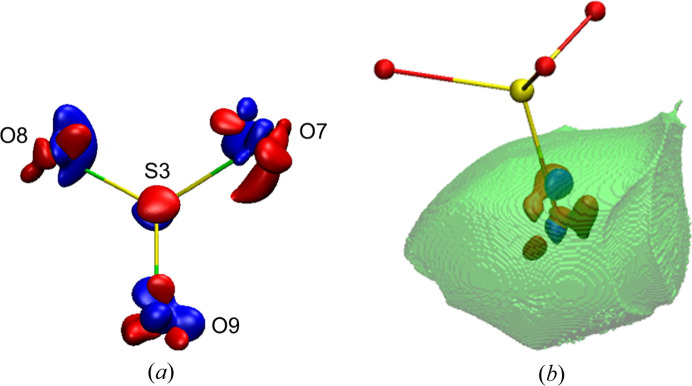
Difference of total electron density at the (*a*) SO_4_ group, difference between ambient pressure and 1 GPa (iso-contour: + 0.005 blue and −0.005 red). (*b*) Map of the difference between ambient pressure and 10 GPa (iso-contour: +0.05 and −0.05; blue and red, respectively).

Of course such changes in EDD caused by pressure within a particular atomic basin do not occur in isolation from neighbouring basins. This is a concerted mechanism. That is why we should also take into account the changes in atomic basins of the surrounding atoms/ions. Fig. 13 ▸(*a*) shows the SO_4_ group and Fig. 13 ▸(*b*) the O(9) atomic basin as well as changes of EDD within this basin. Such maps where two total electron densities under different pressures are subtracted from each other, two factors are responsible for the visualized changes of EDD. One component is the true difference of electron density at the position of the considered particular atom/ion and in its vicinity. This is the main cause of differences at the centre of such grids [at the O(9) anion position]. The second factor, whose participation increases when we are further away from the centre of the grid, is the change of atomic position in space. The higher the difference of pressure values, the more significant changes in atomic positions. Although at the centre of map the situation is normalized in a way that the atom considered is always exactly at the centre, the closer to the edges of map the higher discrepancies in the atomic positions become.

## 4. Conclusions

Our investigations of the Lb structure under pressure leads to several conclusions. Firstly, when we take into consideration just structural and thermal parameters of Lb we can distinguish ‘sensitive’ and ‘resistant’ ions and interionic interactions. Sulfur and magnesium cations belong to the group of resistant ions. Their ADPs and atomic basins are the most impervious to pressure, the similarity index for ADPs within the investigated pressure range is also quite small for these ions and the S—O and Mg—O interionic contacts undergo a relatively small reduction. At the other extreme potassium cations and oxygen anions are found. Their ADPs and atomic basins are compressed significantly under pressure. As a result, shortening of K—O contacts is more pronounced. This information suggests where, in the structure of Lb, we should expect some visible changes of EDD under pressure around potassium and oxygen ions.

Another important conclusion from our studies is the importance of atomic basins which seem to be better defined and more useful than typical polyhedra used in inorganic chemistry and mineralogy. Figures showing atomic basins, side by side, show not only when the pressure is significant enough to cause deformation of electron density but also how 3D space belonging to a particular ion is changing. Moreover the changing shape of integrated atomic basins provides information about the response of electron density to the external stimuli such as pressure or temperature. One can also trace what is happening inside such an atomic electron density basin. The effects of the electron density redistribution within the atomic basins can be visualized in the form of 2D and, particularly, 3D differential maps comparing particular ions under two selected values of pressure. In the case of Lb, the most noticeable changes in EDD are present at sensitive oxygen anions.

The next question to answer is whether or not any incompleteness in our experimental data can affect the results. On the basis of our studies such as the gradual reduction of completeness for experimental data, we can conclude that full completeness is not required in experimental quantitative charge density studies. Less than 100% completeness of the starting *hkl* data can still produce very good results for the final charge density. In the case of Lb this threshold was about 80–90%. This means that we do not observe any serious deviations in the values of structural, electronic or thermal parameters up to this limit of completeness. For details, see the supporting information.

We also want to stress the importance of theoretical high-pressure studies which allow us to significantly extend the range of pressures we can apply to study different phenomena. We did our best to perform our electron density refinements in exactly the same way for both experimental and theoretical data. Of course each dataset, experimental or theoretical, possesses its own specific errors due to the way it was obtained or collected. Admittedly, the output of refinements based on experimental *hkl* files is actually very similar to the results obtained on the basis of theoretically calculated dynamic structure factors, a fact which cross-validates both approaches.

The ICSD deposition numbers 2093991–2093996 contain the supplementary crystallographic data for the investigated system. This data can be obtained freely via http://www.ccdc.cam.ac.uk/data_request/cif, or by contacting data_request@ccdc.cam.ac.uk or the Cambridge Crystallographic Data Centre directly.

## 5. Related literature

The following references are cited in the supporting information: Becke & Edgecombe (1990 ▸); Clementi & Roetti (1974 ▸); Dominiak *et al.* (2006 ▸); Hirshfeld (1977 ▸); Johnson *et al.* (2010 ▸); Kirzhnits (1957 ▸); Koch & Popelier (1995 ▸); Macchi *et al.* (1998*a* ▸,*b* ▸); Silvi & Savin (1994 ▸); Tsirelson & Stash (2002 ▸); Tsirelson (2002 ▸); Volkov *et al.* (2001 ▸); Zuckschwerdt (1891 ▸).

## Supplementary Material

Crystal structure: contains datablock(s) Langbeinite_silver_source_structural, Langbeinite_silver_source_multipole, Langbeinite_molybdenum_source_structural, Langbeinite_molybdenum_source_multipole, Langbeinite_synchrotron_source_structural, Langbeinite_synchrotron_source_multipole. DOI: 10.1107/S2052252521012628/ti5023sup1.cif

Structure factors: contains datablock(s) Langbeinite_silver_source. DOI: 10.1107/S2052252521012628/ti5023sup2.fcf

Structure factors: contains datablock(s) Langbeinite_molybdenum_source. DOI: 10.1107/S2052252521012628/ti5023sup3.fcf

Structure factors: contains datablock(s) Langbeinite_synchrotron_source. DOI: 10.1107/S2052252521012628/ti5023sup4.fcf

Animations M1-M13. DOI: 10.1107/S2052252521012628/ti5023sup5.pptx

Movie M1. DOI: 10.1107/S2052252521012628/ti5023sup6.avi

Movie M2. DOI: 10.1107/S2052252521012628/ti5023sup7.avi

Movie M3. DOI: 10.1107/S2052252521012628/ti5023sup8.avi

Movie M4. DOI: 10.1107/S2052252521012628/ti5023sup9.avi

Movie M5. DOI: 10.1107/S2052252521012628/ti5023sup10.avi

Movie M6. DOI: 10.1107/S2052252521012628/ti5023sup11.avi

Movie M7. DOI: 10.1107/S2052252521012628/ti5023sup12.avi

Movie M8. DOI: 10.1107/S2052252521012628/ti5023sup13.avi

Movie M9. DOI: 10.1107/S2052252521012628/ti5023sup14.avi

Movie M10. DOI: 10.1107/S2052252521012628/ti5023sup15.avi

Movie M11. DOI: 10.1107/S2052252521012628/ti5023sup16.avi

Movie M12. DOI: 10.1107/S2052252521012628/ti5023sup17.avi

Movie M13. DOI: 10.1107/S2052252521012628/ti5023sup18.avi

Supporting figures and tables. DOI: 10.1107/S2052252521012628/ti5023sup19.pdf

CCDC references: 2093991, 2093992, 2093993, 2093994, 2093995, 2093996

## Figures and Tables

**Table 1:** Selected crystal data for spherical and multipole refinements of langbeinite at ambient pressure and at 1 GPa Detailed tables of structural and experimental parameters, data completeness, and comparison of statistical characteristics of the datasets are provided in the supporting information.

Data source	Ag_exp	Mo_exp	APS_exp
Spherical refinement			
Pressure (GPa)	Ambient	Ambient	1
*a* (Å)	9.91895 (2)	9.91977 (3)	9.90450 (7)
*V* (Å^3^)	975.88 (1)	976.12 (1)	971.62 (2)
*Z*, *F*(000)	4, 824	4, 824	4, 824
*D_x_* (Mg m^−3^)	2.825	2.824	2.837
Wavelength (Å)	0.5609	0.7107	0.4340
μ (mm^−1^)	0.91	1.81	0.45
Crystal size (mm)	0.50 × 0.43 × 0.28	0.21 × 0.10 × 0.04	†
Absorption correction	Numerical absorption correction *T*_min_ = 0.217, *T*_max_ = 1.000	Numerical absorption correction *T*_min_ = 0.616, *T*_max_ = 1.000	Empirical multiscan *T*_min_ = 0.832, *T*_max_ = 1.000‡
Measured reflections	168589	63785	14881
Independent reflections	5360	5785	3008
Observed reflections	5292	5569	2712§
*R*_int_	0.031	0.031	0.045‡
θ values (°)	θ_min_ = 2.3, θ_max_ = 44.6	θ_min_ = 2.9, θ_max_ = 66.0	θ_min_ = 1.8, θ_max_ = 27.7
(sin θ/λ)_max_ (Å^−1^)	1.252	1.285	1.071
Completeness (%)	100	100	89§
Range of *h*, *k*, *l*	*h* = −24→24	*h* = −24→25	*h* = −21→11‡
	*k* = −24→24	*k* = −25→21	*k* = −8→13
	*l* = −24→24	*l* = −24→24	*l* = −11→15
Refinement on, parameters, reflections	*F* ^2^/58/5360	*F* ^2^/58/5785	*F* ^2^/58/3008
*R*[*F* ^2^ > 2σ(*F* ^2^)], *wR*(*F* ^2^), *S*	0.022, 0.060, 1.10	0.022, 0.059, 1.06	0.033, 0.076, 1.03
Weighting scheme	*w* = 1/[σ^2^(*F*_o_^2^) + (0.0284*P*)^2^ + 0.1847*P*] where *P* = (*F*_o_^2^ + 2*F*_c_^2^)/3	*w* = 1/[σ^2^(*F*_o_^2^) + (0.0272*P*)^2^ + 0.1257*P*] where *P* = (*F*_o_^2^ + 2*F*_c_^2^)/3	*w* = 1/[σ^2^(*F*_o_^2^) + (0.0267*P*)^2^] where *P* = (*F*_o_^2^ + 2*F*_c_^2^)/3
(Δ/σ)_max_	0.001	0.001	0.001
Δ〈_max_, Δ〉_min_ (eÅ^−3^)	0.91, −0.41	0.90, −0.54	0.73, −0.64
Multipole refinement			
Refinement on, parameters, reflections	*F* ^2^/ 223 / 5234	*F* ^2^/ 223 /5380	*F* ^2^/ 223 /2504
*R*[*F*2 > 2σ (*F*2)], *R*(all)	0.0219, 0.0220	0.0186, 0.0189	0.0452, 0.0477
*wR*[*F*2 > 2σ (*F*2)], *S*	0.0519, 1.6644	0.0477, 1.2381	0.0524, 0.966
Weighting scheme	*w* = 1/[σ^2^(*F*_o_^2^)]	*w* = 1/[σ^2^(*F*_o_^2^)]	*w* = 1/[σ^2^(*F*_o_^2^)]
(Δ/σ)_max_	0.00265	0.02314	0.00236
Δ〉_max_, Δ〉_min_ (eÅ^−3^)	0.533, −0.563	0.511, −0.568	0.628, −0.652

† Three crystals in DAC.

‡ Data for the first component.

§ Data for all three components after concatenation and merging.

**Table 2:** Net atomic charge Ω (e^−^) at atoms/ions and atomic/ionic volumes *V* (Å^3^)

	Ag_exp Ω, *V*	Ag_exp (APS completeness) Ω, *V*	Mo_exp Ω, *V*	APS_exp Ω *V*
K1	+1.16, 19.0	+1.15, 19.1	+1.21, 18.0	+1.18, 19.5
K2	+1.12, 20.0	+1.09, 19.9	+0.99, 19.8	+0.63, 19.9
S3	+3.06, 5.6	+3.13, 5.4	+3.13, 5.6	+3.64, 4.4
Mg4	+1.53, 5.3	+1.55, 5.1	+1.85, 4.2	+1.78, 4.5
Mg5	+1.55, 5.8	+1.56, 5.8	+1.91, 4.3	+1.71, 5.4
O6	−1.03, 13.2	−1.08, 13.4	−1.29, 14.2	−1.28, 14.9
O7	−1.09, 15.4	−1.10, 15.6	−1.35, 16.0	−1.03, 14.9
O8	−1.02, 13.8	−1.05, 14.0	−1.10, 14.8	−1.07, 13.9
O9	−1.72, 17.2	−1.7, 16.9	−1.46, 16.3	−2.05, 18.4
Total charge	−0.16	−0.20	−1.00	−0.28
Total volume	982.8	983.2	988.0	995.2

**Table 3:** Net atomic charges Ω (e^−^) and volumes *V* [Å^3^] of atomic basins

	Ambient Ω, *V*	1 GPa Ω, *V*	5 GPa Ω, *V*	10 GPa Ω, *V*	20 GPa Ω, *V*	40 GPa Ω, *V*
K1	+1.03, 21.2	+ 1.03, 20.5	+0.97, 19.4	+1.03, 17.7	+1.05, 16.3	+0.89, 14.5
K2	+1.00, 20.4	+1.01, 19.6	+1.07, 18.4	+1.03, 17.4	+0.93, 16.0	+1.3, 13.7
S3	+3.22, 6.1	+3.19, 6.1	+3.19, 5.9	+3.18, 6.0	+3.13, 5.8	+3.16, 5.0
Mg4	+1.91, 4.6	+1.91, 4.6	+1.84, 5.2	+1.84, 5.1	+1.74, 4.7	+1.93, 4.2
Mg5	+1.91, 4.6	+1.91, 5.2	+1.81, 5.0	+1.80, 4.9	+1.69, 4.6	+1.83, 4.3
O6	−1.27, 14.6	−1.23, 14.1	−1.16, 13.1	−1.11, 12.3	−1.15, 11.3	−1.44, 11.1
O7	−1.34, 14.8	−1.37, 14.9	−1.36, 14.2	−1.45, 14.0	−1.39, 12.6	−0.95, 9.9
O8	−1.44, 14.9	−1.42, 14.5	−1.37, 13.3	−1.35, 12.4	−1.23, 11.2	−1.56, 10.0
O9	−1.09, 14.3	−1.1, 14.2	−1.17, 13.9	−1.15, 13.3	−1.20, 12.2	−1.14, 10.7
Total charge	+0.36	+0.28	+0.32	+0.24	−0.44	+0.64
Total volume	979.6	965.2	916.8	876.4	803.6	707.2
