# Supplementary material for: Tracing electron density changes in langbeinite under pressure
Source: IUCrJ. 2021 Dec 23;9(Pt 1):146–62. doi: 10.1107/S2052252521012628 (PMC8733888; doi:10.1107/S2052252521012628)
Supplement: Supplementary file 5 [file m-09-00146-sup5.pptx]

## Slide 1
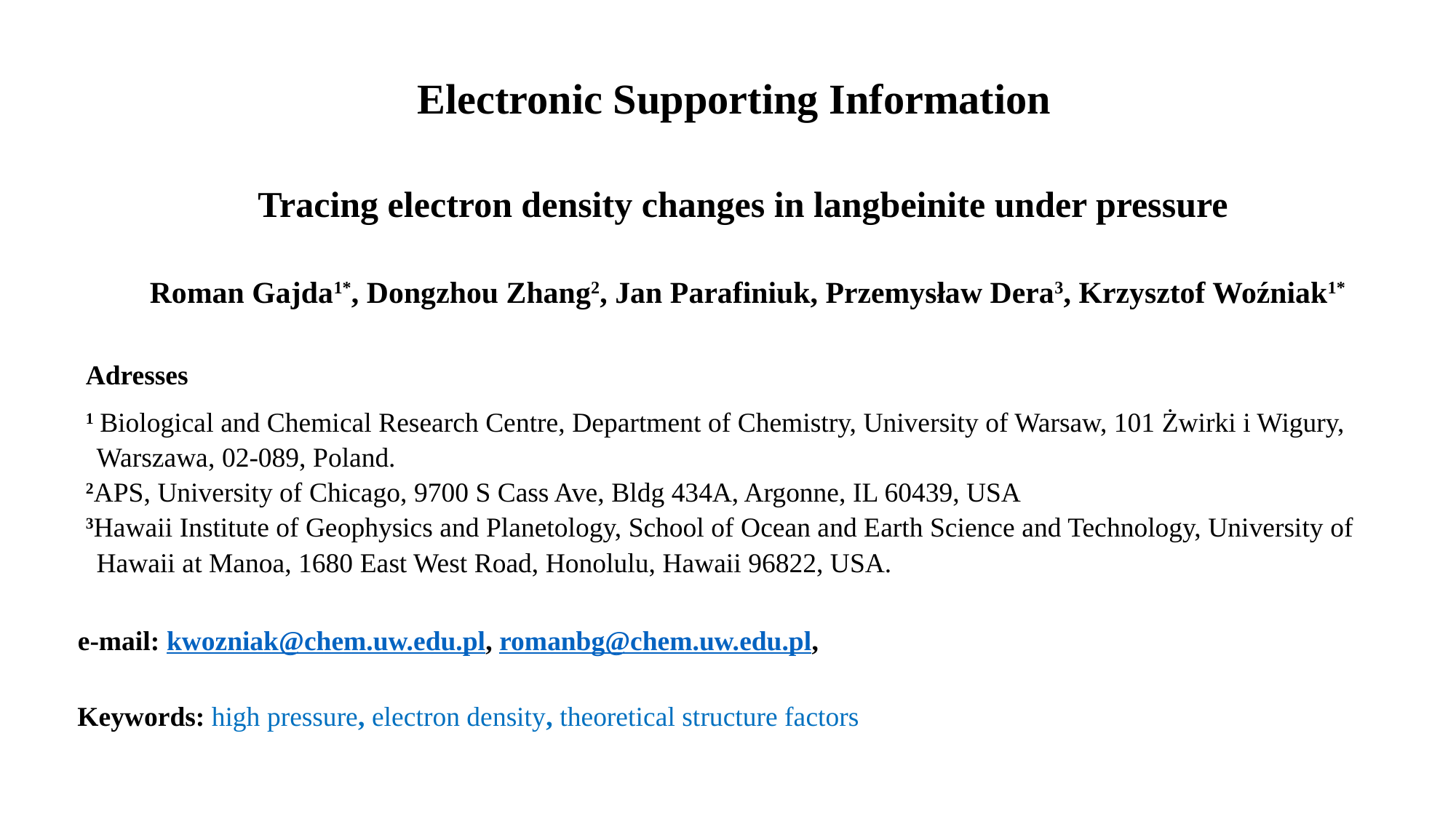

Electronic Supporting Information
Tracing electron density changes in langbeinite under pressure
Roman Gajda1*, Dongzhou Zhang2, Jan Parafiniuk, Przemysław Dera3, Krzysztof Woźniak1*
Adresses
1 Biological and Chemical Research Centre, Department of Chemistry, University of Warsaw, 101 Żwirki i Wigury, Warszawa, 02-089, Poland.
2APS, University of Chicago, 9700 S Cass Ave, Bldg 434A, Argonne, IL 60439, USA
3Hawaii Institute of Geophysics and Planetology, School of Ocean and Earth Science and Technology, University of Hawaii at Manoa, 1680 East West Road, Honolulu, Hawaii 96822, USA.
e-mail: kwozniak@chem.uw.edu.pl, romanbg@chem.uw.edu.pl,
Keywords: high pressure, electron density, theoretical structure factors

## Slide 2
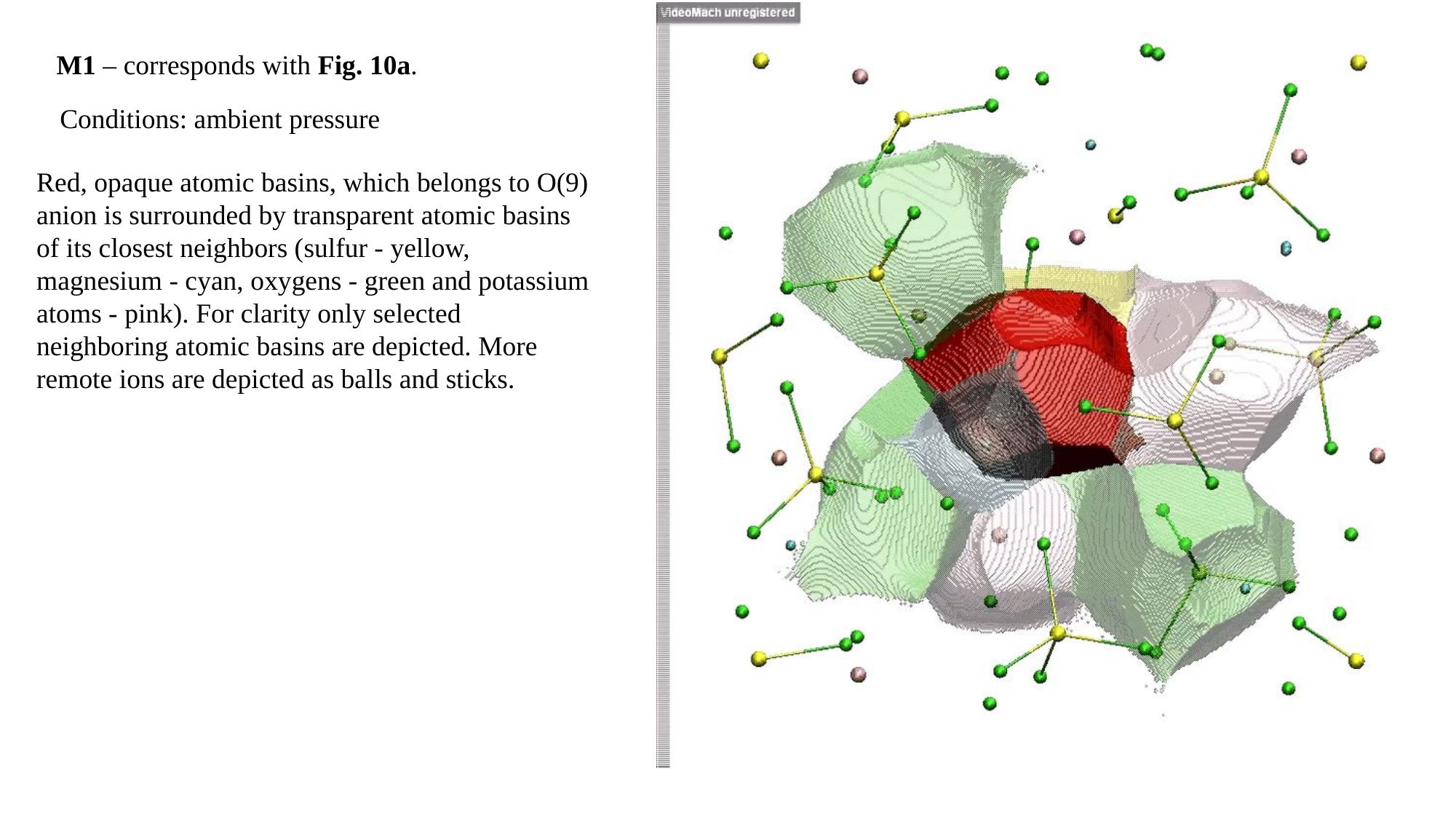

M1 – corresponds with Fig. 10a.
Conditions: ambient pressure
Red, opaque atomic basins, which belongs to O(9) anion is surrounded by transparent atomic basins of its closest neighbors (sulfur - yellow, magnesium - cyan, oxygens - green and potassium atoms - pink). For clarity only selected neighboring atomic basins are depicted. More remote ions are depicted as balls and sticks.

## Slide 3
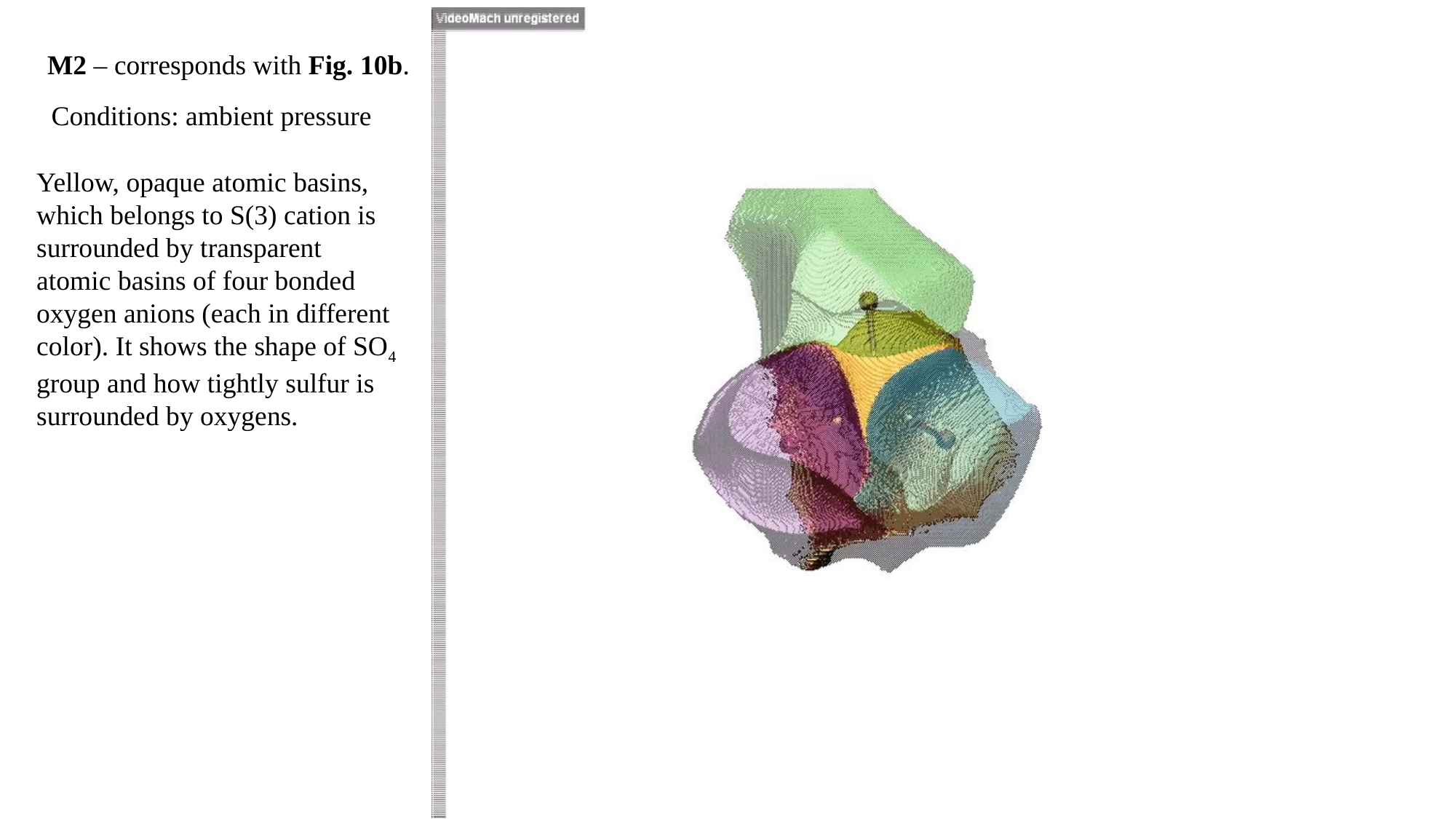

M2 – corresponds with Fig. 10b.
Conditions: ambient pressure
Yellow, opaque atomic basins, which belongs to S(3) cation is surrounded by transparent atomic basins of four bonded oxygen anions (each in different color). It shows the shape of SO4 group and how tightly sulfur is surrounded by oxygens.

## Slide 4
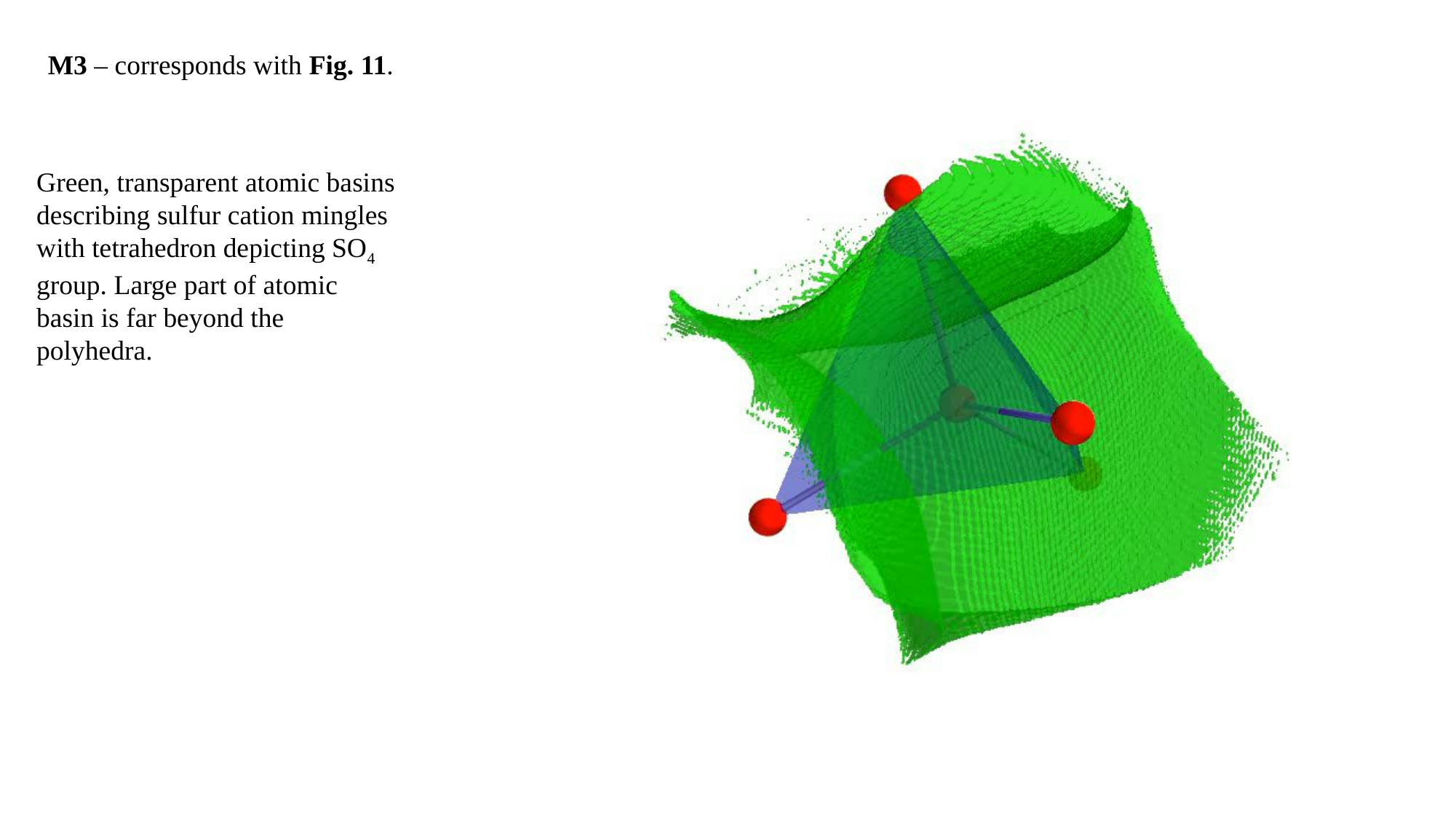

M3 – corresponds with Fig. 11.
Green, transparent atomic basins describing sulfur cation mingles with tetrahedron depicting SO4 group. Large part of atomic basin is far beyond the polyhedra.

## Slide 5
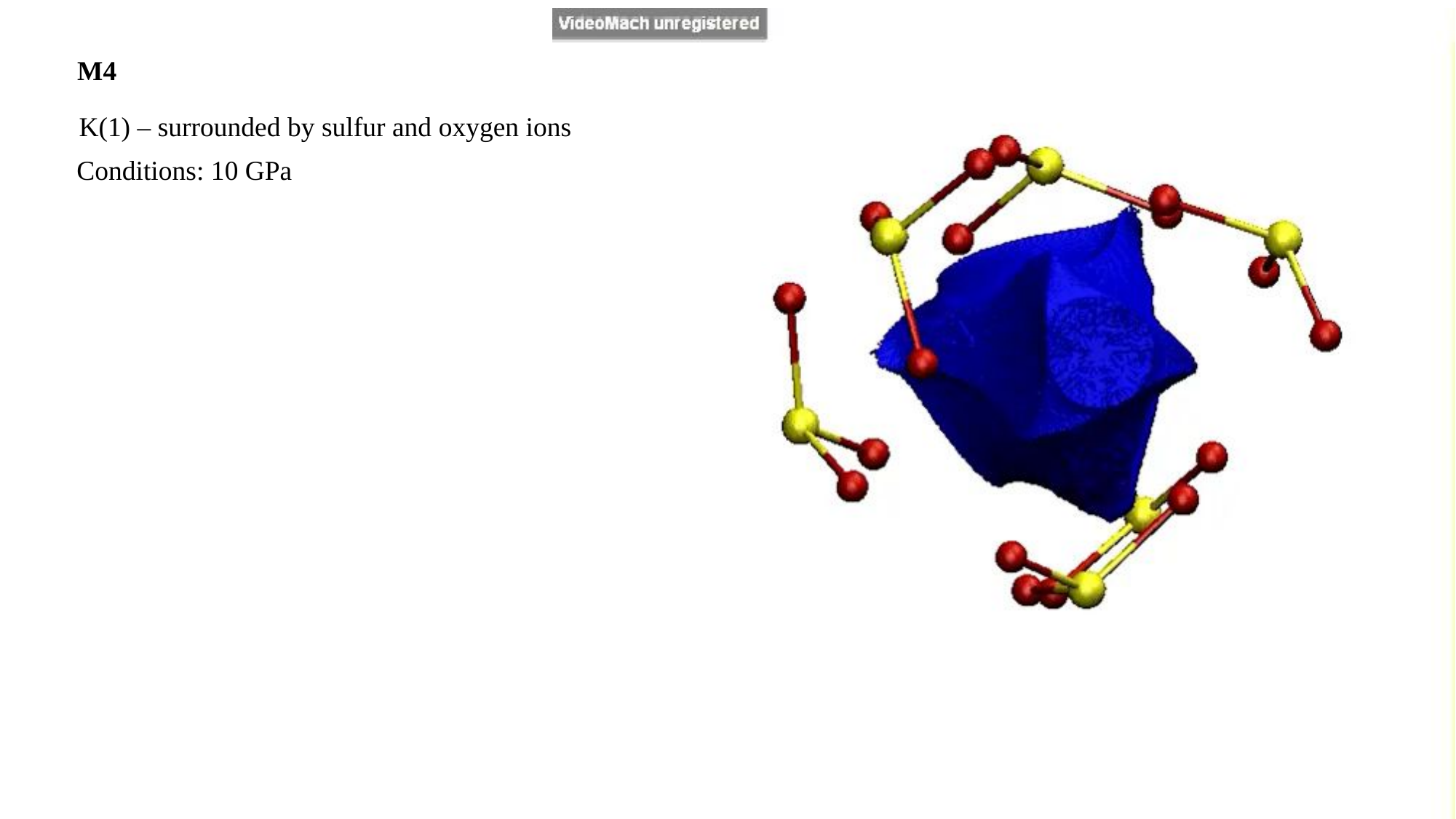

M4
K(1) – surrounded by sulfur and oxygen ions
Conditions: 10 GPa

## Slide 6
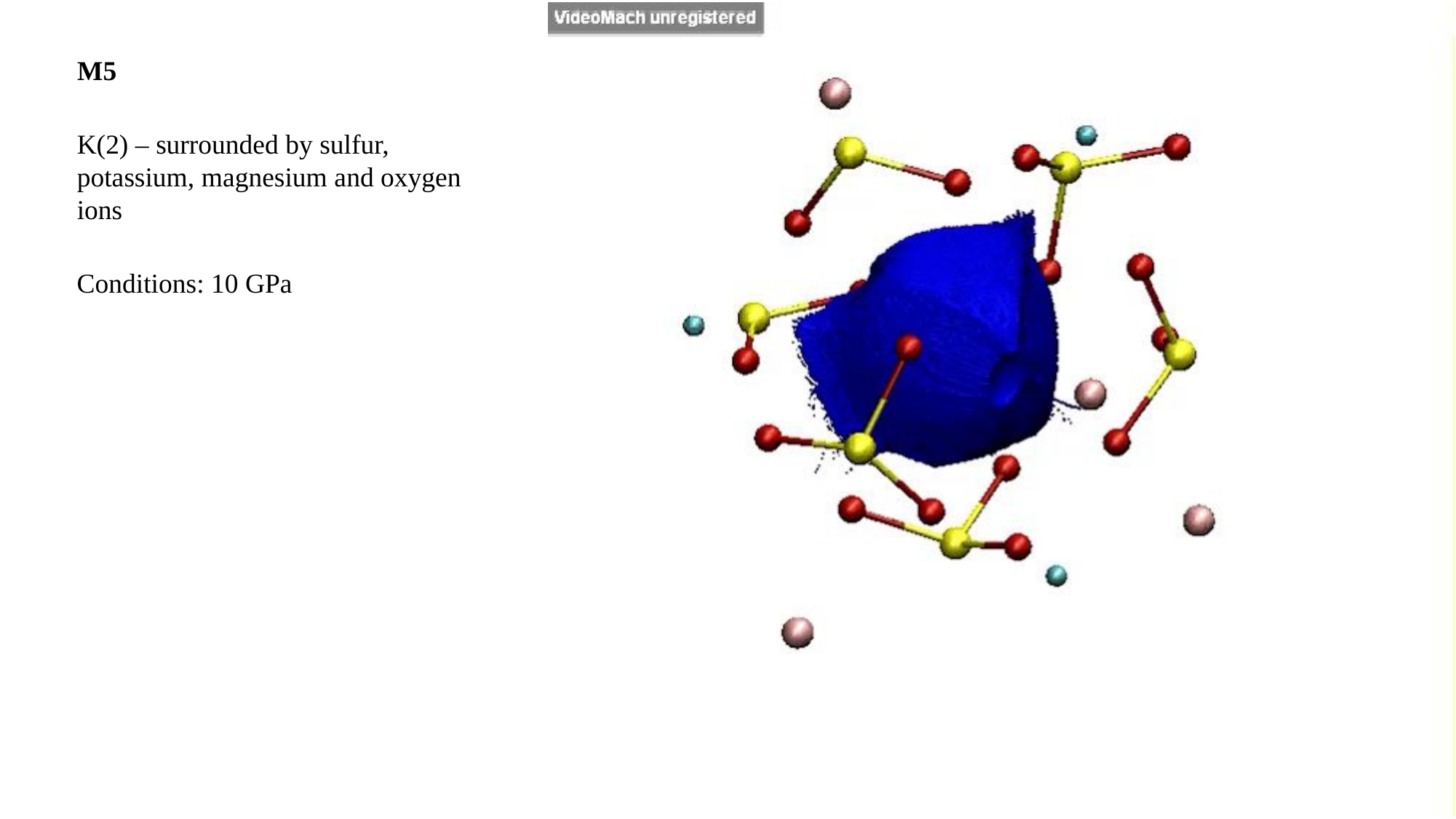

M5
K(2) – surrounded by sulfur, potassium, magnesium and oxygen ions
Conditions: 10 GPa

## Slide 7
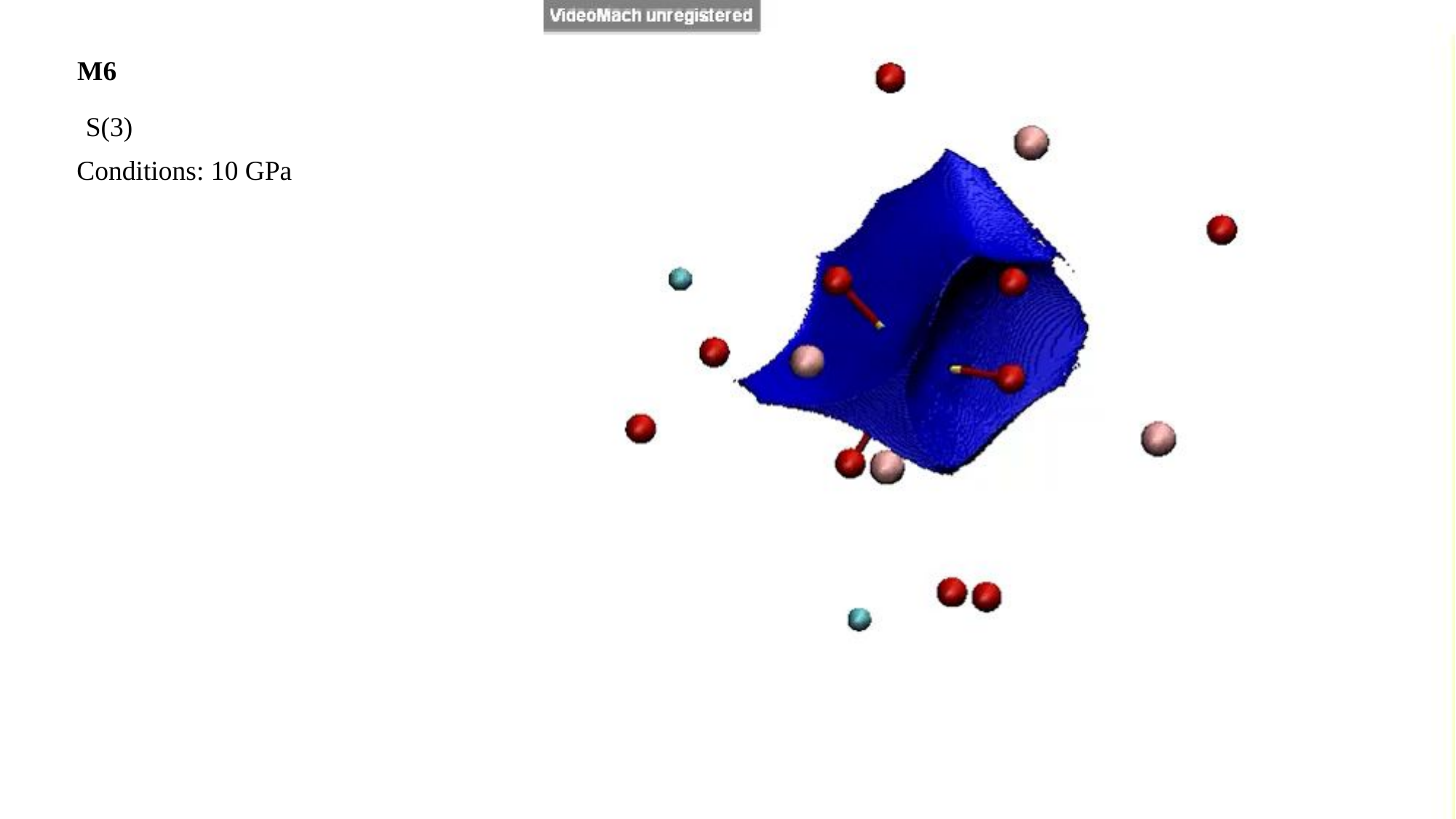

M6
S(3)
Conditions: 10 GPa

## Slide 8
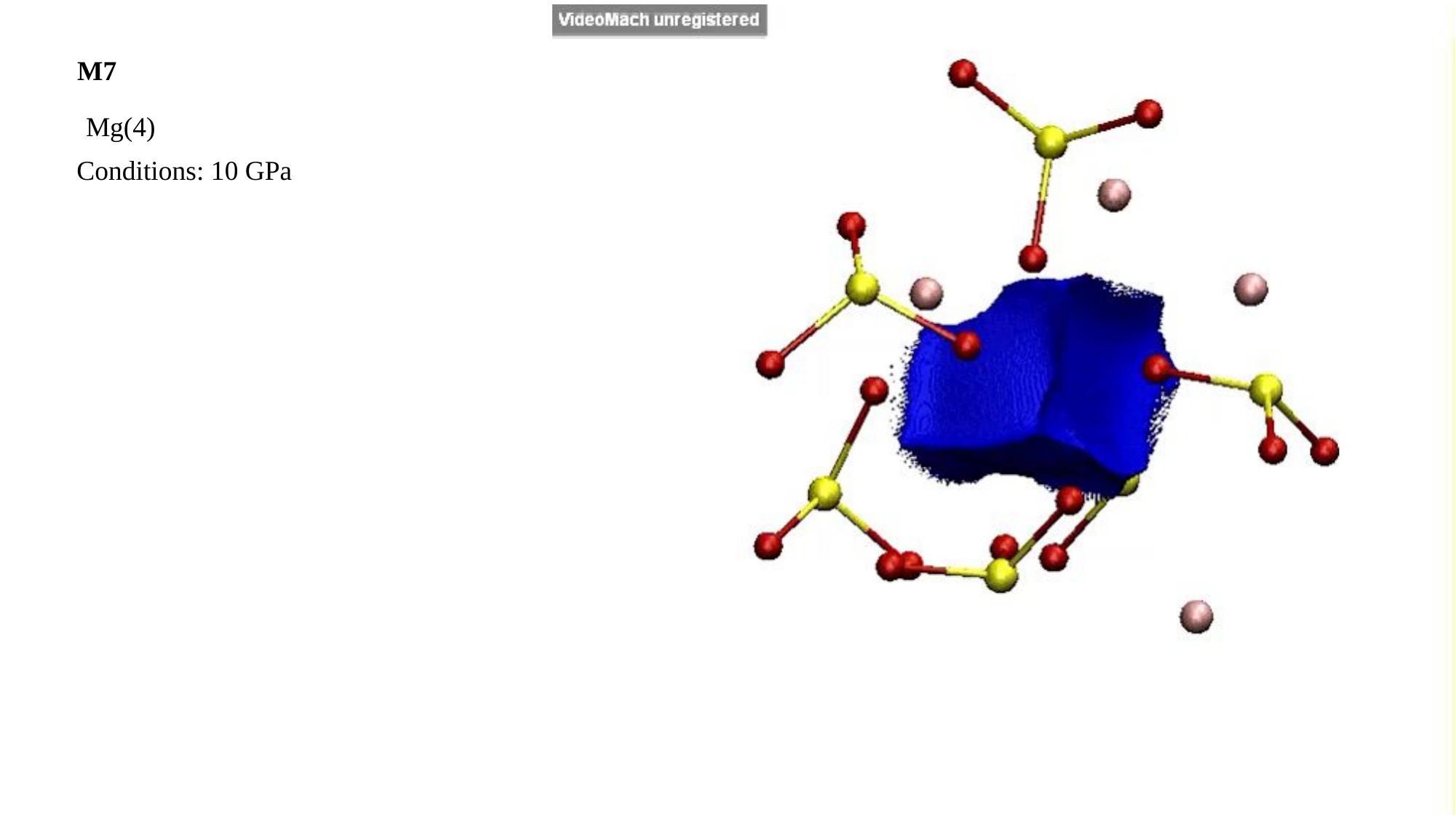

M7
Mg(4)
Conditions: 10 GPa

## Slide 9
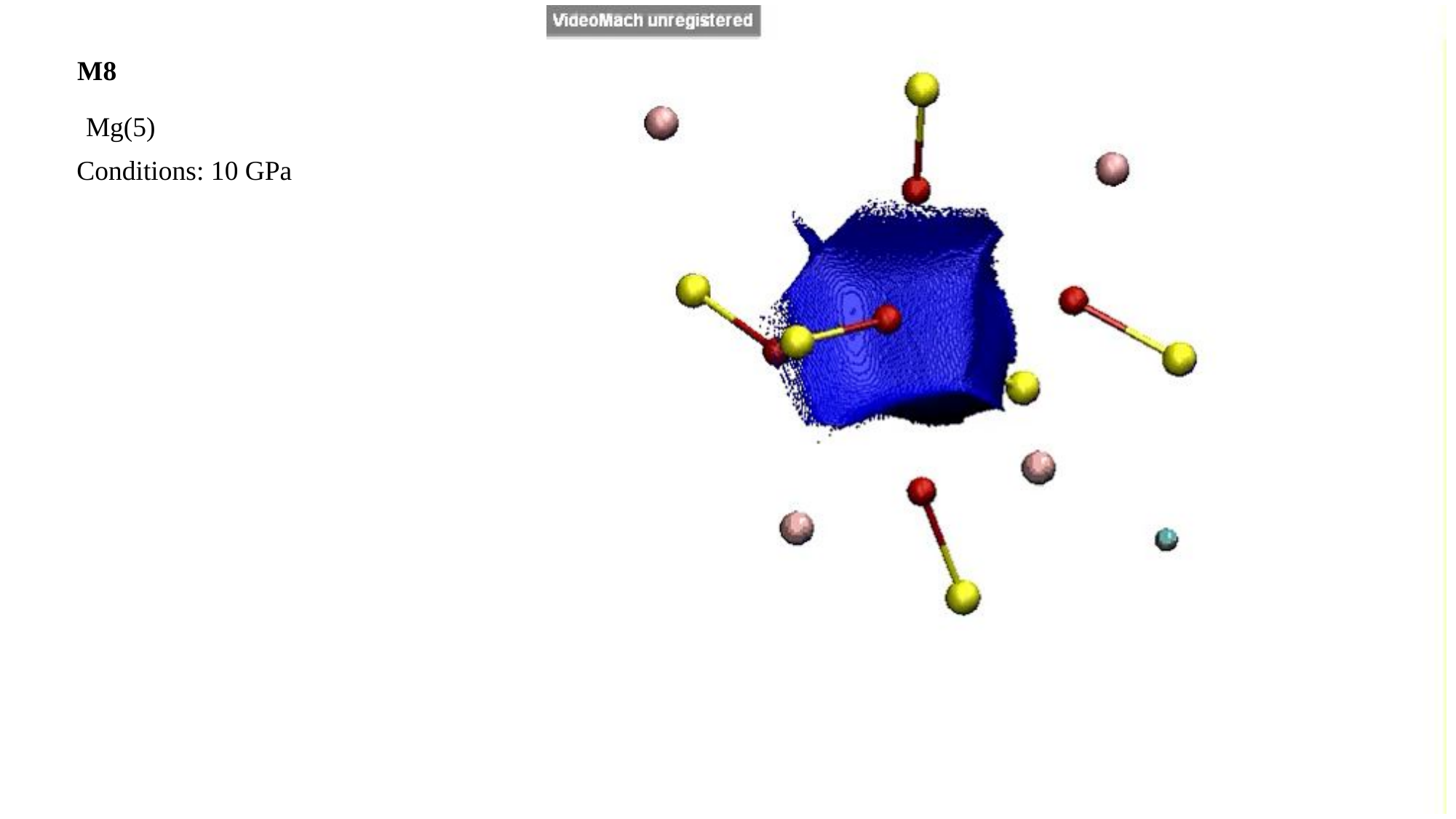

M8
Mg(5)
Conditions: 10 GPa

## Slide 10
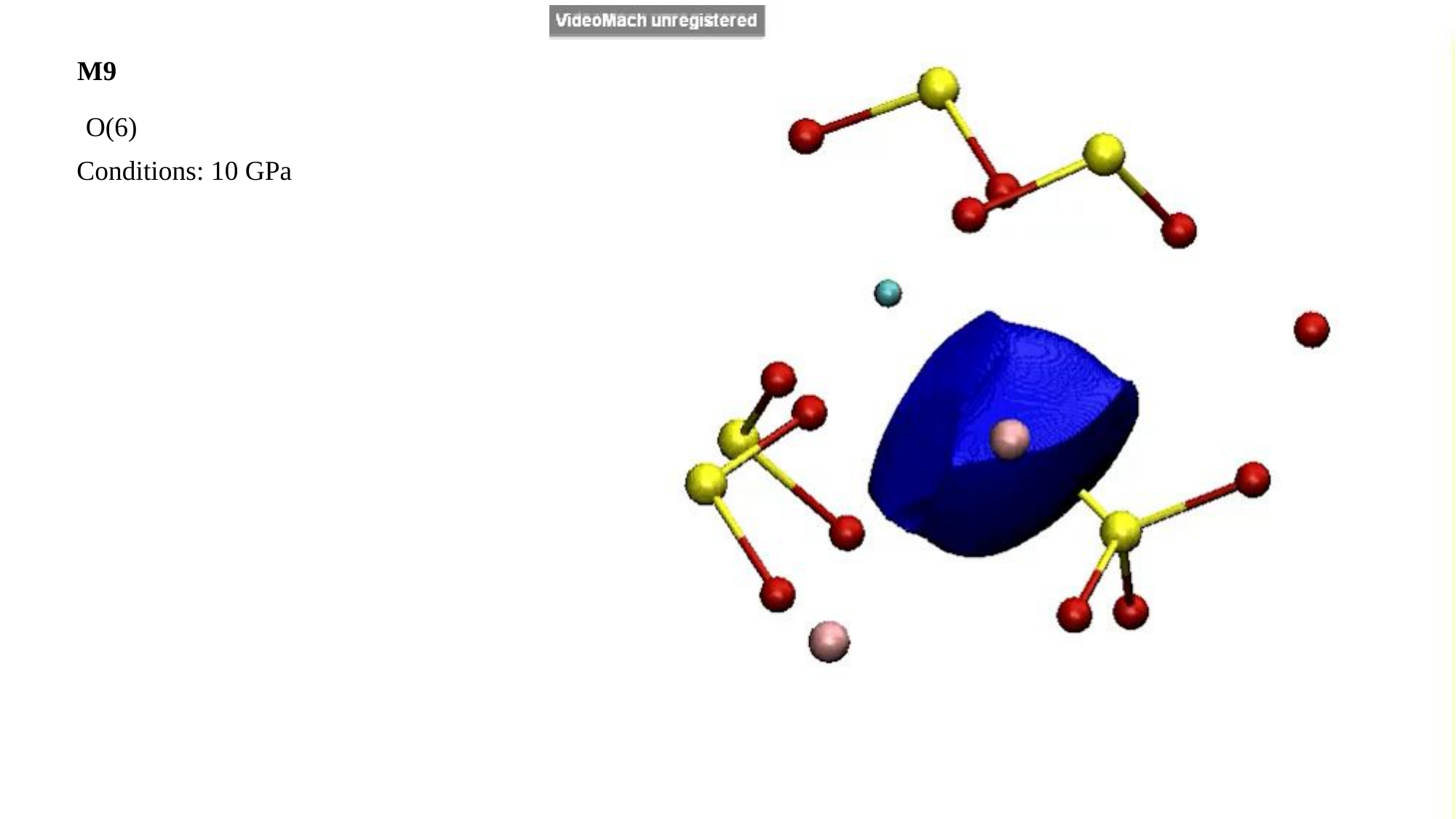

M9
O(6)
Conditions: 10 GPa

## Slide 11
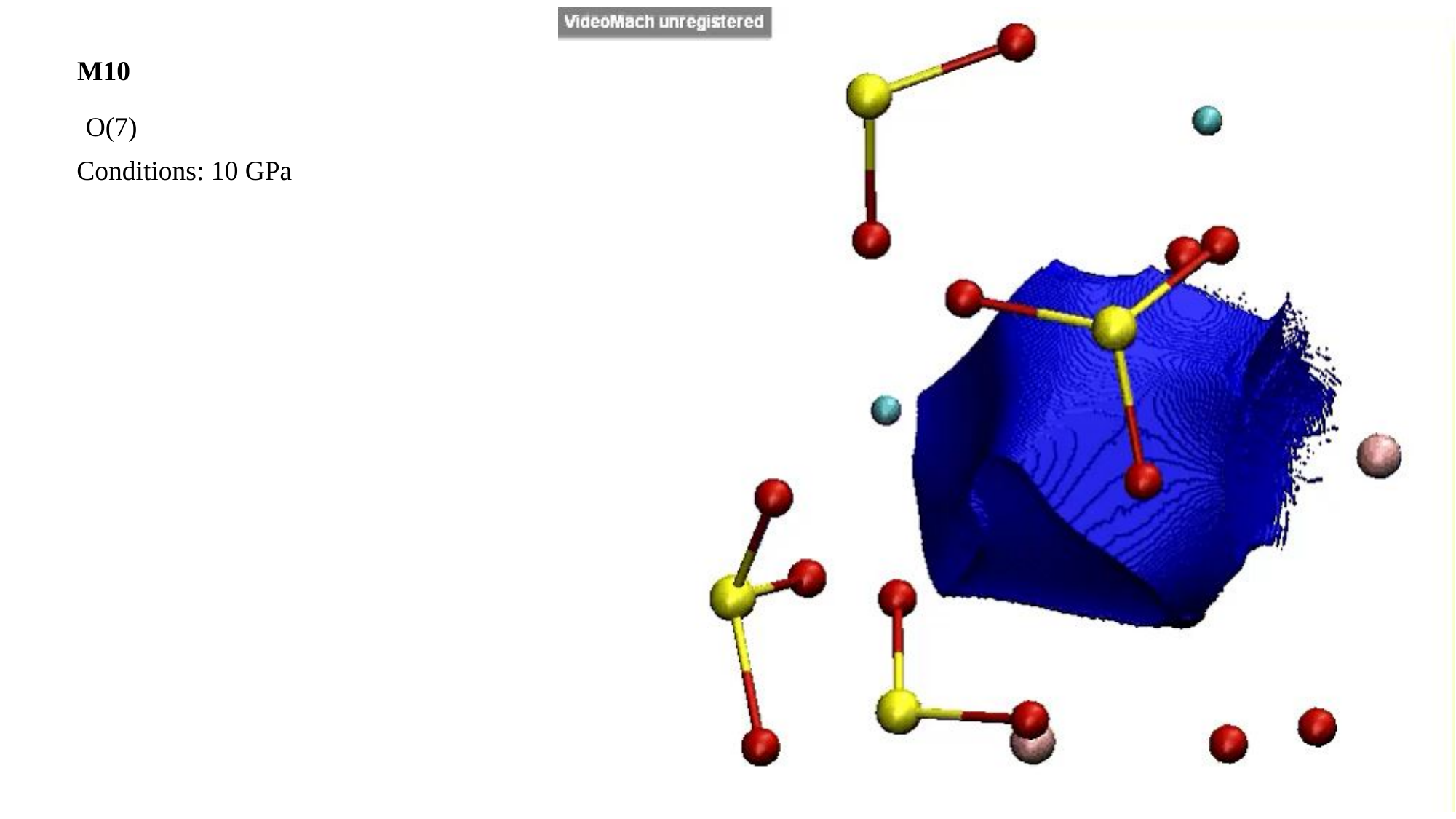

M10
O(7)
Conditions: 10 GPa

## Slide 12
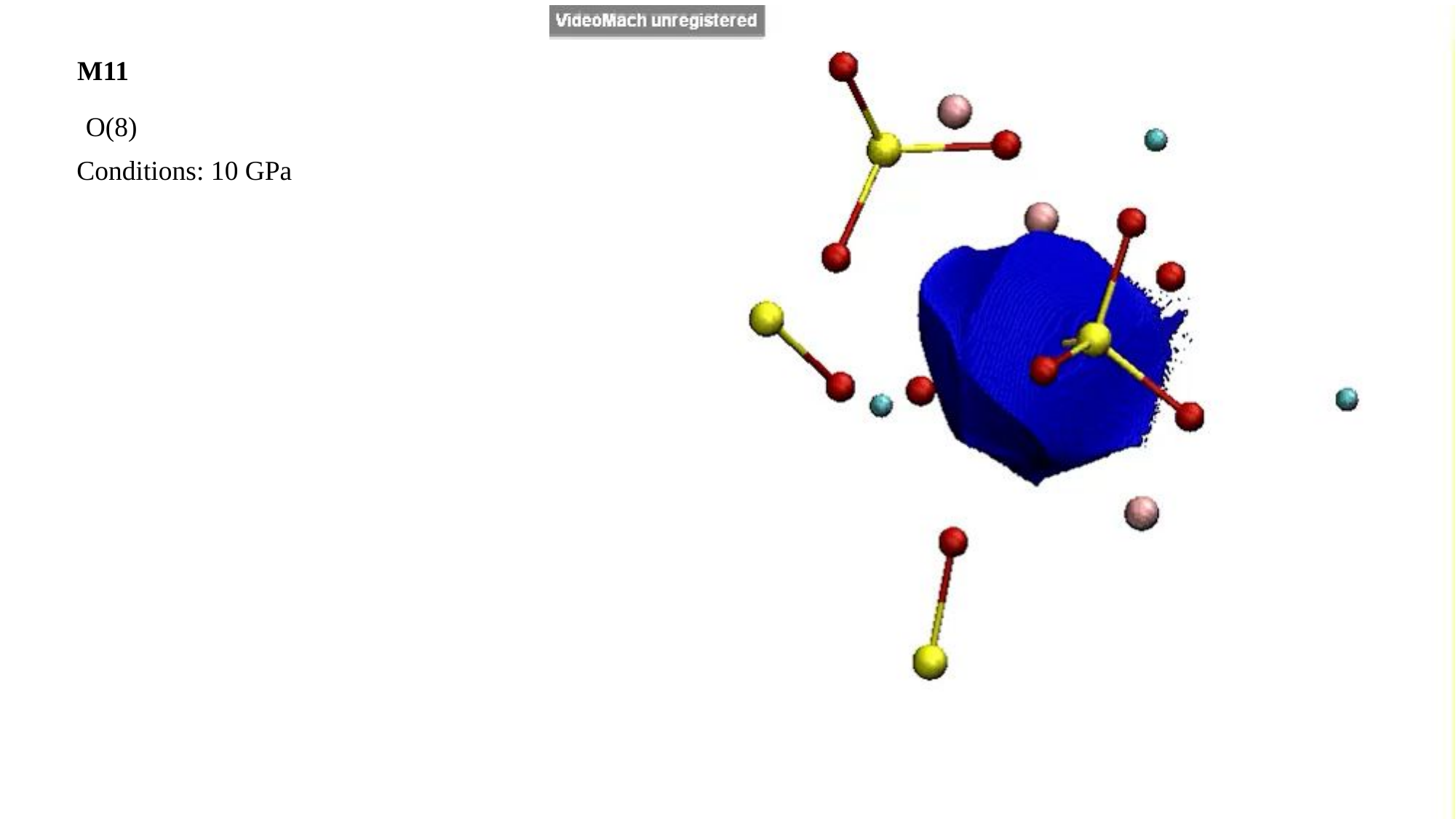

M11
O(8)
Conditions: 10 GPa

## Slide 13
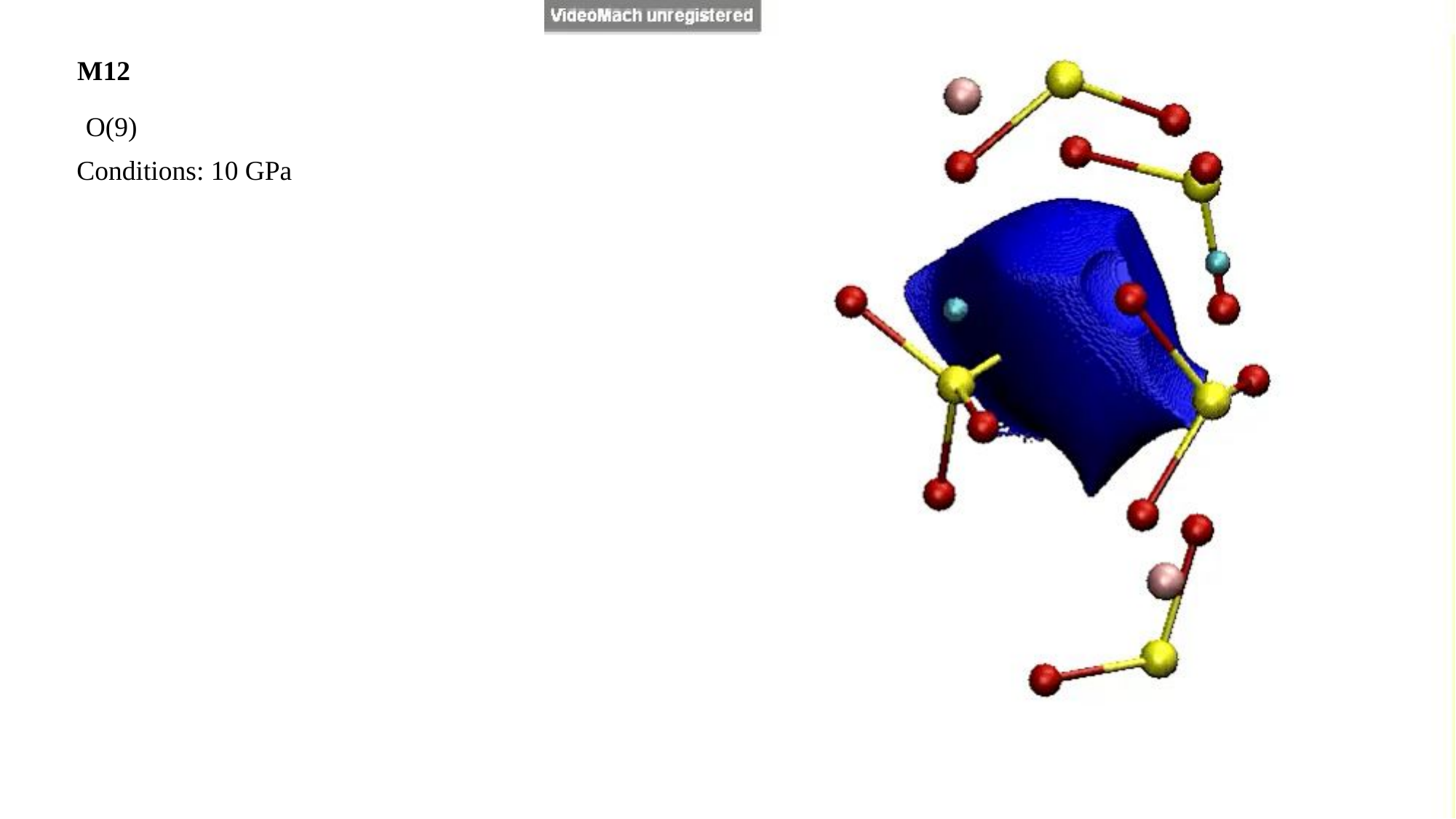

M12
O(9)
Conditions: 10 GPa

## Slide 14
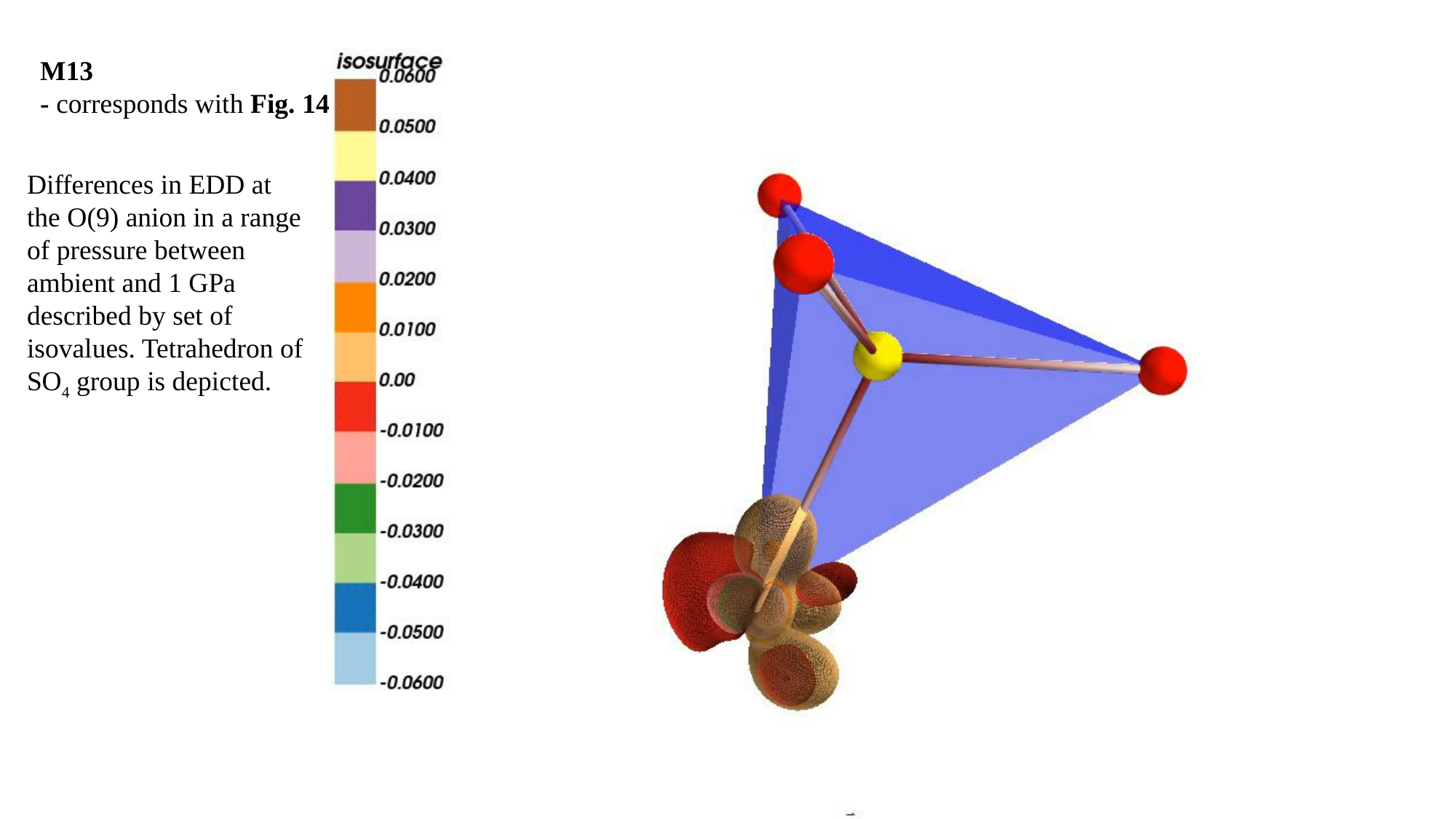

M13
- corresponds with Fig. 14
Differences in EDD at the O(9) anion in a range of pressure between ambient and 1 GPa described by set of isovalues. Tetrahedron of SO4 group is depicted.
