# Supplementary material for: Tracing electron density changes in langbeinite under pressure
Source: IUCrJ. 2021 Dec 23;9(Pt 1):146–62. doi: 10.1107/S2052252521012628 (PMC8733888; doi:10.1107/S2052252521012628)
Supplement: Supplementary file 19 [file m-09-00146-sup19.pdf]

IUCrJ

Volume 9 (2022)

Supporting information for article:

Tracing electron density changes in langbeinite under pressure

Roman Gajda, Dongzhou Zhang, Jan Parafiniuk, Przemysław Dera and Krzysztof Woźniak

S1. Experimental quantitative charge density studies – main ideas.

Commonly used models of experimental charge density are based on a finite spherical harmonic expansion of the electronic part of the charge distribution about each atomic center. Such an atomic expansion is called a pseudoatom and the molecular electron distribution at any point in a crystal is the sum of all the pseudoatomic densities. In the most commonly used formalism of Hansen and Coppens(Hansen & Coppens, 1978; Koritsanszky & Coppens, 2001) the pseudoatom electron density is defined by:

$$\rho(r) = \rho_c(r) + P_V\kappa^3\rho_V(\kappa r) + \sum_{i=0}^{l_{max}} \kappa'^3 R_j(\kappa' r) \sum_{m=0}^{l} P_{lm\pm} d_{lm\pm}(\theta,\varphi)$$

where ρ_c(r) and ρ_v(r) are spherical core and valence densities, respectively. The third term contains the sum of the angular functions $d_{lm\pm}(\theta,\varphi)$ to take into account aspherical deformations. The angular functions $d_{lm\pm}(\theta,\varphi)$ are real spherical harmonic functions. The coefficients P_v and $P_{lm\pm}$ are populations for the valence and deformation density multipoles, respectively. The κ and κ' are scaling parameters introduced to make valence and deformation densities expand or contract. In the Hansen-Coppens formalism the P_v, $P_{lm\pm}$, κ and κ' are refineable parameters together with the atomic coordinates and thermal coefficients. Least-squares refinements are performed against the measured intensities F^2(hkl) of reflections obtained by single crystal X-ray diffraction. This requires resolution of data limited up to 0.45-0.50Å and the full data completeness. Starting atomic coordinates and anisotropic displacement parameters are taken from the ordinary spherical refinement stage and freely refined. The C-H bond distances and thermal motions of H-atoms are usually taken from neutron diffraction studies or fixed (when neutron data are not available) at the averaged neutron distances for similar groups. Each atom is assigned a core and spherical-valence scattering factors derived from Clementi and Roetti wavefunctions.(Clementi & Roetti, 1974) A single-ζ Slater type radial function multiplied by density-normalized spherical harmonics are used for describing the valence deformation terms. The multipole expansion usually is truncated at the hexadecapole level for the non-hydrogen atoms. Usually, only bond-oriented dipole components for H atoms are applied. The radial fit of the valence density is optimized by refinement of the expansion-contraction parameter κ for all non-hydrogen atoms. The valence-deformation radial fits are optimized by refinement of the κ' parameters. The validity of all κ and κ' values are checked against the values obtained from multipole refinement on theoretical structure factors for a series of model compounds.(Volkov *et al.*, 2001) The adequacy of the proper deconvolution of thermal motion from the bonding density for each model is tested by the Hirshfeld rigid-bond test.(Macchi *et al.*, 1998*a*,*b*)

Once such an aspherical atomic electron density ρ(r) is defined, then it can be used to obtain aspherical atomic form factors and aspherical structural factors for a given crystal. The squares of such aspherical

structural factors corrected for numerous experimental effects such as thermal motions, extinction, absorption, TDS when present, *etc*, are proportional to the measured intensities of reflections. When hundreds thousands of reflections (or sometimes even more than million) are measured and used in the refinement, the above mentioned parameters such as the populations of electrons, contraction/expansion coefficients can be obtained.

To acquire good charge density data some special requirements have to be fulfilled. These are: very good quality single crystals, high resolution (> 0.5Å) data to analyse even subtle changes of the electron density, low temperature measurements (100K or even 10K and lower when possible) – although for many mineral crystals this requirement is less important, H-atom treatment (availability of neutron data, proper estimation of hydrogen atom positions and ADPs), accurate and precise intensity measurements with errors small enough not to influence bonding electron density and careful corrections for different technical effects such as absorption, extinction, background and thermal diffuse scattering (TDS), variation of incident beam intensity.

S2. Topological analysis of electron density

Once quantitative electron density distribution in minerals is established, different methods of electron density partitioning can be used to analyse properties of the studied systems. These are, for example, such methods as the stockholder pseudoatom partitioning,(Hirshfeld, 1977) or - the most popular - Atoms-In-Molecules theory (AIM) proposed by R. Bader.(Bader, 1994) The AIM theory(Popelier, 1996) offers a self-consistent way of partitioning any molecular system into its atomic fragments, deduced from the first principles of Quantum Mechanics and Schwinger's principle of stationary action.(Coppens *et al.*, 1979) In the AIM theory, the many electron system is separated into subsystems (atomic basins) by *zero-flux surfaces* (ZFSs) that satisfy the following condition for every point on the surface: $\mathbf{n}\bullet\nabla\rho(\mathbf{r}) = 0$, where $\nabla\rho(\mathbf{r})$ is the gradient vector field of the molecular electron density, $\mathbf{r}$ is a point on the zero-flux surface that separates two fragments, and $\mathbf{n}$ is the vector normal to the surface at that point. Further analysis of the gradient vector field of electron density results in localization of the extremes of the electron density by finding points named *critical points* (CP) at which following equation applies: $\nabla\rho(\mathbf{r}_{CP})=0$. Particularly useful are bond critical points – the weakest points in bonds which define their properties. Integrating these properties over the atomic basins is one of the cornerstones of AIM theory because it yields valuable information such as integrated charges and the volumes of atoms/ions, their energies, electronic populations as well as higher multiple moments polarizabilities, *etc*.(Angyan *et al.*, 1994) Koch and Popelier have utilized Bader's AIM theory to produce specific criteria to characterise weak interactions, and thus classify hydrogen bonds in particular.(Koch & Popelier, 1995) We have verified these using experimental data.(Dominiak *et al.*, 2006) The first four criteria concern bond critical point (BCP) properties: the existence of BCPs, charge

density and laplacian at BCPs and mutual penetration of interacting atoms. Four additional necessary criteria are based on the integrated properties of atoms and concern: loss of charge, destabilisation of atom, decrease of the dipolar depolarization and atomic volumes of the interacting atoms in relation to the non-interacting ones. All the above parameters of electron density are quantitative in nature and are useful descriptors to study electronic effects, intra and intermolecular interactions in minerals.

From experimental electron-density distribution, also the Electron Localisation Function (ELF), introduced by Becke & Edgecombe,(Becke & Edgecombe, 1990) can be derived using the Kirzhnits approximation for the calculation of kinetic energy density,(Kirzhnits, 1957; Tsirelson, 2002) as suggested by Tsirelson & Stash(Tsirelson & Stash, 2002) used for bond classification.(Silvi & Savin, 1994) ELF relates to the Pauli exclusion principle and tends to the value 1, where parallel electron spins are highly improbable and there is a high probability of opposing spin pairs. Its value goes to zero in regions where there is a high probability of same-spin pairs, whereas it tends to 0.5 in those regions where the electrons follow the homogeneous electron gas distribution. Partitioning of the ELF gradient field yields basins that can be associated with bonds and electron lone pairs of atoms. Core basins surround nuclei with atomic numbers higher than two and are labelled C(A), where A stands for an atom. Valence basins are categorized by synaptic order, i.e. the number of valence shells of various atoms they participate in. Monosynaptic basins are labelled V(A), disynaptic basins are labelled V(A,B), where A and B are the atoms to which a given basin contributes.

The reduced density gradient (RDG) is another convenient tool for bonding characterization.(Johnson *et al.*, 2010) It can be calculated directly from electron density and its derivative. This dimensionless quantity describes the deviation from a homogenous electron distribution. In regions where electron density decays to zero exponentially (e.g. far from the nuclei), RDG has large positive values, and in the regions of both covalent and noncovalent interactions, RDG approaches values close to zero. The sign of the laplacian is a widely used tool in the interpretation of the nature of a chemical bond, especially in the case of strong interactions.

S3. Independent Atom Model refinement

Table S1 Basic experimental data.

	Ag_exp	Mo_exp	APS_exp
Crystal data			
Chemical formula	$K_2Mg_2O_{12}S_3$	$K_2Mg_2O_{12}S_3$	$K_2Mg_2O_{12}S_3$
Mr	415.00	415.00	415.00
Crystal system, space group	Cubic, $P2_13$	Cubic, $P2_13$	Cubic, $P2_13$
Temperature (K)	293	297	293
a (Å)	9.91895 (2)	9.91977 (3)	9.90450 (7)
V (Å3)	975.88 (1)	976.12 (1)	971.62 (2)
Z	4	4	4
F(000)	824	824	824
Dx (Mg m-3)	2.825	2.824	2.837
Radiation type	Ag Kα, λ = 0.56087 Å	Mo K, λ = 0.7107	synchrotron, λ = 0.434 Å
No. of reflections for cell measurement	80569	28935	2862*
θ range (°) for cell measurement	2.8–44.4	3.5–64.9	2.2-27.7*
μ (mm-1)	0.91	1.81	0.45
Crystal size (mm)	0.50 × 0.43 × 0.28	0.21 × 0.10 × 0.04	
Data collection			
Diffractometer	SuperNova, Single source at offset/far, Eos	SuperNova, Single source at offset/far, Eos	Esperanto-CrysAlis PRO-abstract goniometer imported esperanto images
Radiation source	micro-focus sealed X-ray tube, SuperNova (Ag) X-ray Source	micro-focus sealed X-ray tube, SuperNova (Mo) X-ray Source	synchrotron
Monochromator	Mirror	Mirror	synchrotron
Detector resolution (pixels mm-1)	16.0128	16.0026	5.8140
Scan method	ω scans	ω scans	ω scans
Absorption correction	Gaussian CrysAlis PRO	Gaussian CrysAlis PRO	Multi-scan CrysAlis PRO

	1.171.40.67a (Rigaku Oxford Diffraction, 2019) Numerical absorption correction based on gaussian integration over a multifaceted crystal model Empirical absorption correction using spherical harmonics, implemented in SCALE3 ABSPACK scaling algorithm.	1.171.40.67a (Rigaku Oxford Diffraction, 2019) Numerical absorption correction based on gaussian integration over a multifaceted crystal model Empirical absorption correction using spherical harmonics, implemented in SCALE3 ABSPACK scaling algorithm.	1.171.40.67a (Rigaku Oxford Diffraction, 2019) Empirical absorption correction using spherical harmonics, implemented in SCALE3 ABSPACK scaling algorithm.
Tmin, Tmax	0.217, 1.000	0.616, 1.000	0.832, 1.000*
No. of measured, independent and observed [I > 2σ(I)] reflections	168589, 5360, 5292	63785, 5785, 5569	3008, 3008, 2712
Rint	0.031	0.031	0.045*
θ values (°)	θmax = 44.6, θmin = 2.3	θmax = 66.0, θmin = 2.9	θmax = 27.7, θmin = 1.8
(sin θ/λ)max (Å-1)	1.252	1.285	1.071
Range of h, k, l	h = -24→24, k = -24→24, l = -24→24	h = -24→25, k = -25→21, l = -24→24	*h = -21→11, k = -8→13, l = -11→15
Refinement			
Refinement on	F2	F2	F2
R[F2 > 2σ(F2)], wR(F2), S	0.022, 0.060, 1.10	0.022, 0.059, 1.06	0.033, 0.076, 1.03
No. of reflections	5360	5785	3008
No. of parameters	58	58	58
No. of restraints	0	0	0
Weighting scheme	w = 1/[□2(Fo2) + (0.0284P)2 + 0.1847P] where P = (Fo2 + 2Fc2)/3	w = 1/[□2(Fo2) + (0.0272P)2 + 0.1257P] where P = (Fo2 + 2Fc2)/3	w = 1/[□2(Fo2) + (0.0267P)2] where P = (Fo2 + 2Fc2)/3
(Δ/σ)max	0.001	0.001	0.001
Δ>max, Δ>min (e Å-3)	0.91, -0.41	0.90, -0.54	0.73, -0.64

Absolute structure	Flack x determined using 2346 quotients [(I+)-(I-)]/[(I+)+(I-)] (Parsons, Flack and Wagner, Acta Cryst. B69 (2013) 249-259).	Flack x determined using 2444 quotients [(I+)-(I-)]/[(I+)+(I-)] (Parsons, Flack and Wagner, Acta Cryst. B69 (2013) 249-259).	Flack x determined using 596 quotients [(I+)-(I-)]/[(I+)+(I-)] (Parsons, Flack and Wagner, Acta Cryst. B69 (2013) 249-259).
Absolute structure parameter	0.023 (9)	0.002 (9)	0.11 (7)

* Data for the component 1

S4. Completeness of experimental data

Answering questions about data completeness seems to be very important. First of all, how incomplete data set affects properties of charge density distributions? Secondly, should the data be absolutely complete to obtain reasonable experimental charge density distributions?

The issue of incomplete data concerns in this study only the experiment conducted at the synchrotron facility (see **Table S4**). Other experimental data have 100% completeness and theoretical calculations were conducted on the basis of set of theoretical structure factors which correspond with data resolutions obtained at the synchrotron. The lists of reflections for theoretical structure factors were complete. In general, it is obvious that complete X-ray diffraction data sets should be collected. However, the fact that there is a lack of some reflections in the data set does not necessarily mean that that dataset is completely spoiled. To check whether one can trust in such data, a benchmark test should be performed. In this case, we used refinements for experiments on in-house diffractometers **(Ag_exp** data**)**. Comparison between numerical data (properties at BCPs, properties of integrated atomic basins) show that the results obtained on the basis of incomplete synchrotron data corresponds these with benchmarks.

Below we present the results of refinements conducted for experimental data **Ag_exp**. We took 100% complete data set as a starting point and then, step by step, the size of this dataset was randomly reduced (e.g. 90%, 80%, 70%, 60% and 50%). Reflections were rejected from the whole resolution range with the use of a function which completely randomized this process. Table S15 contains the values of rho and the laplacian at BCPs and Table S16 the volumes and charges of the integrated atomic basins. Values of rho at BCPs look quite stable up to 60% of completeness. At 50% completeness, one Mg-O contact was not found. The values of the laplacian were fluctuated much more, as it is more sensitive to these changes. However, the gradual degradation of completeness does not seem to cause any visible trends for electron density or the laplacian at BCP values. Closer to 50% completeness some small effects may be visible. This is due mostly to larger scatter values for the analysed parameters compared to the 100% completeness case. However the effect seems to be smaller than expected. This is particularly important for experimental charge density investigations of crystals under pressure when DACs often reduce the completeness of data particularly for the lower symmetry crystal systems. When we take into consideration charges of the integrated atomic basins we see that even for 60% of completeness, the total charge of the whole unit cell is still close to 0. However, we want to stress that also parameters of individual ions/atoms should be carefully analysed. In the case of this mineral, just for 80% of completeness some results for particular ions begin to appear a bit odd (K(1) +1.48, O(6) -0.83). That is why, in this case biased results begin to appear at 80% data completeness.

A visualisation of different trends for particular electron density parameters is presented in Tables S15 and S16 (Supplementary Materials) and is shown in Fig. S1. The scatter of atomic volumes (Fig S1 a) increases with the decreasing completeness of data. Integrated atomic charge is quite stable down to a completeness of *ca.* 80% (Fig S1 b). Parameters at BCPs are well known to be very stable (Figs S1 c - S1 f) and they do not change much particularly for completeness above 80%. Isotropic ADPs values (and ADPs in general) seem to be practically independent of completeness (Figs S1 g and S1 h).

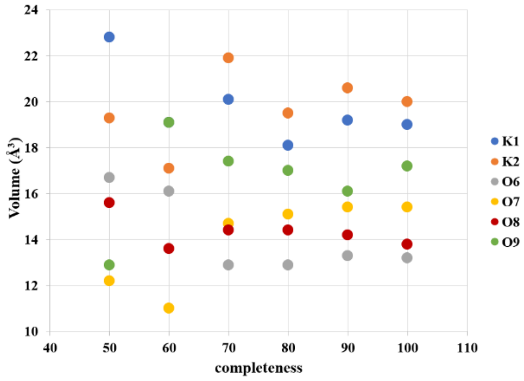

(a)

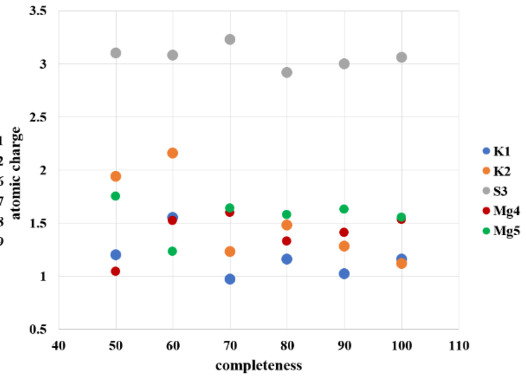

(b)

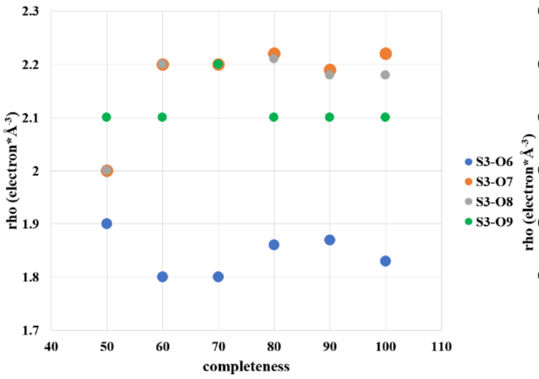

(c)

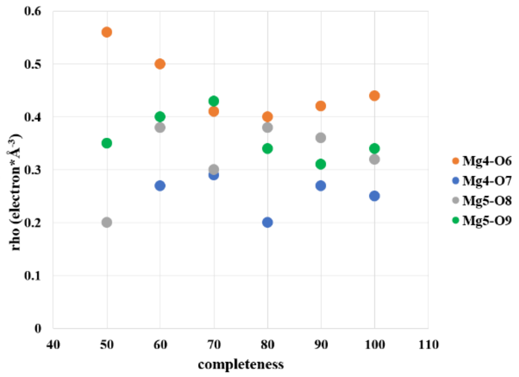

(d)

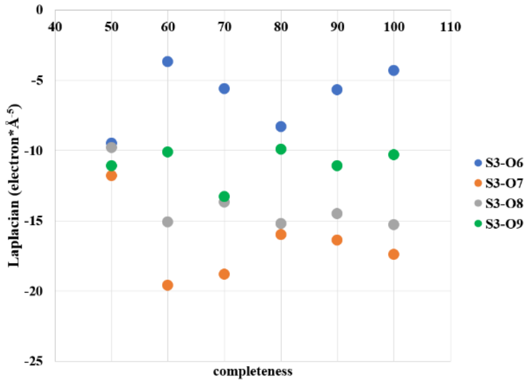

(e)

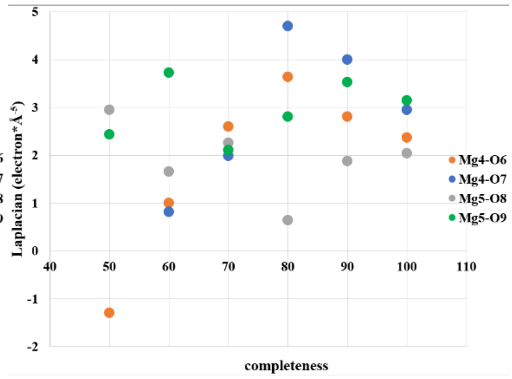

(f)

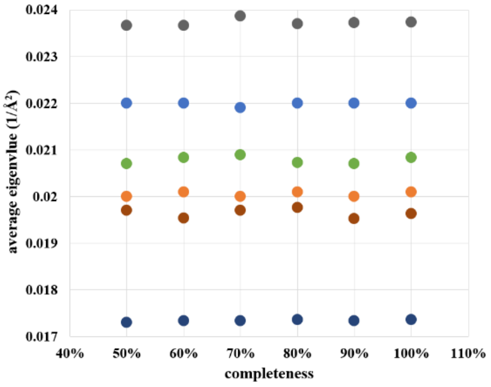

(g)

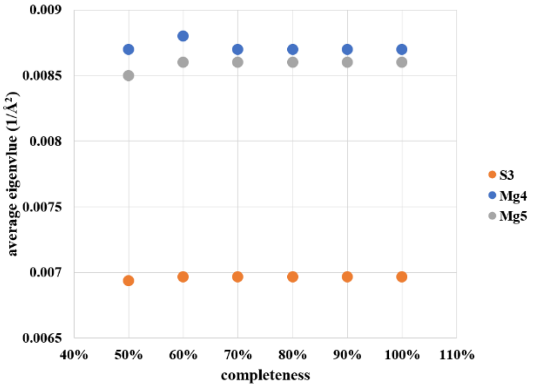

(h)

Figure S1 Different atomic and electronic parameters versus completeness of **Ag_exp** data: (a) atomic volumes; (b) atomic charge; (c) rho at BCPs for SO_4 anion; (d) rho at BCPs for Mg…O contacts; (e) laplacian values at BCPs for S-O bonds; (f) laplacian values at BCPs for Mg…O contacts; (g) isotropic ADPs for potassium and oxygen atoms/ions and (h) isotropic ADP values for S and Mg atoms/ions.

Table S2 **Mo_exp** - Distribution of measured and missing reflections in equal-volume resolution shells.

Shell s_max	Shell d_min	n_hkl measured	n_hkl missing	Percent completeness
0.4734	1.056	292	0	100.0
0.5965	0.838	297	0	100.0
0.6828	0.732	286	0	100.0
0.7515	0.665	298	0	100.0
0.8095	0.618	278	0	100.0
0.8603	0.581	295	0	100.0
0.9056	0.552	284	0	100.0
0.9468	0.528	286	0	100.0
0.9848	0.508	310	0	100.0
1.0200	0.490	280	0	100.0
1.0529	0.475	290	0	100.0
1.0839	0.461	307	0	100.0
1.1132	0.449	265	0	100.0
1.1410	0.438	291	0	100.0
1.1676	0.428	294	0	100.0
1.1929	0.419	290	0	100.0
1.2173	0.411	282	0	100.0
1.2407	0.403	304	0	100.0
1.2633	0.396	278	0	100.0
1.2851	0.389	278	33	89.4

Table S3 **Ag_exp** - Distribution of measured and missing reflections in equal-volume resolution shells.

Shell s_max	Shell d_min	N_hkl measured	N_hkl missing	Percent completeness
0.4613	1.084	272	0	100.0
0.5812	0.860	269	0	100.0
0.6653	0.752	282	0	100.0
0.7322	0.683	268	0	100.0
0.7888	0.634	251	0	100.0
0.8382	0.597	279	0	100.0
0.8824	0.567	273	0	100.0
0.9226	0.542	259	0	100.0
0.9595	0.521	267	0	100.0
0.9938	0.503	258	0	100.0
1.0259	0.487	296	0	100.0
1.0561	0.473	250	0	100.0
1.0846	0.461	279	0	100.0
1.1118	0.450	265	0	100.0
1.1376	0.440	274	0	100.0
1.1624	0.430	261	0	100.0
1.1861	0.422	258	0	100.0
1.2089	0.414	268	0	100.0
1.2309	0.406	290	0	100.0
1.2521	0.399	241	2	99.2

Table S4 **APS_exp** - Distribution of measured and missing reflections in equal-volume resolution shells.

Shell s_max	Shell d_min	N_hkl measured	N_hkl missing	Percent completeness
0.3945	1.267	172	0	100.0
0.4971	1.006	160	0	100.0
0.5690	0.879	178	0	100.0
0.6263	0.798	168	0	100.0
0.6746	0.741	158	1	99.4
0.7169	0.697	172	0	100.0
0.7547	0.663	162	2	98.8
0.7890	0.634	167	2	98.8
0.8206	0.609	163	2	98.8
0.8500	0.588	172	0	100.0
0.8774	0.570	174	3	98.3
0.9032	0.554	141	5	96.6
0.9276	0.539	162	1	99.4
0.9508	0.526	178	5	97.3
0.9730	0.514	162	8	95.3
0.9941	0.503	144	12	92.3
1.0144	0.493	154	10	93.9
1.0339	0.484	127	53	70.6
1.0527	0.475	63	101	38.4
1.0709	0.467	31	124	20.0

S5. Multipole model refinement

Table S5 Selected details of multipole refinement results based on theoretical structure factors.

pressure	ambient	1 GPa	5 GPa
Refinement on, parameters, reflections	F^2 / 223 / 3357	F^2 / 223 / 3357	F^2 / 223 / 3357
R[F2 > 2σ (F2)], R(all)	0.0087, 0.0087	0.0086, 0.0086	0.0089, 0.0089
wR[F2 > 2σ (F2)]	0.0130	0.0126	0.0132
Weighting scheme	$w = 1/[\sigma^2(Fo^2)]$	$w = 1/[\sigma^2(Fo^2)]$	$w = 1/[\sigma^2(Fo^2)]$
$(\Delta/\sigma)_{max}$	1.00326	0.01509	0.64096
$\Delta\rangle_{max}$, $\Delta\rangle_{min}$ (e Å^{-3})	0.160, -0.150	0.147; -0.098	0.156, -0.117

pressure	10 GPa	20 GPa	40 GPa
Refinement on, parameters, reflections	F^2 / 223 / 3357	F^2 / 223 / 3357	F^2 / 223 / 3357
R[F2 > 2σ (F2)], R(all)	0.0094, 0.0094	0.0095, 0.0095	0.0097, 0.0097
wR[F2 > 2σ (F2)]	0.0139	0.0143	0.0148
Weighting scheme	$w = 1/[\sigma^2(Fo^2)]$	$w = 1/[\sigma^2(Fo^2)]$	$w = 1/[\sigma^2(Fo^2)]$
$(\Delta/\sigma)_{max}$	0	0.00068	0
$\Delta\rangle_{max}$, $\Delta\rangle_{min}$ (e Å^{-3})	0.172; -0.170	0.222; -0.192	0.283; -0.281

S6. Interionic distances

Table S6 Interionic distances (Å) in the structures of langbeinite obtained experimentally (synchrotron and in-house diffractometer). Results after multipole refinement.

	Ag_exp	Mo_exp	APS_exp
K(1) – O(6)	2.9915(6)	2.9922(10)	2.9822(9)
K(1) – O(7)	2.8377(8)	2.8380(8)	2.8422(9)
K(1) – O(9)	3.1376(8)	3.1390(7)	3.120(2)
K(2) – O(6)	3.0051(9)	3.0077(7)	3.0073(11)
K(2) – O(8)	2.8669(8)	2.8712(7)	2.867(2)
K(2) – O(9)	3.1058(8)	3.1102(8)	3.099(3)
S(3) – O(6)	1.4628(4)	1.4623(4)	1.4630(8)
S(3) – O(7)	1.4686(4)	1.4677(4)	1.4682(7)
S(3) – O(8)	1.4605(4)	1.4605(4)	1.4601(15)
S(3) – O(9)	1.4670(4)	1.4671(5)	1.4709(16)
Mg(4) – O(6)	2.0606(5)	2.0610(5)	2.0569(9)
Mg(4) – O(7)	2.0765(5)	2.0768(5)	2.0733(8)
Mg(5) – O(8)	2.0487(5)	2.0489(5)	2.0492(16)
Mg(5) – O(9)	2.0646(5)	2.0640(5)	2.0614(17)

Table S7 Interionic distances (Å) in the structures of langbeinite obtained on the basis of theoretically calculated dynamic structure factors.

	ambient	1 GPa	5 GPa	10 GPa	20 GPa	40 GPa
K(1) – O(6)	2.9431(6)	2.9162(6)	2.8286(4)	2.7387(4)	2.6008(4)	2.4626(4)
K(1) – O(7)	2.8441(5)	2.8350(4)	2.7980(4)	2.7618(4)	2.7243(4)	2.6964(5)
K(1) – O(9)	3.0875(7)	3.0585(5)	2.9747(4)	2.9052(4)	2.8595(4)	2.8249(4)
K(2) – O(6)	3.0346(6)	3.0255(6)	2.9845(5)	2.9362(5)	2.8627(4)	2.7742(4)
K(2) – O(8)	2.9258(6)	2.9137(5)	2.8997(5)	2.8964(4)	2.9055(4)	2.8711(4)
K(2) – O(9)	2.9875(6)	2.9684(6)	2.8789(5)	2.7908(5)	2.6607(4)	2.5342(4)
S(3) – O(6)	1.5376(3)	1.5364(3)	1.5304(3)	1.5236(3)	1.5118(3)	1.4919(3)
S(3) – O(7)	1.5460(3)	1.5450(3)	1.5407(3)	1.5367(3)	1.5314(3)	1.5233(3)
S(3) – O(8)	1.5256(3)	1.5233(3)	1.5156(3)	1.5081(3)	1.4984(3)	1.4893(3)
S(3) – O(9)	1.5454(4)	1.5447(3)	1.5414(3)	1.5381(3)	1.5309(3)	1.5144(3)
Mg(4) – O(6)	2.0572(6)	2.0464(4)	2.0153(4)	1.9897(3)	1.9621(3)	1.9176(3)
Mg(4) – O(7)	2.0722(6)	2.0684(4)	2.0521(4)	2.0372(4)	2.0211(4)	1.9697(4)
Mg(5) – O(8)	2.0119(3)	2.0025(3)	1.9728(3)	1.9486(3)	1.9300(3)	1.9198(3)
Mg(5) – O(9)	2.0434(4)	2.0339(4)	2.0079(3)	1.9486(3)	1.9725(3)	1.9462(3)

S7. Comparison of ADPs and Similarity Index

Table S8 Experimental ADPs for atoms in the langbeinite structures at 1 GPa (APS_exp) and ambient pressure (Ag_exp, Mo_exp).

	Ag_exp					
ADPs	U_{11}	U_{22}	U_{33}	U_{12}	U_{13}	U_{23}
K(1)	0.02202(4)	0.02202(4)	0.02202(4)	0.00217(11)	-0.00217(11)	0.00125(11)
K(2)	0.02010(5)	0.02010(5)	0.02010(5)	-0.00486(11)	-0.00486(11)	0.00486(11)
S(3)	0.00703(3)	0.00713(3)	0.00688(3)	0.000557(19)	0.00109(2)	-0.00004(2)
Mg(4)	0.00876(4)	0.00876(4)	0.00876(4)	-0.00018(4)	-0.00018(4)	-0.00018(4)
Mg(5)	0.00862(4)	0.00862(4)	0.00862(4)	-0.00194(4)	-0.00030(4)	-0.00030(4)
O(6)	0.01882(16)	0.0264(2)	0.01709(14)	-0.00871(14)	0.00946(12)	0.00112(13)
O(7)	0.02047(15)	0.02341(17)	0.00813(9)	-0.00663(13)	-0.00302(9)	0.00087(10)
O(8)	0.0263(2)	0.01039(11)	0.02208(17)	-0.00343(12)	0.00404(15)	-0.00725(11)
O(9)	0.0284(2)	0.0250(2)	0.01720(16)	-0.00271(17)	0.01519(16)	-0.00174(14)

	Mo_exp					
ADPs	U_{11}	U_{22}	U_{33}	U_{12}	U_{13}	U_{23}
K(1)	0.02187(4)	0.02187(4)	0.02187(4)	0.00196(12)	-0.00196(12)	0.00116(12)
K(2)	0.01954(4)	0.01954(4)	0.01954(4)	-0.00170(10)	-0.00170(10)	-0.00166(10)
S(3)	0.00703(3)	0.00701(3)	0.00679(3)	0.00054(2)	0.00002(2)	-0.00112(2)
Mg(4)	0.00875(4)	0.00875(4)	0.00875(4)	-0.00006(4)	-0.00006(4)	-0.00044(4)
Mg(5)	0.00861(4)	0.00861(4)	0.00861(4)	-0.00183(4)	-0.00027(4)	-0.00027(4)
O(6)	0.0268(2)	0.0188(2)	0.0170(1)	-0.0087(2)	-0.0010(1)	-0.0094(1)
O(7)	0.0231(2)	0.0206(2)	0.0083(1)	-0.0065(1)	-0.0009(1)	0.0030(1)
O(8)	0.0107(1)	0.0257(2)	0.0224(2)	-0.0033(1)	0.0075(1)	-0.0039(2)
O(9)	0.0287(2)	0.0174(2)	0.0247(2)	0.0154(2)	-0.0024(2)	-0.0015(1)

	APS_exp					
ADPs	U_{11}	U_{22}	U_{33}	U_{12}	U_{13}	U_{23}
K(1)	0.0171(1)	0.0171(1)	0.0171(1)	-0.0008(1)	-0.0008(1)	-0.0008(1)
K(2)	0.0149(1)	0.0149(1)	0.0149(1)	0.0014(1)	0.0014(1)	0.0014(1)
S(3)	0.0029(1)	0.0031(1)	0.0025(1)	-0.0006(1)	0.0012(1)	-0.0001(1)
Mg(4)	0.0043(1)	0.0043(1)	0.0043(1)	-0.0001(1)	-0.0001(1)	-0.0001(1)
Mg(5)	0.0042(1)	0.0042(1)	0.0042(1)	-0.0008(1)	-0.0008(1)	-0.0008(1)
O(6)	0.0145(4)	0.0223(5)	0.0133(4)	0.0087(4)	0.0092(4)	-0.0014(4)
O(7)	0.0168(4)	0.0204(5)	0.0037(3)	0.0071(4)	-0.0034(3)	-0.0007(3)
O(8)	0.0227(5)	0.0064(4)	0.0170(4)	0.0036(4)	0.0039(4)	0.0071(3)
O(9)	0.0121(4)	0.0241(5)	0.0211(5)	-0.0140(4)	0.0020(4)	-0.0030(4)

Table S9 Eigenvalues of ADPs for atoms in the langbeinite structures at 1 GPa (APS_exp) and ambient pressure (Ag_exp, Mo_exp).

	Ag_exp		
ADPs	λ_1	λ_2	λ_3
K(1)	0.01826	0.02453	0.02327
K(2)	0.02982	0.01524	0.01524
S(3)	0.00819	0.00711	0.00574
Mg(4)	0.00838	0.00895	0.00895
Mg(5)	0.01056	0.00659	0.00871
O(6)	0.00607	0.03351	0.02273
O(7)	0.02905	0.01556	0.00740
O(8)	0.00682	0.02085	0.03110
O(9)	0.03969	0.02430	0.00661

	Mo_exp		
ADPs	λ_1	λ_2	λ_3
K(1)	0.01846	0.02412	0.02303
K(2)	0.01617	0.02125	0.02120
S(3)	0.00816	0.00700	0.00567
Mg(4)	0.00829	0.00877	0.00919
Mg(5)	0.01044	0.00670	0.00869
O(6)	0.03373	0.02270	0.00618
O(7)	0.02880	0.01562	0.00758
O(8)	0.00695	0.02098	0.03087
O(9)	0.00665	0.03998	0.02418

	APS_exp		
ADPs	λ_1	λ_2	λ_3
K(1)	0.01550	0.01790	0.01790
K(2)	0.01770	0.01350	0.01350
S(3)	0.00418	0.00290	0.00142
Mg(4)	0.00450	0.00390	0.00450
Mg(5)	0.00240	0.00510	0.00510
O(6)	0.00210	0.02913	0.01887
O(7)	0.02624	0.01186	0.00280
O(8)	0.00272	0.01648	0.02690
O(9)	0.00287	0.03432	0.02012

Table S10 Eigenvalues of ADPs for atoms in the langbeinite structures obtained on the basis of theoretical calculations (conducted on the basis of F^2).

	0_GPa		
ADPs	λ_1	λ_2	λ_3
K(1)	0.05599	0.05134	0.04957
K(2)	0.05767	0.04536	0.03577
S(3)	0.01158	0.01613	0.01769
Mg(4)	0.03117	0.01446	0.01757
Mg(5)	0.01547	0.01963	0.01950
O(6)	0.01278	0.08431	0.06420
O(7)	0.07146	0.03479	0.01645
O(8)	0.08071	0.01409	0.04490
O(9)	0.01387	0.09543	0.05550

	1_GPa		
ADPs	λ_1	λ_2	λ_3
K(1)	0.04700	0.05330	0.04700
K(2)	0.05000	0.03650	0.05000
S(3)	0.01112	0.01552	0.01686
Mg(4)	0.01710	0.02130	0.02130
Mg(5)	0.01460	0.01820	0.01820
O(6)	0.01244	0.07770	0.06056
O(7)	0.06549	0.03287	0.01573
O(8)	0.07115	0.01353	0.04052
O(9)	0.01334	0.08438	0.05189

	5_GPa		
ADPs	λ_1	λ_2	λ_3
K(1)	0.03770	0.04520	0.03770
K(2)	0.03960	0.03030	0.03960
S(3)	0.01009	0.01408	0.01494
Mg(4)	0.01570	0.01900	0.01900
Mg(5)	0.01370	0.01610	0.01610
O(6)	0.01131	0.06418	0.05050
O(7)	0.05481	0.02929	0.01370
O(8)	0.06004	0.01194	0.03112
O(9)	0.01209	0.06468	0.04203

	10_GPa		

ADPs	λ_1	λ_2	λ_3
K(1)	0.04190	0.03200	0.03200
K(2)	0.02650	0.03190	0.03190
S(3)	0.00939	0.01310	0.01390
Mg(4)	0.01830	0.01500	0.01830
Mg(5)	0.01340	0.01460	0.01460
O(6)	0.01054	0.05474	0.04252
O(7)	0.05118	0.02740	0.01263
O(8)	0.05522	0.01100	0.02428
O(9)	0.01134	0.05156	0.03720

	20_gpa		
ADPs	λ_1	λ_2	λ_3
K(1)	0.03870	0.02610	0.02610
K(2)	0.02420	0.02180	0.02420
S(3)	0.00868	0.01208	0.01314
Mg(4)	0.01307	0.01723	0.01710
Mg(5)	0.01494	0.01376	0.01390
O(6)	0.00998	0.04335	0.03207
O(7)	0.04821	0.02458	0.01121
O(8)	0.05768	0.01040	0.01693
O(9)	0.01121	0.03938	0.03002

	40_GPa		
ADPs	λ_1	λ_2	λ_3
K(1)	0.02230	0.02290	0.02230
K(2)	0.01860	0.01740	0.01860
S(3)	0.01164	0.00982	0.00813
Mg(4)	0.01007	0.01693	0.01680
Mg(5)	0.01790	0.01370	0.01370
O(6)	0.00897	0.03440	0.02543
O(7)	0.05820	0.01897	0.00943
O(8)	0.04101	0.00938	0.01281
O(9)	0.00960	0.03166	0.02694

Table S11 Similarity Index calculated for ADPs obtained on the basis of experimental data.

ADPs	APS_exp vs Ag_exp	APS_exp vs Mo_exp	Mo_exp vs Ag_exp
K(1)	1.59	1.49	0.00
K(2)	3.75	2.56	1.65
S(3)	17.83	18.66	0.57
Mg(4)	8.88	8.86	0.02
Mg(5)	10.29	10.34	0.00
O(6)	24.43	34.72	18.30
O(7)	11.68	14.01	1.32
O(8)	22.89	29.72	15.26
O(9)	28.15	38.98	22.90

ADPs	Amb. vs 1_GPa	Amb. vs 5_GPa	Amb. vs 10_GPa	Amb. vs 20_GPa	Amb. vs 40_GPa
K(1)	0.08	1.34	3.05	6.00	12.15
K(2)	0.33	1.27	3.51	8.20	14.46
S(3)	0.04	0.42	0.94	1.65	3.41
Mg(4)	1.79	2.01	2.20	2.85	4.22
Mg(5)	0.09	0.57	1.19	1.49	1.70
O(6)	0.07	0.94	2.51	6.04	10.53
O(7)	0.10	1.02	2.01	3.78	7.78
O(8)	0.18	1.79	4.42	9.84	17.00

S8. Bond Critical Point

Table S12 Electron density, ρ [$\bar{e}$Å^{-3}], and Laplacian, $\nabla^2{}_\rho$ [$\bar{e}$Å^{-5}], at the (3, -1) BCPs for the experimental measurements.

Bond or bonding contact	Ag_exp ρ	Ag_exp (APS completeness)	Mo_exp ρ	APS_exp
	ρ<br>$\nabla^2{}_\rho$	ρ<br>$\nabla^2{}_\rho$	ρ<br>$\nabla^2{}_\rho$	ρ<br>$\nabla^2{}_\rho$
K(1) - O(7)	0.096(9)<br>0.525(7)	0.093(8)<br>0.589(7)	0.064(9)<br>1.115(7)	n/a
K(1) - O(9)	0.15(1)<br>-0.31(1)	0.14(1)<br>-0.28(1)	0.10(1)<br>0.219(9)	n/a
K(2) – O(6)	n/a	n/a	0.10(2)<br>0.37(1)	n/a
K(2) – O(8)	n/a	n/a	0.042(9)<br>1.212(7)	n/a
K(2) - O(9)	0.09(1)<br>0.088(9)	0.08(1)<br>0.21(1)	n/a	0.148(0)<br>0.366(0)
S(3) - O(6)	1.83(8)<br>-4.3(3)	1.84(8)<br>-2.3(3)	2.13(7)<br>-14.9(3)	2.09(8)<br>-13.0(4)
S(3) - O(7)	2.22(7)<br>-17.4(3)	2.21(8)<br>-17.9(3)	2.16(7)<br>-15.8(3)	2.19(8)<br>-9.8(5)
S(3) - O(8)	2.18(7)<br>-15.3(3)	2.12(7)<br>-13.1(3)	2.14(7)<br>-13.0(3)	2.00(7)<br>2.9(4)
S(3) - O(9)	2.1(1)<br>-10.3(4)	2.1(1)<br>-10.4(4)	2.04(7)<br>-11.4(3)	2.00(8)<br>-6.1(4)
Mg(4)…O(6)	0.44(2)<br>2.37(4)	0.45(2)<br>1.98(4)	0.28(2)<br>4.23(3)	0.31(1)<br>5.19(3)
Mg(4)…O(7)	0.25(2)<br>2.95(3)	0.25(2)<br>2.96(4)	0.28(2)<br>4.24(3)	0.25(1)<br>4.18(3)
Mg(5)…O(8)	0.32(2)<br>2.04(3)	0.33(2)<br>1.86(3)	0.28(2)<br>4.09(3)	0.18(2)<br>6.82(2)
Mg(5)…O(9)	0.34(2)<br>3.14(4)	0.33(2)<br>3.09(4)	0.26(2)<br>4.06(3)	0.31(1)<br>4.69(3)

Table S13 Electron density, ρ [$\bar{e}$ Å^{-3}], and laplacian, $\nabla^2{}_\rho$ [$\bar{e}$ Å^{-5}], at the (3, -1) BCPs for theoretical calculations.

Bond/contact	Ambient	1 GPa	5 GPa	10 GPa	20 GPa	40 GPa

	ρ	ρ	ρ	ρ	ρ	ρ
	$\nabla^2\rho$	$\nabla^2\rho$	$\nabla^2\rho$	$\nabla^2\rho$	$\nabla^2\rho$	$\nabla^2\rho$
K(1) – O(6)	0.080(2)	0.070(2)	0.062(3)	0.063(3)	0.091(3)	0.244(5)
	**0.800(2)**	**1.020(2)**	**1.516(3)**	**2.003(3)**	**2.754(3)**	**3.112(3)**
K(1) – O(7)	0.073(2)	0.074(2)	0.079(2)	0.087(3)	0.134(4)	n/a
	**1.095(2)**	**1.333(2)**	**1.641(2)**	**1.816(2)**	**1.817(3)**	
K(1) – O(8)	n/a	n/a	n/a	n/a	n/a	0.121(4)
						**0.888(3)**
K(1) – O(9)	0.059(2)	0.061(2)	0.075(3)	0.083(3)	n/a	n/a
	**0.600(2)**	**0.732(2)**	0.895(2)	1.102(2)		
K(2) - O(6)	n/a	n/a	n/a	n/a	0.107(4)	0.132(3)
					**1.248(2)**	**0.035(3)**
K(2) - O(7)	n/a	0.066(3)	0.069(4)	0.092(4)	0.105(4)	0.174(6)
		**0.341(2)**	**0.587(2)**	**0.785(3)**	**2.064(3)**	**3.768(4)**
K(2) - O(8)	0.076(3)	0.074(3)	0.063(3)	n/a	n/a	n/a
	**0.863(2)**	**0.998(2)**	**1.234(2)**			
K(2) - O(9)	n/a	n/a	0.039(4)	0.055(5)	0.119(6)	0.172(7)
			**1.438(2)**	**1.714(2)**	**2.145(2)**	**2.841(3)**
S(3) - O(6)	1.67(2)	1.65(2)	1.65(2)	1.68(3)	1.73(3)	1.86(3)
	**5.4(1)**	**6.9(1)**	**7.1(1)**	**6.0(1)**	**2.8(1)**	**5.6(1)**
S(3) - O(7)	1.63(2)	1.65(2)	1.67(2)	1.68(2)	1.71(3)	1.68(3)
	**0.2(1)**	**-1.6(1)**	**-2.1(1)**	**-1.5(1)**	**-1.9(1)**	**-1.4(1)**
S(3) - O(8)	1.74(2)	1.74(2)	1.74(2)	1.75(2)	1.81(2)	1.89(3)
	**-0.4(1)**	**-0.3(1)**	**-0.2(1)**	**0.7(1)**	**0.3(1)**	**-3.0(1)**
S(3) - O(9)	1.66(2)	1.65(2)	1.67(2)	1.68(3)	1.72(3)	1.77(3)
	**2.8(1)**	**3.3(1)**	**1.2(1)**	**-1.0(1)**	**-4.2(1)**	**-0.2(1)**
Mg(4)…O(6)	0.178(6)	0.179(6)	0.228(7)	0.248(8)	0.280(9)	0.283(8)
	**5.834(9)**	**6.378(9)**	**6.57(1)**	**7.12(1)**	**7.40(1)**	**6.95(1)**
Mg(4)…O(7)	0.240(3)	0.248(3)	0.281(4)	0.290(6)	0.341(7)	0.359(6)
	**5.07(1)**	**5.223(9)**	**5.03(1)**	**5.45(1)**	**5.65(1)**	**6.26(1)**
Mg(5)…O(8)	0.261(6)	0.269(6)	0.313(6)	0.345(6)	0.386(7)	0.378(8)
	**6.458(9)**	**6.852(9)**	**7.30(1)**	**8.09(1)**	**6.83(1)**	**8.44(2)**
Mg(5)…O(9)	0.173(5)	0.180(5)	0.234(5)	0.242(5)	0.290(4)	0.251(6)
	**6.479(9)**	**6.920(9)**	**7.172(9)**	**7.95(1)**	**7.58(1)**	**9.74(1)**

S9. Atomic basins integration

Table S14 Net atomic charge Ω [$\bar{e}$] resulting from theoretical computations.

		I	II	III	IV	V	VI
		Amb.	1 GPa	5 GPa	10 GPa	20 GPa	40 GPa
K1	Ag	+ 1.16					
	Mo	+ 1.25					
	APS		+ 1.20				
	theor	+ 1.02	+ 1.03	+0.98	+1.06	+1.10	+0.92
K2	Ag	+ 1.08					
	Mo	+ 1.05					
	APS		+ 0.66				
	theor	+ 0.93	+ 0.95	+1.00	+0.99	+0.93	+1.1
S3	Ag	+3.07					
	Mo	+3.15					
	APS		+3.64				
	theor	+ 3.19	+ 3.16	+3.15	+3.15	+3.14	+3.15
Mg4	Ag	+1.51					
	Mo	+1.85					
	APS		+1.79				
	theor	+ 1.91	+ 1.90	+1.83	+1.82	+1.73	+1.90
Mg5	Ag	+ 1.56					
	Mo	+1.90					
	APS		+1.74				
	theor	+ 1.89	+ 1.88	+1.78	+1.78	+1.67	+1.81
O6	Ag	-1.05					
	Mo	-1.23					
	APS		-1.28				
	theor	-1.31	-1.26	-1.18	-1.12	-1.16	-1.44
O7	Ag	-1.10					
	Mo	-1.33					
	APS		-1.03				
	theor	-1.38	-1.40	-1.41	-1.48	-1.41	-1.0
O8	Ag	-1.06					
	Mo	-1.10					
	APS		-1.07				
	theor	-1.49	-1.45	-1.41	-1.38	-1.25	-1.57
O9	Ag	-1.73					
	Mo	-1.43					
	APS		-2.0				
	theor	-1.13	-1.14	-1.21	-1.17	-1.20	-1.19
Total	Ag	-1.20					
	Mo	+ 0.92					
	APS		+ 0.68				
	theor	+1.28	-2.04	-2.36	-1.40	-0.84	-1.68

S10. 2D maps of total electron density, Laplacian, deformation density

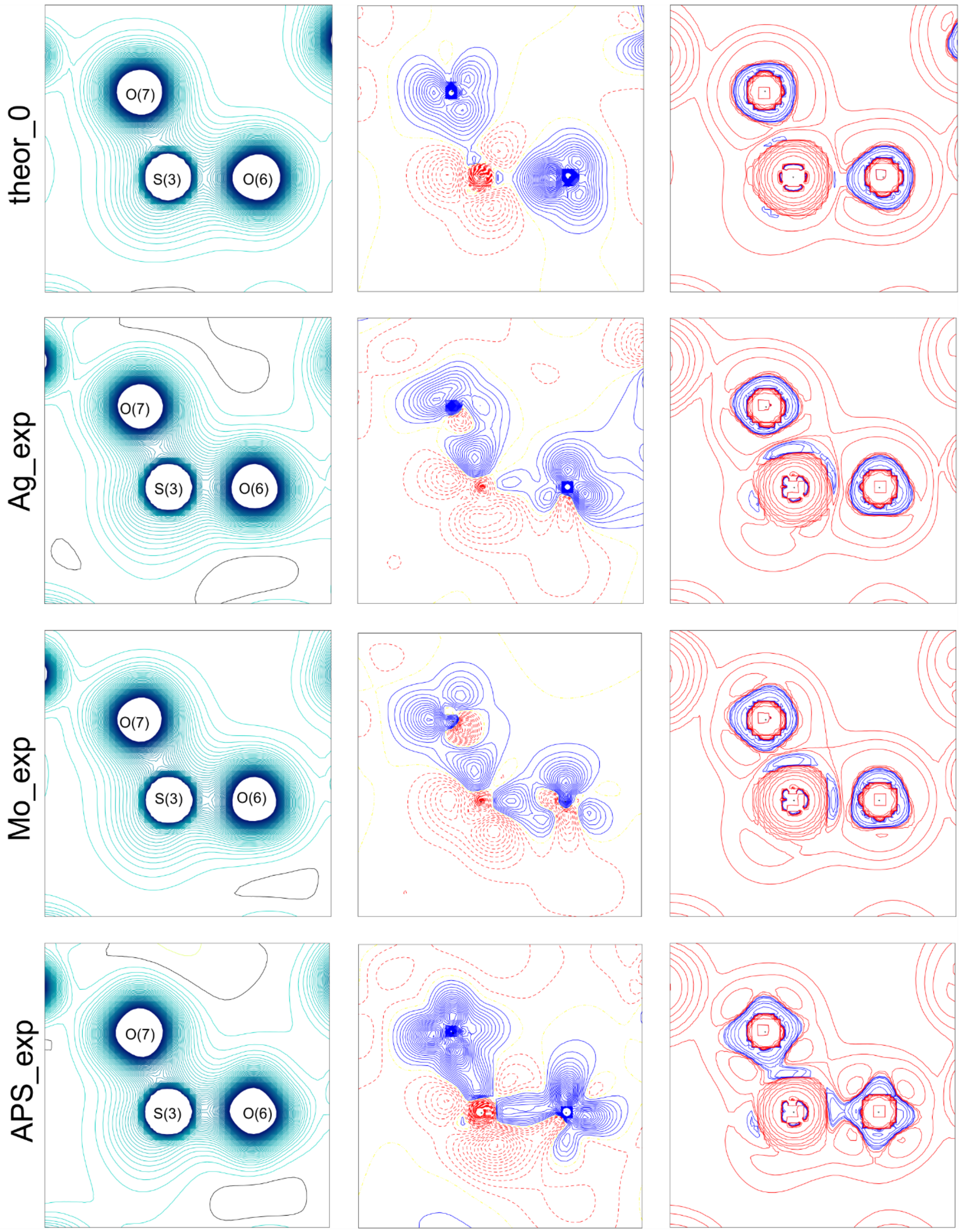

Figure S2 Plane defined by sulphur and oxygen atoms. Maps of total electron density (first column), deformation density (second column) and Laplacian (third column).

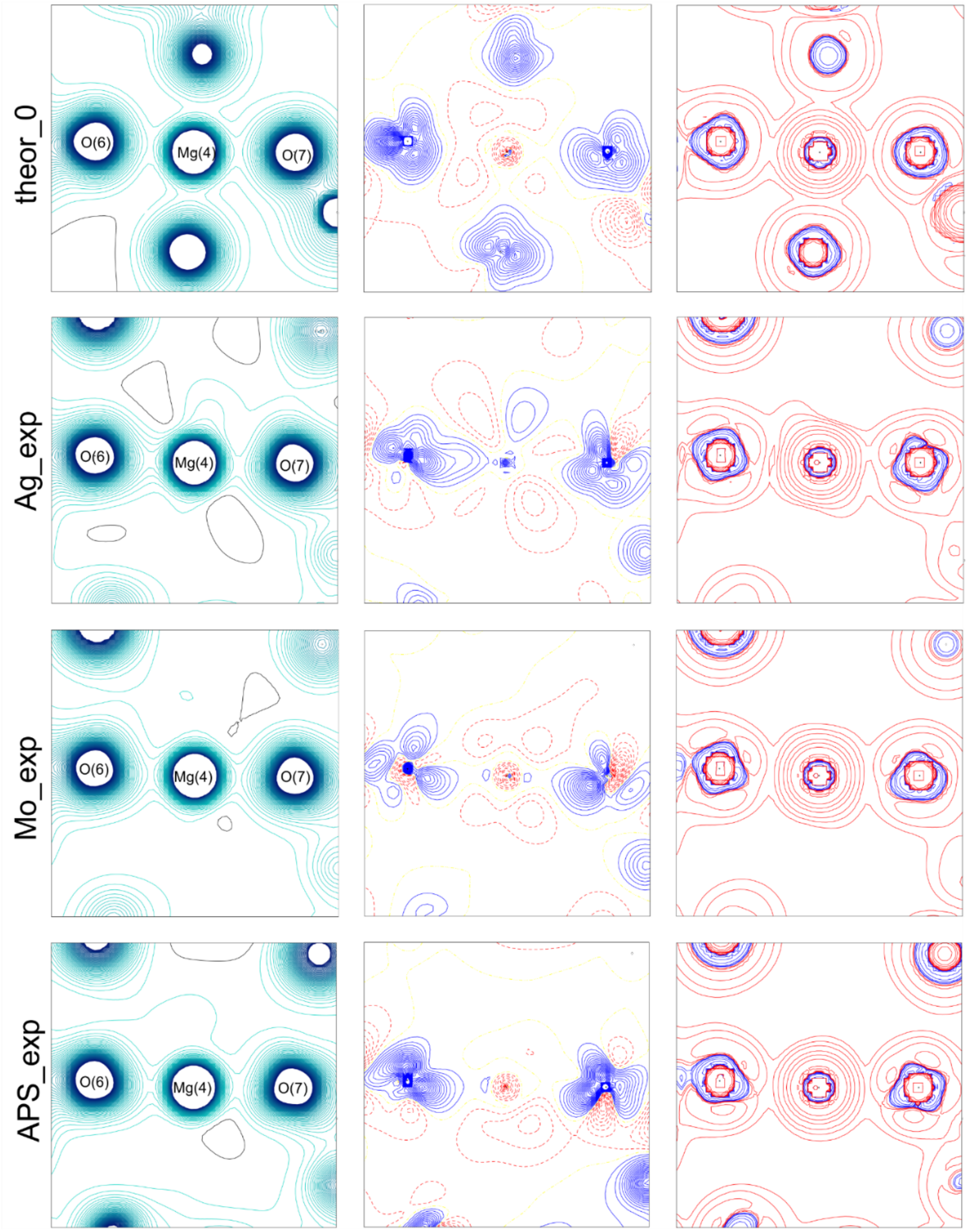

Figure S3 Plane determined by magnesium cation and oxygen anions. Maps of total electron density (first column), deformation density (second column) and Laplacian (third column).

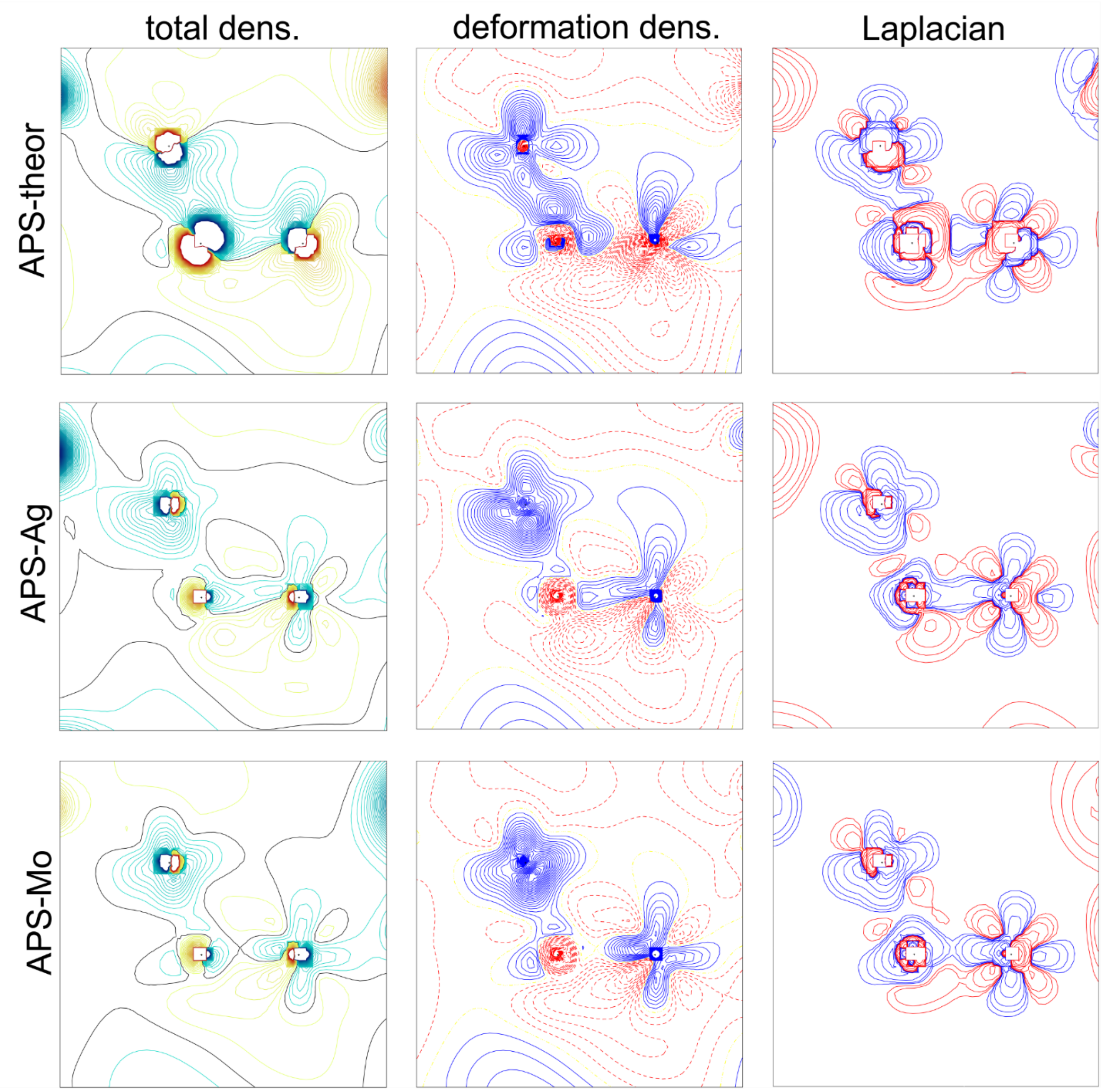

Figure S4 Plane defined by sulphur and oxygen atoms. Maps of difference of: total electron density (first column), deformation density (second column) and Laplacian (third column).

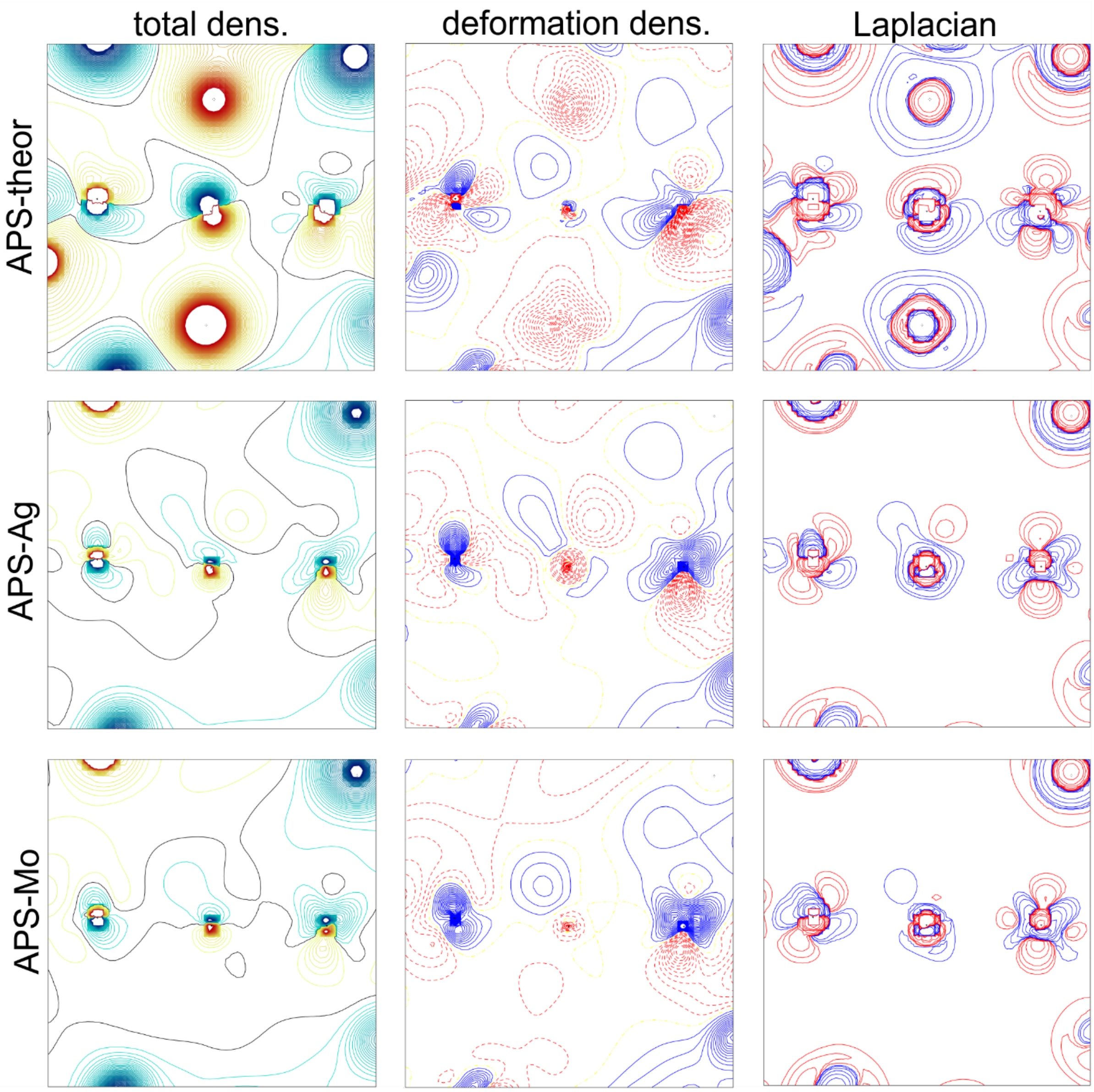

Figure S5 Plane determined by magnesium cation and oxygen anions. Maps of difference of: total electron density (first column), deformation density (second column) and Laplacian (third column).

S11. Atomic basins under pressure

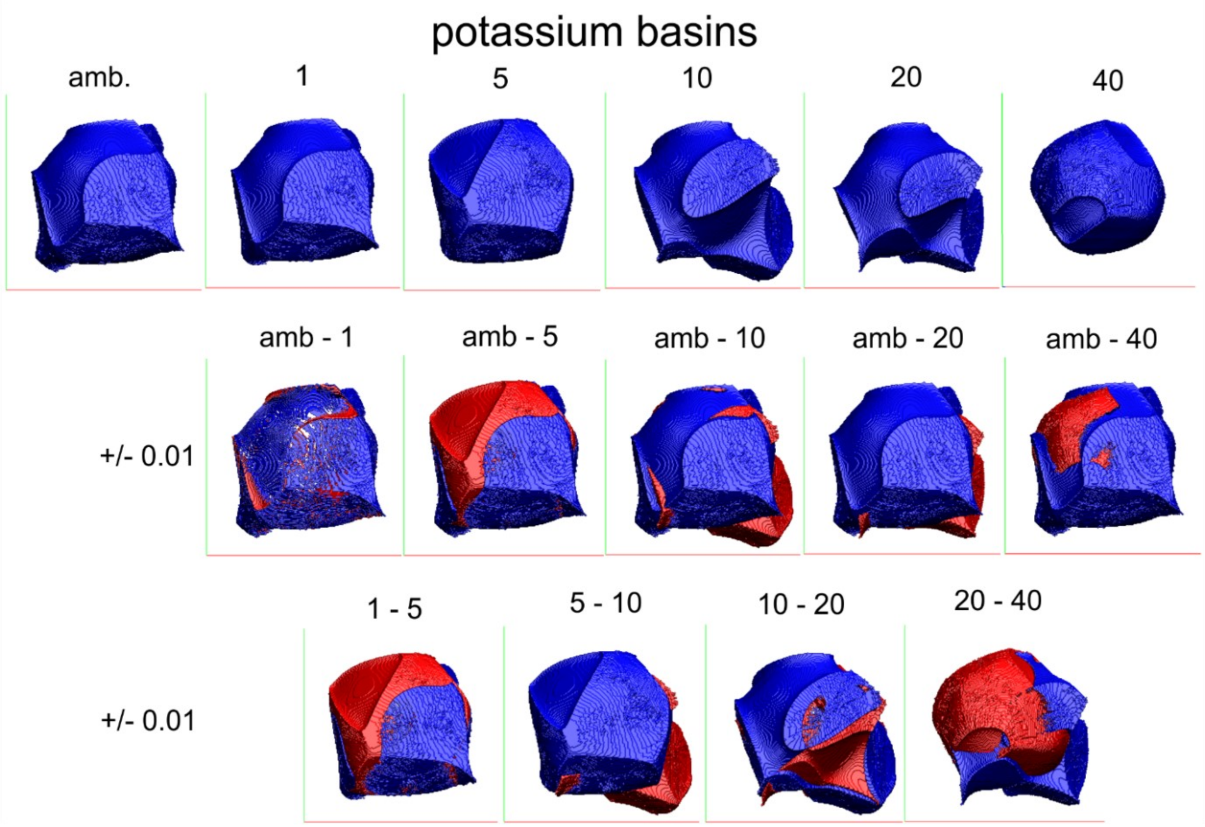

Figure S6 Atomic basins of K(1) cation and its changes under pressure (first row). Differences between atomic basin at ambient pressure and at other pressures – subtracted and superimposed (second row, blue - plus, red - minus). Differences between pairs of pressures.

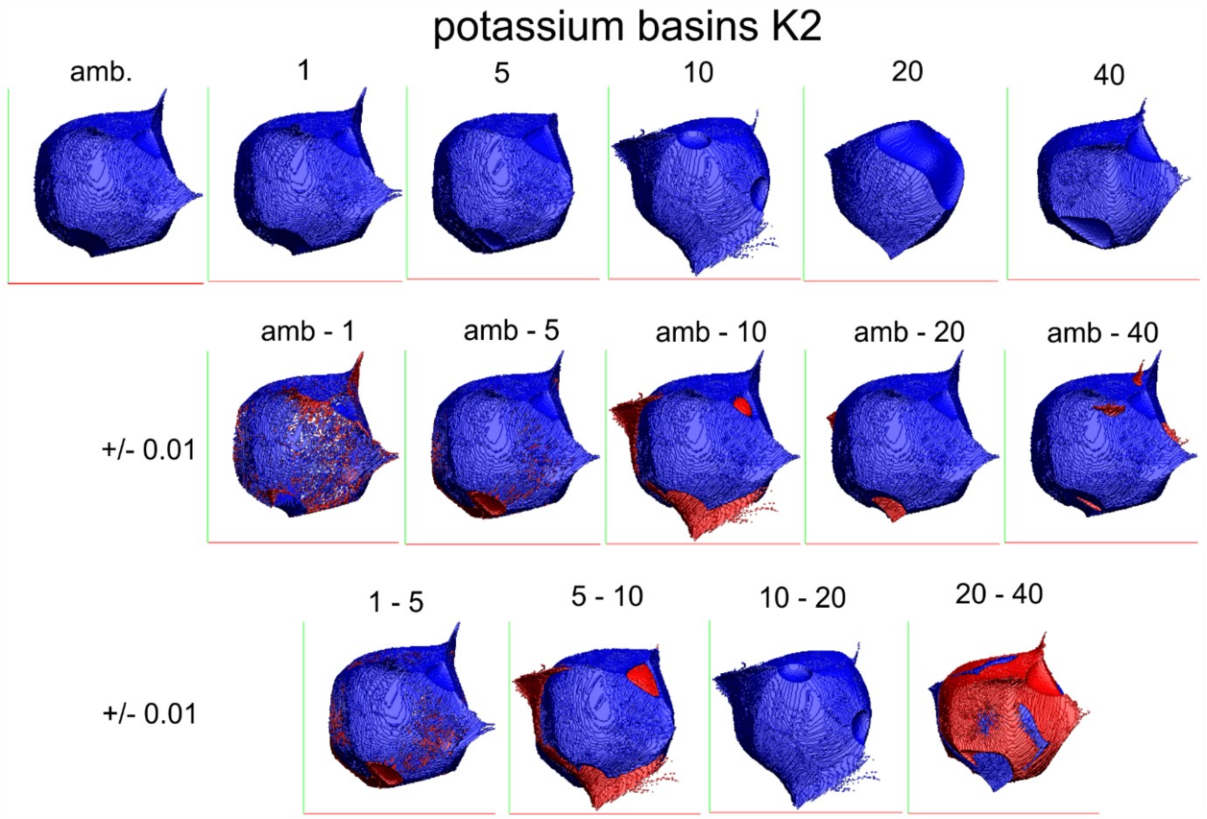

Figure S7 Atomic basins of K(2) cation and its changes under pressure (first row). Differences between atomic basin at ambient pressure and at other pressures – subtracted and superimposed (second row, blue - plus, red - minus). Differences between pairs of pressures.

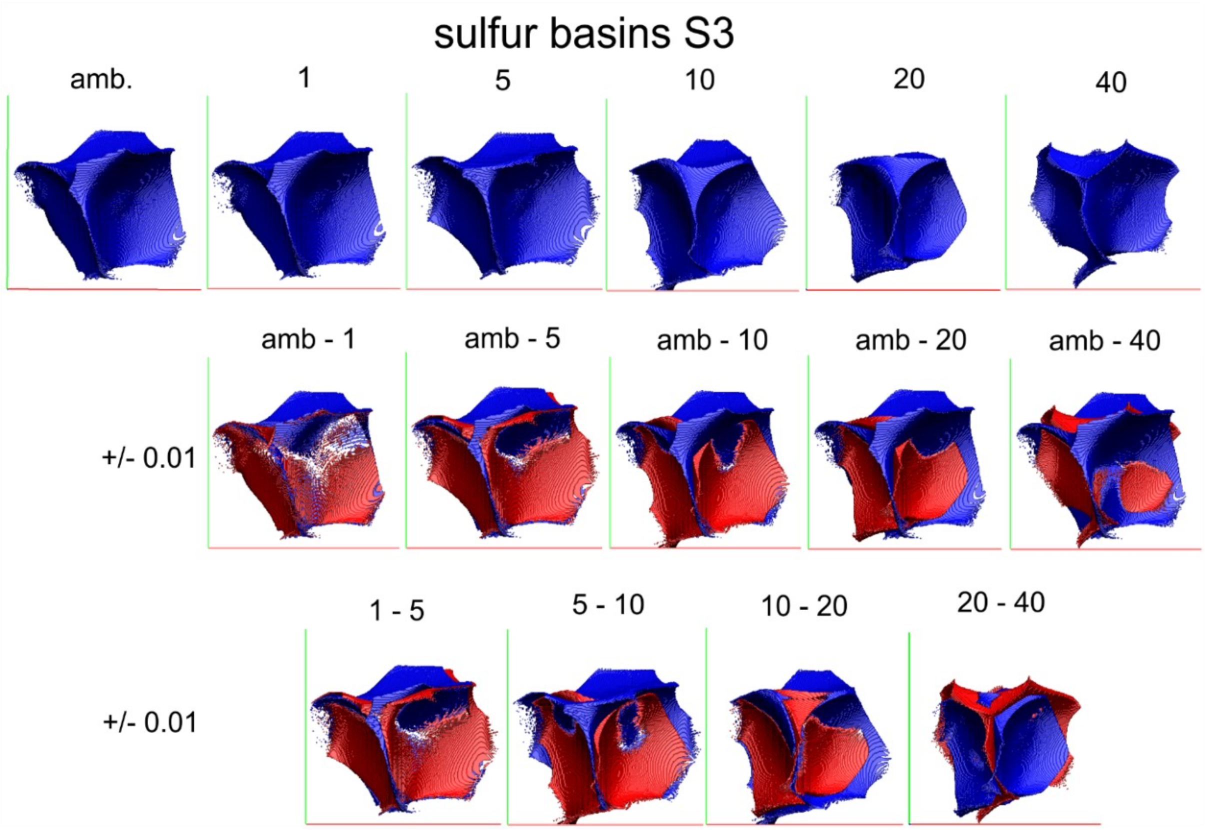

Figure S8 Atomic basins of S(3) cation and its changes under pressure (first row). Differences between atomic basin at ambient pressure and at other pressures – subtracted and superimposed (second row, blue - plus, red - minus). Differences between pairs of pressures.

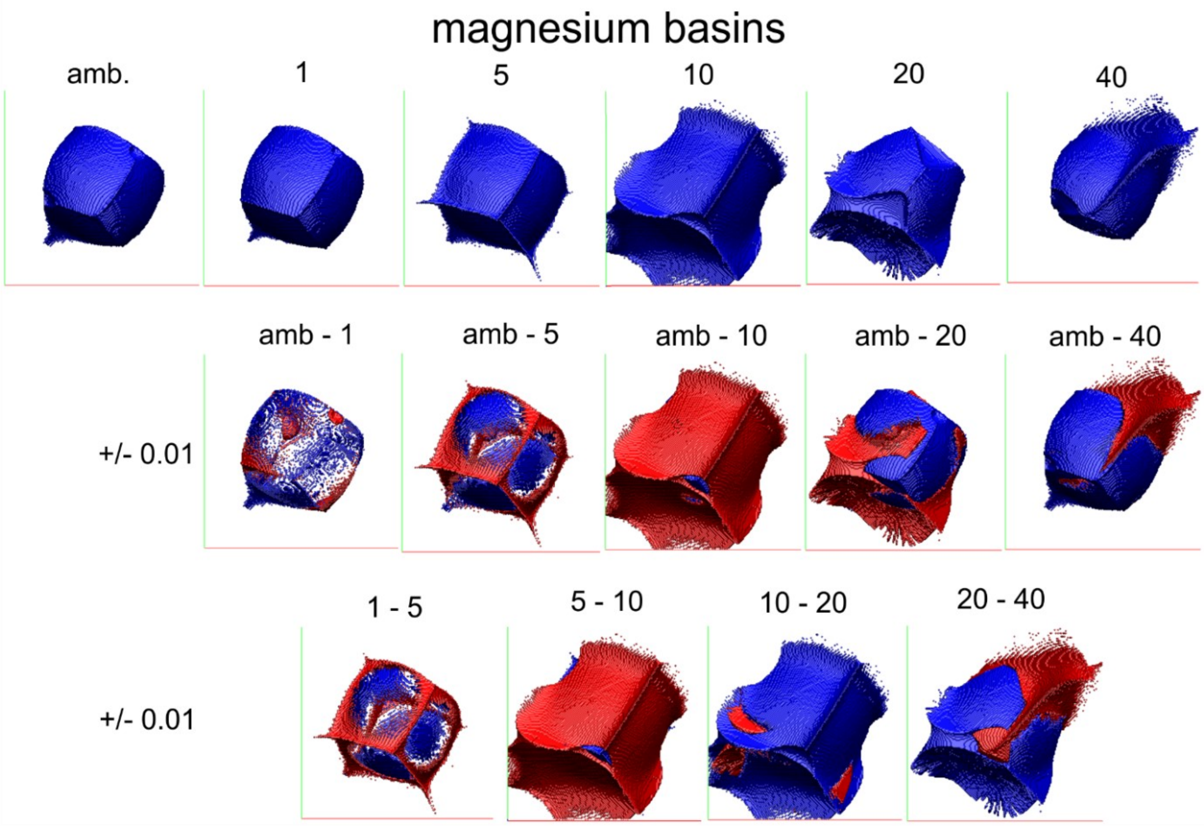

Figure S9 Atomic basins of Mg(4) cation and its changes under pressure (first row). Differences between atomic basin at ambient pressure and at other pressures – subtracted and superimposed (second row, blue - plus, red - minus). Differences between pairs of pressures.

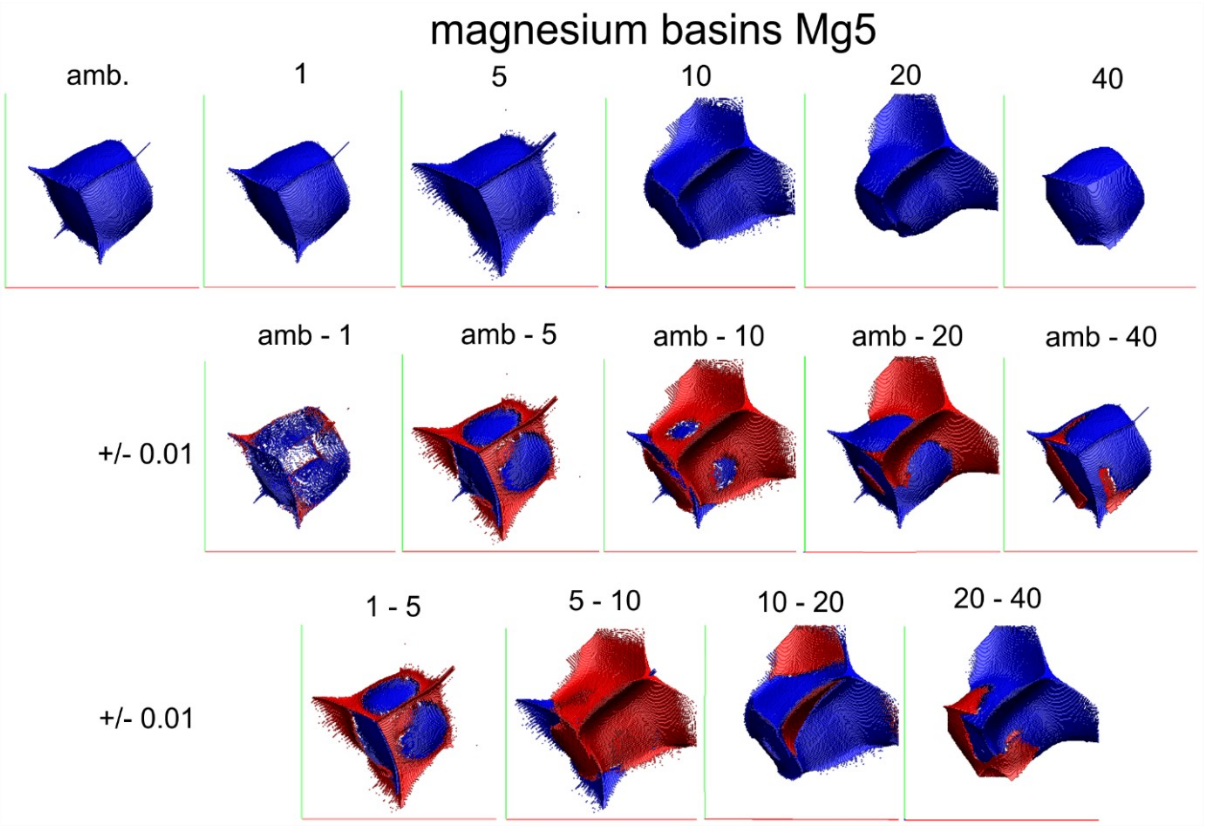

Figure S10 Atomic basins of Mg(5) cation and its changes under pressure (first row). Differences between atomic basin at ambient pressure and at other pressures – subtracted and superimposed (second row, blue - plus, red - minus). Differences between pairs of pressures.

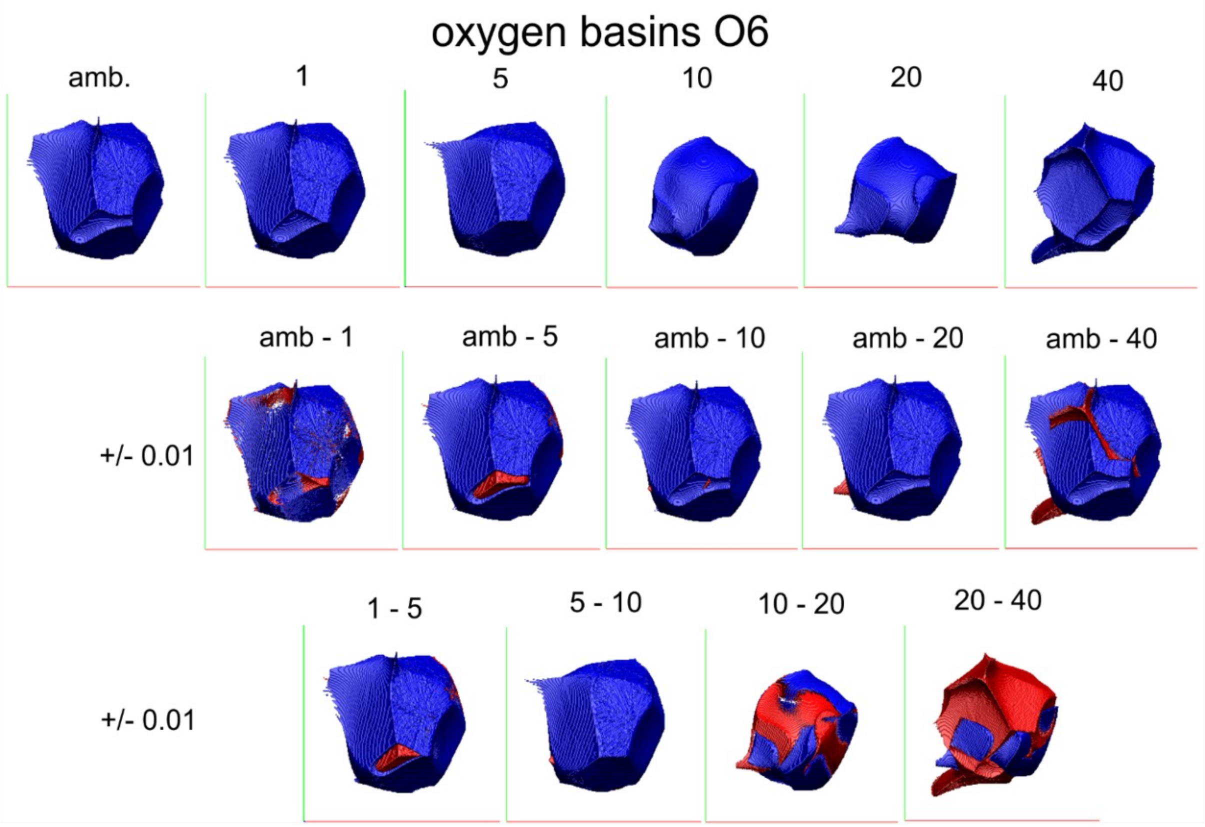

Figure S11 Atomic basins of O(6) cation and its changes under pressure (first row). Differences between atomic basin at ambient pressure and at other pressures – subtracted and superimposed (second row, blue - plus, red - minus). Differences between pairs of pressures.

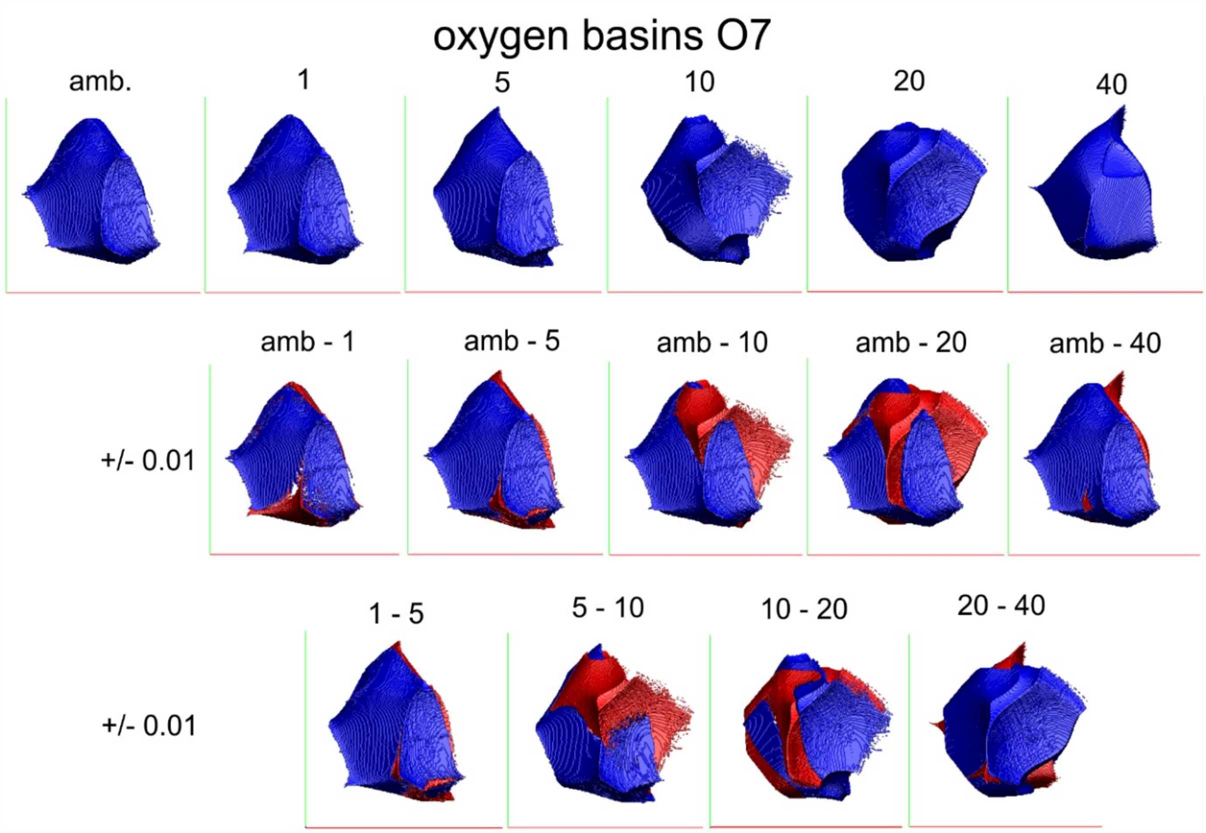

Figure S12 Atomic basins of O(7) cation and its changes under pressure (first row). Differences between atomic basin at ambient pressure and at other pressures – subtracted and superimposed (second row, blue - plus, red - minus). Differences between pairs of pressures.

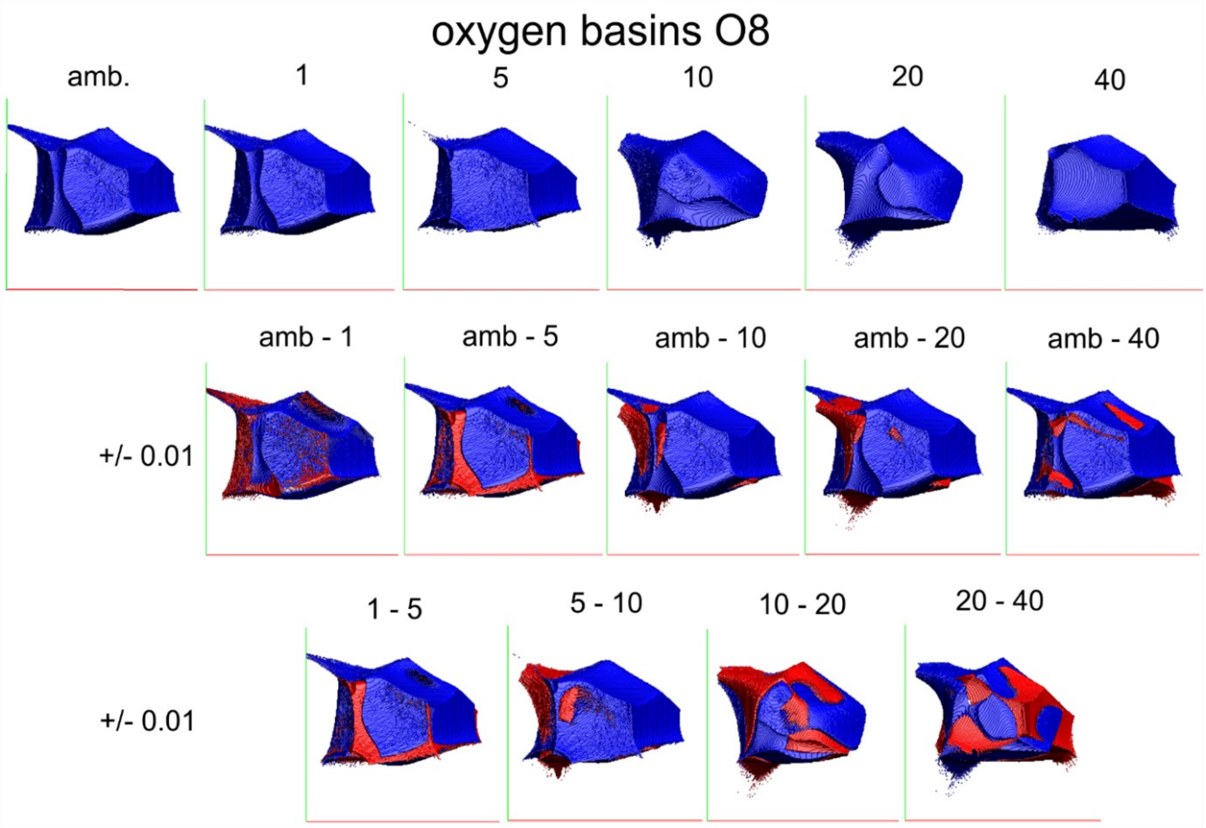

Figure S13 Atomic basins of O(8) cation and its changes under pressure (first row). Differences between atomic basin at ambient pressure and at other pressures – subtracted and superimposed (second row, blue - plus, red - minus). Differences between pairs of pressures.

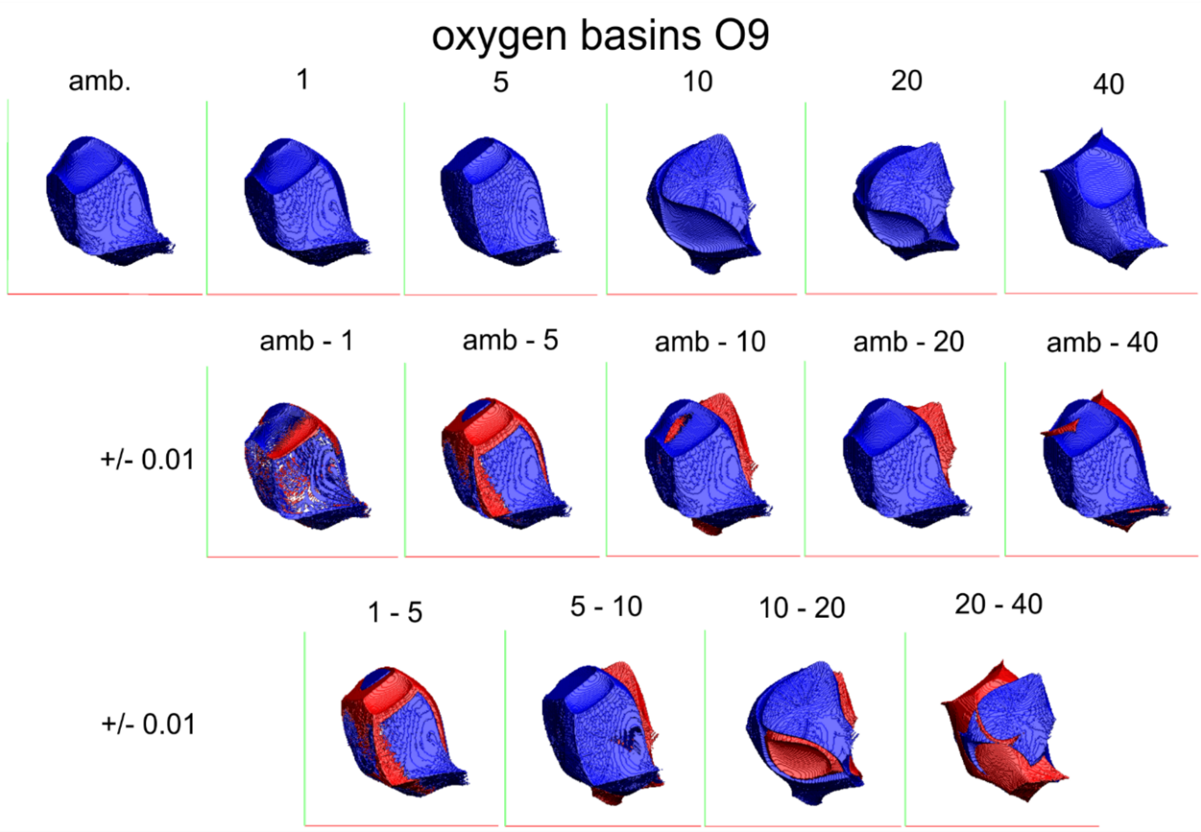

Figure S14 Atomic basins of O(9) cation and its changes under pressure (first row). Differences between atomic basin at ambient pressure and at other pressures – subtracted and superimposed (second row, blue - plus, red - minus). Differences between pairs of pressures.

Table S15 Electron density, ρ [$\bar{e}$Å^{-3}], and Laplacian, $\nabla^2\rho$ [$\bar{e}$Å^{-5}], at the (3, -1) BCPs for **Ag_exp** data with decreasing completeness.

Bond/contact	100%	90%	80%	70%	60%	50%
	ρ<br>**∇²ρ**	ρ<br>**∇²ρ**	ρ<br>**∇²ρ**	ρ<br>**∇²ρ**	ρ<br>**∇²ρ**	ρ<br>**∇²ρ**
S(3) - O(6)	1.83(8)<br>**-4.3(3)**	1.87(8)<br>**-5.7(3)**	1.86(9)<br>**-8.3(4)**	1.8(1)<br>**-5.6(4)**	1.8(1)<br>**-3.7(4)**	1.9(1)<br>**-9.5(4)**
S(3) - O(7)	2.22(7)<br>**-17.4(3)**	2.19(8)<br>**-16.4(3)**	2.22(9)<br>**-16.0(3)**	2.2(1)<br>**-18.8(3)**	2.2(1)<br>**-19.6(4)**	2.0(1)<br>**-11.8(4)**
S(3) - O(8)	2.18(7)<br>**-15.3(3)**	2.18(7)<br>**-14.5(3)**	2.21(8)<br>**-15.2(3)**	2.2(1)<br>**-13.7(3)**	2.2(1)<br>**-15.1(4)**	2.0(1)<br>**-9.8(4)**
S(3) - O(9)	2.1(1)<br>**-10.3(4)**	2.1(1)<br>**-11.1(4)**	2.1(1)<br>**-9.9(4)**	2.2(1)<br>**-13.3(5)**	2.1(1)<br>**-10.1(5)**	2.1(2)<br>**-11.1(6)**
Mg(4)…O(6)	0.44(2)<br>**2.37(4)**	0.42(3)<br>**2.80(4)**	0.40(3)<br>**3.64(5)**	0.41(1)<br>**2.60(5)**	0.50(4)<br>**1.00(6)**	0.56(7)<br>**-1.3(7)**
Mg(4)…O(7)	0.25(2)<br>**2.95(3)**	0.27(2)<br>**4.00(4)**	0.20(2)<br>**4.70(4)**	0.29(3)<br>**1.99(5)**	0.27(3)<br>**0.82(4)**	n/a<br>n/a
Mg(5)…O(8)	0.32(2)<br>**2.04(3)**	0.36(3)<br>**1.88(4)**	0.38(3)<br>**0.64(4)**	0.30(3)<br>**2.26(5)**	0.38(4)<br>**1.66(4)**	0.2(6)<br>**2.95(6)**
Mg(5)…O(9)	0.34(2)<br>**3.14(4)**	0.31(2)<br>**3.53(4)**	0.34(3)<br>**2.81(5)**	0.43(3)<br>**2.10(5)**	0.40(3)<br>**3.72(6)**	0.35(4)<br>**2.43(7)**

Table S16 Net atomic charges Ω[$\bar{e}$] and volumes V[Å^3] of atomic basins for **Ag_exp** data with decreasing completeness.

	100%	90%	80%	70%	60%	50%
	Ω<br>**V**	Ω<br>**V**	Ω<br>**V**	Ω<br>**V**	Ω<br>**V**	Ω<br>**V**
K1	+1.16<br>**19.0**	+1.02<br>**19.2**	+1.16<br>**18.1**	+0.97<br>**20.1**	+1.55<br>**19.1**	+1.20<br>**22.8**
K2	+1.12<br>**20.0**	+1.28<br>**20.6**	+1.48<br>**19.5**	+1.23<br>**21.9**	+2.16<br>**17.1**	+1.94<br>**19.3**
S3	+3.06<br>**5.6**	+3.00<br>**5.8**	+2.92<br>**5.9**	+3.23<br>**4.8**	+3.08<br>**5.3**	+3.10<br>**5.3**
Mg4	+1.53<br>**5.3**	+1.41<br>**5.6**	+1.33<br>**6.2**	+1.6<br>**5.0**	+1.52<br>**5.4**	+1.04<br>**6.1**
Mg5	+1.55<br>**5.8**	+1.63<br>**5.8**	+1.58<br>**5.8**	+1.64<br>**5.2**	+1.23<br>**5.9**	+1.75<br>**5.3**
O6	-1.03<br>**13.2**	-1.08<br>**13.3**	-0.83<br>**12.9**	-0.90<br>**12.9**	-1.30<br>**16.1**	-2.54<br>**16.7**
O7	-1.09<br>**15.4**	-1.14<br>**15.4**	-1.04<br>**15.1**	-1.14<br>**14.7**	-0.29<br>**11.0**	-0.75<br>**12.2**
O8	-1.02<br>**13.8**	-1.07<br>**14.2**	-1.36<br>**14.4**	-1.26<br>**14.4**	-1.56<br>**13.6**	-1.27<br>**15.6**
O9	-1.72<br>**17.2**	-1.48<br>**16.1**	-1.56<br>**17.0**	-1.75<br>**17.4**	-2.08<br>**19.1**	-0.39<br>**12.9**
Total*	-0.16	+0.12	-0.24	-0.08	+0.04	+1.52
Nu of electr.	**982.8**	**982.4**	**982.0**	**979.2**	**971.2**	**966.4**

*The sum of atomic charge in the unit cell

Comparison of experimental results:

Home diffractometer Ag source, RT, 1 atm	Home diffractometer Mo source, RT, 1 atm	APS, DAC, RT, 1 GPa
Normal probability plot		
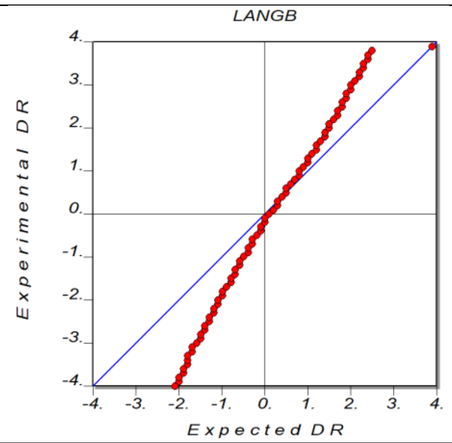	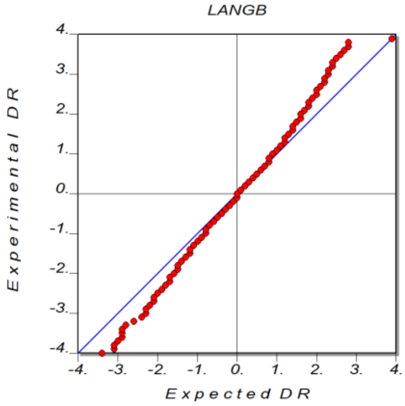	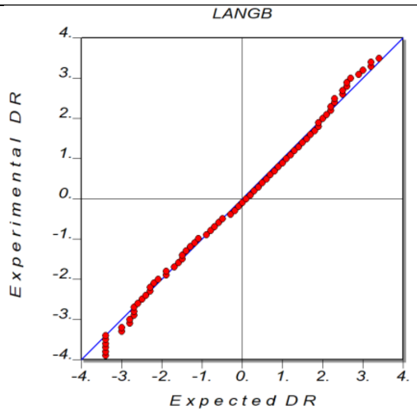
Σ(Fo²)/Σ(Fc²) as a function of sinθ/λ		
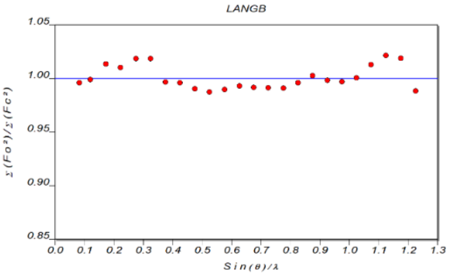	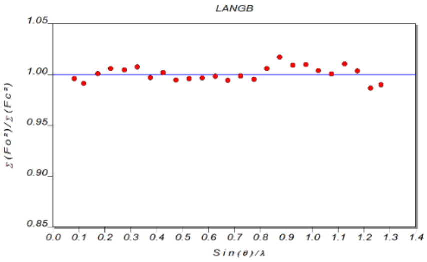	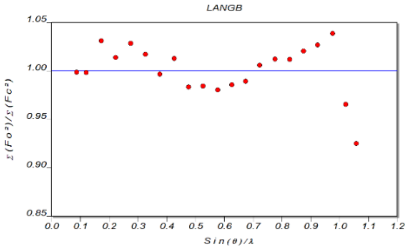

Home diffractometer Ag source, RT, 1 atm	Home diffractometer Mo source, RT, 1 atm	APS, DAC, RT, 1 GPa
Probability Distribution Histogram		
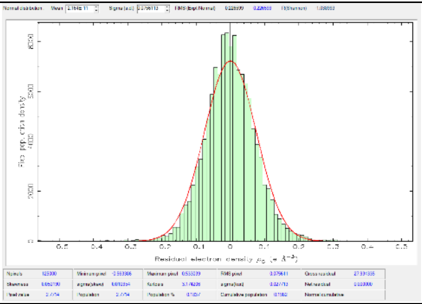	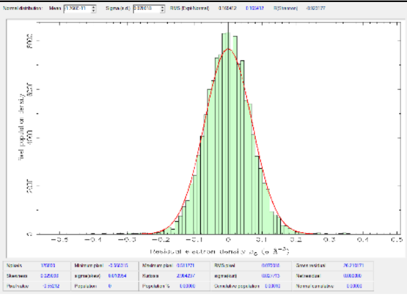	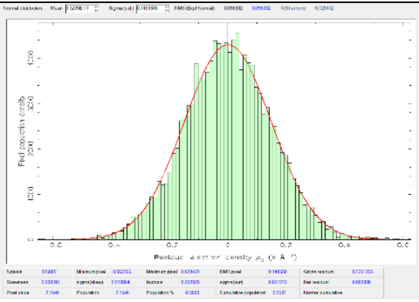
Fractal Dimension Plot		
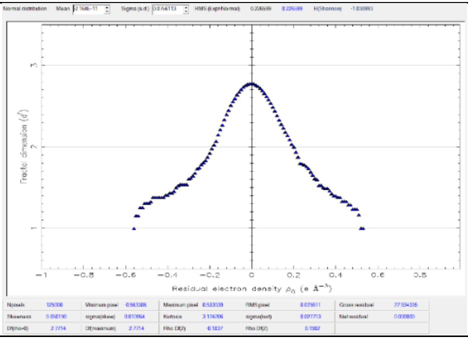	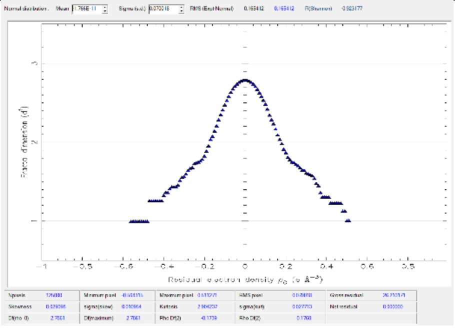	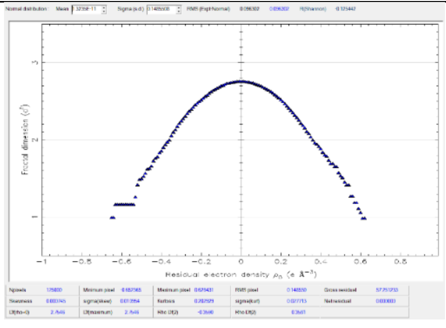

Figure S15 Normal probability plots, $\Sigma(Fo^2)/\Sigma(Fc^2)$ plots as a function of $\sin\theta/\lambda$, residual electron density distributions and fractal dimension plots for data sets discussed in this ms.
